# The SIRT3-DsbA-L-TFAM axis restrains cGAS-driven metabolic dysfunction-associated steatohepatitis in male mice

**DOI:** 10.1038/s41467-026-72395-8

**Published:** 2026-05-05

**Authors:** Li Hu, Juli Bai, Jie Wen, Hairong Luo, Dongqing Yin, Bulent T. Delibasi, Juanhong Liu, Yan Yang, Jingjing Zhang, Cynthia Ju, Alan Frazer, Lily Q. Dong, Feng Liu

**Affiliations:** 1 National Clinical Research Center for Metabolic Diseases and the Metabolic Syndrome Research Center, The Second Xiangya Hospital of Central South University, Changsha, Hunan China; 2 Health Management Center, The Second Xiangya Hospital of Central South University, Changsha, Hunan China; 3 Furong Laboratory, Changsha, Hunan China; 4 Department of Cell Systems & Anatomy, The University of Texas Health Science Center at San Antonio, San Antonio, TX USA; 5 Department of Anesthesiology, Critical Care and Pain Medicine, McGovern Medical School, University of Texas Health Science Center at Houston, Houston, TX USA; 6 Department of Endocrinology, Endocrinology Research Center, Xiangya Hospital of Central South University, Changsha, China; 7 National Clinical Research Center for Geriatric Disorders, Xiangya Hospital of Central South University, Changsha, China; 8 Department of Pharmacology, University of Texas Health San Antonio, San Antonio, TX USA

**Keywords:** Innate immunity, Metabolic disorders, Cell signalling

## Abstract

Whereas mitochondrial dysfunction is implicated in metabolic dysfunction-associated steatohepatitis (MASH), the precise underlying mechanisms remain obscure. Here, we identify the Sirtuin 3 (SIRT3)-Disulfide-bond-A oxidoreductase-like protein (DsbA-L)-Mitochondrial Transcription Factor A (TFAM) axis as a crucial suppressor for mitochondrial stress-induced cyclic GMP-AMP synthase (cGAS) activation in hepatocytes, thereby alleviating MASH in mice. SIRT3 facilitates the deacetylation of DsbA-L at lysine residues (Lys^165^, Lys^167^, and Lys^177^), which promotes the interaction between DsbA-L and TFAM, essential for the preservation of mitochondrial integrity and function. Hepatocyte-specific knockout of SIRT3 or DsbA-L in male mice promoted mitochondrial DNA release into the cytosol, resulting in activation of the cGAS pathway and exacerbation of MASH characteristics. Conversely, hepatocyte-specific knockout of cGAS or overexpression of DsbA-L mitigated diet- and SIRT3 deficiency-induced MASH progression. Our study underscores the clinical significance of targeting SIRT3 and cGAS as pivotal therapeutic avenues to inhibit the progression of MASH.

## Abstract (web summary)

This study identifies a mitochondrial protection pathway that safeguards mitochondrial DNA integrity and limits liver damage. Disrupting this pathway triggers inflammation and fibrosis, while restoring protection or inhibiting DNA sensing markedly ameliorates fatty liver disease.

## Introduction

Metabolic dysfunction-associated steatohepatitis (MASH) represents a severe form of metabolic dysfunction-associated steatotic liver disease (MASLD), characterized by hepatic inflammation, fibrosis, and cell damage alongside steatosis^1^. Whereas the precise causes of MASH pathogenesis remain incompletely understood, accumulating evidence points to mitochondrial dysfunction as a significant contributor to its development and progression^2^.

Mitochondria play vital roles in energy homeostasis^3^. An important feature of mitochondria is the presence of a prokaryotic-like genome called mitochondrial DNA (mtDNA), which is regulated differently from nuclear DNA. Unlike nuclear DNA, mtDNA is not packaged by histones but instead forms nucleoprotein complexes called nucleoids. The packaging, transcription, and maintenance of mtDNA are regulated by several nuclear encoded proteins, especially the mitochondrial transcription factor A (TFAM)^4,5^. Heterozygosity of TFAM disrupts mtDNA stability, leading to its release into the cytosol, where it triggers the cyclic GMP-AMP synthase (cGAS)-stimulator of interferon genes (STING) pathway, resulting in inflammation and Type I interferon (IFN) response^6,7^. Recent studies link cGAS-STING pathway activation to metabolic disorders, including insulin resistance, glucose intolerance, and MASLD^8–10^. However, the specific mechanisms linking liver mitochondrial dysfunction, cGAS signaling activation, and MASH progression remain elusive.

Multiple posttranslational modification mechanisms, especially acetylation, have been found to dynamically regulate the integrity and functions of mitochondria^11,12^. Among these mechanisms, mitochondrial protein acetylation is chiefly controlled by Sirtuin 3 (SIRT3), a member of the NAD^+^-dependent Sirtuin deacetylase family localized in the mitochondria. SIRT3 plays a critical role in regulating various mitochondrial functions, including the intake, production, utilization, and storage of fuel sources in response to shifts in nutrient availability^12,13^. Notably, global SIRT3 deficiency is implicated in various metabolic diseases, such as hepatic insulin resistance and hepatosteatosis^14–16^. However, intriguingly, muscle and liver-specific knockout of SIRT3 shows no significant impact on metabolic homeostasis during both chow and high-fat diet (HFD) feeding conditions^17^, raising important questions about the cell-specific functions of SIRT3 in vivo.

In this study, we uncover hepatic SIRT3 as a crucial suppressor of MASH pathogenesis. Our findings demonstrate that SIRT3 exerts its protective role by deacetylating Disulfide-bond-A oxidoreductase-like protein (DsbA-L), enabling DsbA-L to interact with TFAM, a critical step for TFAM-mediated mtDNA biosynthesis and integrity. Disruption of SIRT3 or DsbA-L expression in the mouse liver activates the cGAS-STING signaling pathway, exacerbating MASH. Conversely, liver-specific knockout of cGAS or overexpressing DsbA-L ameliorates diet- or SIRT3 deficiency-induced MASH pathogenesis. These results highlight the significance of the SIRT3-DsbA-L-TFAM cascade as a key protective mechanism, maintaining mitochondrial homeostasis and suppressing hepatic cGAS activation and MASH progression.

## Results

### Hepatic SIRT3 deficiency exacerbated metabolic abnormalities in MASH mouse models

**Fig. 1:**
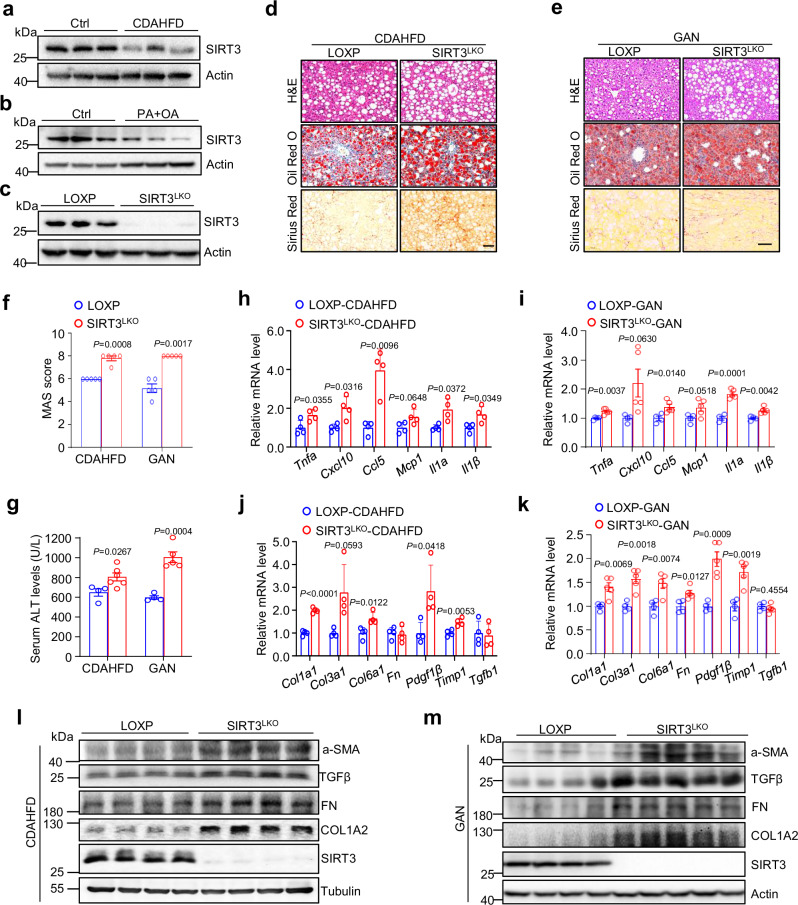
Hepatic SIRT3 deficiency exacerbates CDAHFD or GAN diet-induced MASH. **a** Decreased SIRT3 protein expression was detected in the livers of mice fed with CDAHFD for 8 weeks (*n* = 3/group from 2 independent experiments). **b** Decreased SIRT3 protein expression was detected in primary hepatocytes treated with 0.2 mM PA and 0.4 mM Oleic acid for 24 h (*n* = 3/group from 2 independent experiments). **c** Western blot showing the successful generation of SIRT3^LKO^ mice (*n* = 3/group from 3 independent experiments). **d**,** e** Hematoxylin & Eosin, Oil Red O, and Sirius red staining in liver sections of SIRT3^LKO^ and control loxp mice fed with CDAHFD for 8 weeks or GAN for 28 weeks. Scale bars, 100 μm. **f** MAS grading score was analyzed in livers of CDAHFD- or GAN-fed SIRT3 ^LKO^ (*n* = 5) and control loxp (*n* = 5) mice from 2 independent experiments. **g** Serum ALT concentrations in the indicated groups of mice. (control loxp, *n* = 4; SIRT3^LKO^, *n* = 6 from 2 independent experiments). **h**, **i** Relative mRNA levels of inflammatory genes in the liver of CDAHFD- or GAN-fed SIRT3^LKO^ mice compared to control mice (control loxp, *n* = 4; SIRT3^LKO^, *n* = 2 independent experiments). **j**–**k** Relative mRNA levels of fibrosis-related genes in the livers of CDAHFD- or GAN-fed SIRT3^LKO^ and control loxp mice. (control loxp, *n* = 4; SIRT3^LKO^, *n* = 6 from 2 independent experiments). **l**,** m** Protein levels of fibrosis-related genes in the livers of CDAHFD- or GAN-fed SIRT3^LKO^ and control loxp mice. In all statistical plots, data are presented as mean ± SEM. Statistical significance for (**f**–**k**) was determined by an unpaired, two-sided *t* test. Source data are provided as a Source Data file.

To investigate the role of hepatic SIRT3 in the development of diet-induced liver disease, we first examined whether its expression is altered under MASH conditions. Hepatic SIRT3 was markedly reduced in mice fed a choline-deficient, L-amino acid-defined, high-fat diet (CDAHFD) (Fig. 1a) and in primary hepatocytes treated with palmitic acid (PA) and oleic acid (OA), which mimic lipotoxic stress (Fig. 1b). To assess the functional impact, liver-specific SIRT3 knockout (SIRT3^LKO^) mice (Fig. 1c) were challenged with CDAHFD, Gubra-Amylin (GAN), and methionine–choline-deficient (MCD) diets. Across all models, SIRT3^LKO^ livers showed pronounced lipid accumulation and fibrosis (Fig. 1d, e and Supplementary Fig. 1a, b), accompanied by significantly higher metabolic dysfunction-associated steatotic liver disease activity score (MAS-score), which reflects the severity of steatosis, ballooning degeneration, and lobular inflammation (Fig. 1f and Supplementary Fig. 1c) and collagen deposition (Supplementary Fig. 1d). Serum alanine aminotransferase (ALT) levels were elevated, indicating impaired liver function (Fig. 1g). Moreover, SIRT3^LKO^ livers displayed strong upregulation of inflammatory (*Tnfα, Cxcl10, Ccl5, Il1α, Il1β*) and fibrotic (*Col1a1, Col3a1, Col6a1, Fn, Pdgf1β, Timp1*) genes, as well as increased αSMA, TGFβ, FN, and COL1A2 proteins (Fig. 1h–m and Supplementary Fig. 1e–g). Collectively, these results identify hepatic SIRT3 as a key protector against MASH development and progression.

### SIRT3 deficiencies are associated with activation of the cGAS-STING signaling in liver

**Fig. 2:**
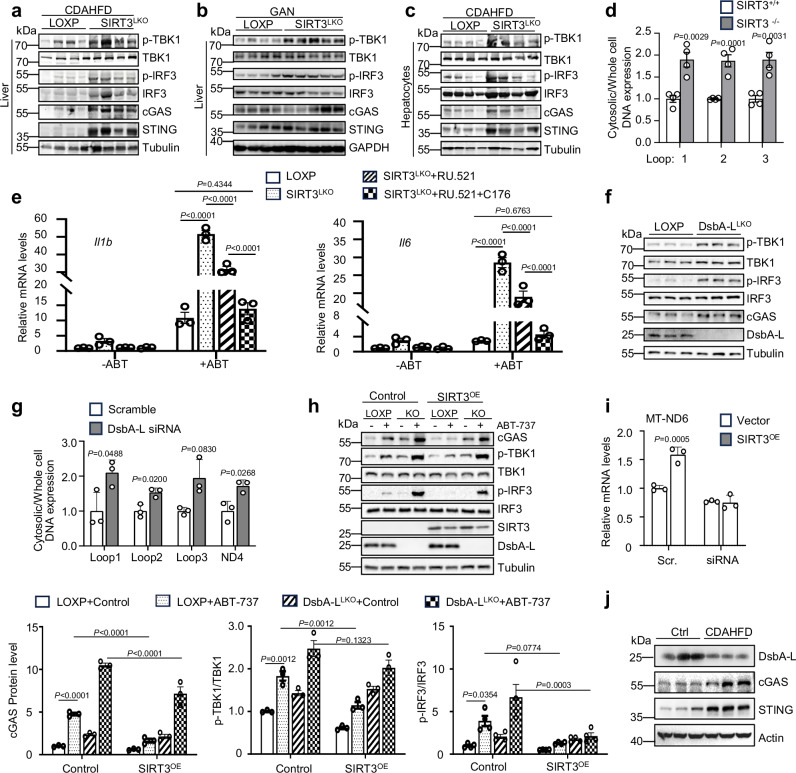
SIRT3 or DsbA-L deficiency led to mitochondrial DNA release-induced activation of the cGAS signaling. **a**,** b** Increased cGAS signaling is observed in the livers of SIRT3^LKO^ mice fed with CDAHFD (*n* = 4 mice/group) or GAN (control loxp, *n* = 4; SIRT3 ^LKO^, *n* = 5) diet from 2 independent experiments. **c** The cGAS-STING signaling pathway in primary hepatocytes isolated from CDAHFD-fed SIRT3^LKO^ and control mice (*n* = 4 mice/group from 2 independent experiments). **d** Increased mtDNA release in the hepatocytes of MCD-fed SIRT3 knockout mice from 3 independent experiments (control loxp, *n* = 4; SIRT3 ^LKO^, *n* = 4). **e** The mRNA levels of inflammatory genes in SIRT3 knockout or control loxp primary hepatocytes pre-treated with cGAS inhibitor RU.521 (5 μM) or STING inhibitor C176 (5 μM) for 2 h, followed by ABT-737 treatment (10 μM) for 24 h (*n* = 3/group from 3 independent experiments). **f** Increased cGAS signaling is observed in the primary mouse hepatocytes of DsbA-L^LKO^ mice fed with MCD (*n* = 3 mice/group from 3 independent experiments). **g** mtDNA release in DsbA-L suppressed mouse hepatocytes was determined by RT-qPCR (*n* = 3/group from 2 independent experiments). **h** Immunoblot and its quantification analysis of DsbA-L-deficient hepatocytes overexpressing SIRT3 or the control by stable transfection, followed with or without 10 μM ABT-737 treatment for 16 h (3 independent experiments). **i** mtDNA content in scramble and DsbA-L suppressed mouse hepatocytes overexpressing control or SIRT3 plasmid (*n* = 3/group from 2 independent experiments). **j** Immunoblot analysis indicated a reduction of DsbA-L and an increase of cGAS and STING protein levels in livers from CDAHFD fed C57BL/6 mice (*n* = 3 mice/group from 2 independent experiments). In all statistical plots, data are presented as mean ± SEM. Statistical significance for (**d**, **g**, **i**) was determined by an unpaired, two-sided *t* test, and for (**e**, **h**) was determined by Two-way ANOVA with Tukey’s multiple comparisons test. Source data are provided as a Source Data file.

Inflammation is a key driver of liver fibrosis and MASH. Because mitochondrial stress activates cGAS-STING signaling to promote inflammation^10,18^, we asked whether SIRT3 deficiency, known to induce mitochondrial stress^19^, affects this pathway in the liver. Compared with wild-type controls, SIRT3^LKO^ mice fed CDAHFD, GAN, or MCD diets showed markedly elevated hepatic expression of cGAS and STING, along with increased phosphorylation of TBK1 and IRF3, two downstream effectors of this pathway (Fig. 2a, b and Supplementary Fig. 1h). Isolated hepatocytes from SIRT3^LKO^ mice similarly displayed increased cGAS and STING expression and enhanced TBK1 and IRF3 phosphorylation (Fig. 2c), accompanied by significant cytosolic mtDNA release (Fig. 2d).

To determine whether cGAS-STING mediates SIRT3 deficiency-induced inflammation, SIRT3-deficient hepatocytes were pretreated with the cGAS inhibitor RU.521 or the STING inhibitor C176 before ABT-737 exposure. ABT-737, a Bcl-2 inhibitor, induces mtDNA release and activates cGAS–STING signaling^20^. Blocking either cGAS or STING markedly reduced ABT-737-induced *Il6* and *Il1β* expression (Fig. 2e), linking SIRT3 loss to cGAS–STING activation in hepatocytes.

Although STING expression is typically low in hepatocytes, it can be upregulated under pathological conditions^21–25^. To exclude immune cell contamination, we analyzed purified hepatocytes from STING^Loxp^ and hepatocytes-specific STING knockout (STING^LKO^) mice. RT-PCR analysis confirmed *sting* mRNA expression in hepatocytes and its loss in knockout cells (Supplementary Fig. 2a). Transcriptomic profiling identified 97 upregulated and 218 downregulated genes in palmitate-treated STING^LKO^ hepatocytes versus controls, with KEGG analysis showing reduced inflammatory pathways, including TNF, NF-κB, and cytokine-cytokine receptor signaling (Supplementary Fig. 2b–d). These findings demonstrate that STING mediates inflammatory signaling within hepatocytes.

SIRT3 is a mitochondrial deacetylase that modulates the acetylation of various mitochondrial proteins, including DsbA-L^12^. Given that DsbA-L suppresses cGAS-STING signaling in adipocytes^10,20^, we examined whether its downregulation contributes to SIRT3 deficiency-induced pathway activation. DsbA-L loss in hepatocytes markedly increased TBK1 and IRF3 phosphorylation and promoted mtDNA cytosolic release (Fig. 2f, g). Moreover, DsbA-L deficiency blunted the inhibitory effect of SIRT3 on ABT-737-induced cGAS-STING activation (Fig. 2h) and reduced the SIRT3-driven increase in mtDNA synthesis (Fig. 2i). Consistently, CDAHFD-fed mouse livers showed reduced DsbA-L and elevated cGAS and STING expression (Fig. 2j). Together, these results identify DsbA-L as a key mediator of SIRT3-dependent suppression of cGAS-STING signaling in hepatocytes.

### SIRT3 regulates the interaction between DsbA-L and TFAM

**Fig. 3:**
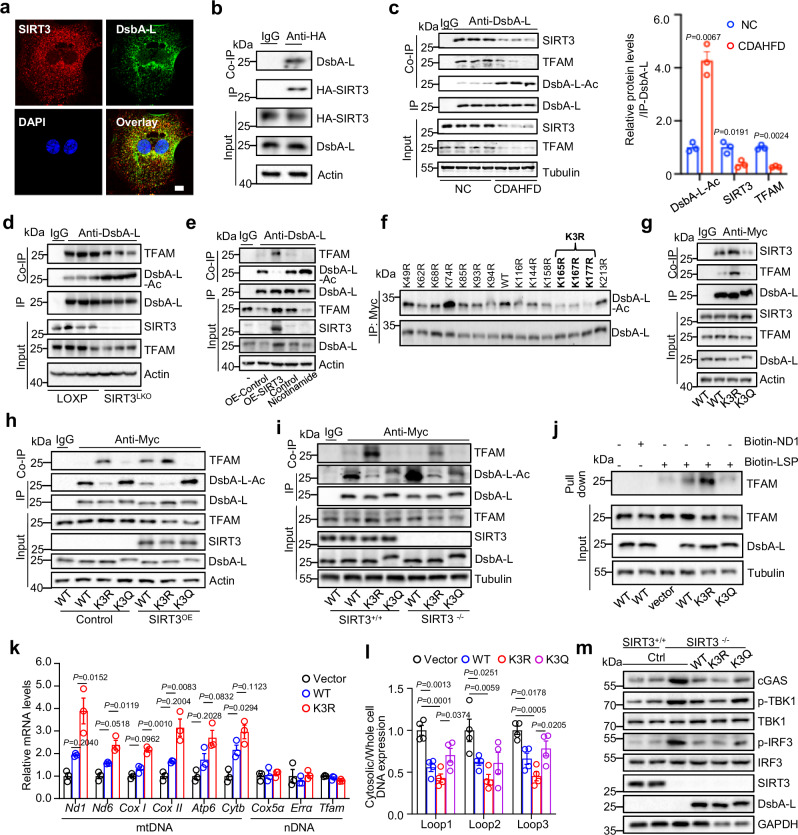
SIRT3 regulates the interaction of DsbA-L with TFAM and the activation of cGAS signaling through deacetylating DsbA-L. **a** Co-localization of DsbA-L and SIRT3 in mouse hepatocytes was determined by confocal immunofluorescence experiments. Scale bar, 10 μm. **b** The interaction of DsbA-L and SIRT3 in mouse hepatocytes was determined by co-immunoprecipitation assay. The interaction of DsbA-L with SIRT3 or TFAM in liver tissues from normal diet (**c**) or CDAHFD (**d**) -fed SIRT3^LKO^ and loxp control mice was determined by co-immunoprecipitation assay. (normal diet, *n* = 4; CDAHFD, *n* = 3). **e** The acetylation level of DsbA-L and the interaction of DsbA-L with TFAM were determined in primary hepatocytes transiently overexpressing SIRT3 or a control plasmid, or treated with Nicotinamide. **f** Acetylation of Myc-tagged wild-type (WT) DsbA-L and DsbA-L mutants (with lysine residues mutated to arginine) in DsbA-L-suppressed mouse hepatocytes was detected by Western blot. **g**–**i** Interactions of WT, K3R, and K3Q DsbA-L with SIRT3 or TFAM in DsbA-L knockout primary hepatocytes (with or without transient SIRT3 overexpression) and SIRT3 knockout primary hepatocytes were determined by co-immunoprecipitation assay. **j** The binding affinity of TFAM to LSP DNA was detected by Biotin-LSP pull-down assay in DsbA-L knockout primary hepatocytes overexpressing WT or K3R or K3Q of DsbA-L. **k** The expression of mtDNA and nuclear DNA (nDNA) in DsbA-L knockout primary hepatocytes overexpressing WT or K3R of DsbA-L was determined by RT-qPCR (*n* = 3/group). **l** mtDNA release in SIRT3 knockout primary hepatocytes overexpressing WT or K3R or K3Q of DsbA-L was determined by RT-qPCR (*n* = 4/group). **m** Expression and/or phosphorylation of components in the cGAS pathway were detected by Western blot in SIRT3 knockout primary hepatocytes overexpressing WT or K3R or K3Q of DsbA-L. **a**, **b,** **e,** **f**–**j**, **m** are from 3 independent experiments, **c**, **k**, **l**, are from 2 independent experiments. In all statistical plots, data are presented as mean ± SEM. Statistical significance for (**c**) was determined by an unpaired, two-sided *t* test, and for (**k,** **l**) was determined by One-way ANOVA with Tukey’s multiple comparisons test. Source data are provided as a Source Data file.

To further validate the earlier proteomic finding that DsbA-L is a target of SIRT3^12^, we checked if DsbA-L colocalizes or interacts with SIRT3. By using confocal imaging and co-immunoprecipitation experiments, we found that DsbA-L was colocalized (Fig. 3a) and interacted (Fig. 3b) with SIRT3 in mouse hepatocytes. To understand the impact of DsbA-L on SIRT3-mediated mtDNA homeostasis, we explored whether DsbA-L interacts with TFAM, a master regulator of mtDNA packaging and transcription. Co-immunoprecipitation experiments demonstrated that DsbA-L forms a complex with SIRT3 and TFAM in mouse liver. This interaction was notably reduced in the liver of CDAHFD-fed mice, coinciding with increased DsbA-L acetylation (Fig. 3c). In addition, CDAHFD-fed SIRT3^LKO^ mice displayed an even greater reduction in DsbA-L interaction with TFAM, which was also associated with elevated DsbA-L acetylation (Fig. 3d). Conversely, overexpression of SIRT3 in mouse hepatocytes substantially enhanced the interaction between DsbA-L and TFAM, while simultaneously reducing DsbA-L acetylation levels (Fig. 3e). These findings consistently demonstrated that SIRT3 plays a pivotal role in regulating the interaction between DsbA-L and TFAM. To reinforce this observation, SIRT3 activity was inhibited using nicotinamide in mouse hepatocytes, which led to a considerable increase in DsbA-L acetylation levels and a corresponding decrease in its association with TFAM (Fig. 3e). These results highlight the cell-autonomous role of SIRT3 in modulating the interaction between DsbA-L/TFAM.

### Deacetylation facilitates DsbA-L interaction with TFAM and improves mitochondrial function

To ascertain the impact of DsbA-L deacetylation on its binding to TFAM, we generated myc-tagged DsbA-L mutants wherein potential SIRT3 targeting sites (Lys^49^, Lys^62^, Lys^68^, Lys^74^, Lys^85^, Lys^93^, Lys^94^, Lys^116^, Lys^144^, Lys^158^, Lys^165^, Lys^167^, Lys^177^, and Lys^213^)^26^ were individually mutated to arginine using site-directed mutagenesis. Notably, mutations at Lys^165^, Lys^167^, and Lys^177^ to arginine significantly diminished DsbA-L acetylation in mouse hepatocytes (Fig. 3f and Supplementary Fig. 3a, b). To further explore the importance of DsbA-L deacetylation, we created DsbA-L mutants by simultaneously mutating Lys^165^, Lys^167^, and Lys^177^ to either arginine (DsbA-L^K3R^) to prevent acetylation or to glutamine (DsbA-L^K3Q^) to mimic acetylation. Our results showed that the DsbA-L K3R mutant strengthened its interactions with both SIRT3 and TFAM in primary hepatocytes, whereas the K3Q mutant markedly weakened these interactions relative to wild-type DsbA-L (Fig. 3g), indicating that DsbA-L acetylation critically regulates these protein associations. In line with these observations, overexpression of SIRT3 markedly enhanced the DsbA-L-TFAM interaction while reducing DsbA-L acetylation (Fig. 3h). The stimulatory effect was abolished when critical acetylation sites (Lys^165^, Lys^167^, and Lys^177^) were mutated to glutamine. Conversely, SIRT3 deletion increased DsbA-L acetylation and weakened its binding to TFAM, whereas the K3R mutant remained largely unaffected (Fig. 3i), identifying these lysine residues as primary SIRT3 deacetylation targets essential for TFAM association.

To determine the functional consequence, we examined TFAM binding to the light-strand promoter (LSP) of the mitochondrial D-loop, which drives mtDNA synthesis^27–29^. TFAM occupancy at the LSP was elevated in hepatocytes expressing wild-type or, more prominently, K3R DsbA-L, but not the K3Q mutant (Fig. 3j). Consistently, both wild-type and K3R DsbA-L enhanced mtDNA biosynthesis without affecting nuclear DNA (Fig. 3k), indicating a specific role in mtDNA replication. Furthermore, overexpression of wild-type or K3R DsbA-L suppressed mtDNA release and cGAS-STING activation in DsbA-L-deficient (Fig. 3l) or ABT-737-treated (Supplementary Fig. 4a, b) primary hepatocytes. Similar effects were observed in SIRT3-deficient cells (Fig. 3m), highlighting DsbA-L deacetylation as a key mediator of the SIRT3/TFAM axis in restraining cGAS signaling. The K3Q mutant also partially reduced mtDNA release, suggesting an additional acetylation-independent protective function. Finally, overexpression of wild-type or K3R DsbA-L markedly improved mitochondrial function in DsbA-L- or SIRT3-deficient hepatocytes, as evidenced by increased oxygen consumption, ATP production, and membrane potential, along with reduced ROS and lipid accumulation (Supplementary Figs. 4c–g, 5a–e). In contrast, the K3Q mutant had no such effects. Collectively, these findings demonstrate a cell-autonomous role of SIRT3 in suppressing cGAS activation, primarily through DsbA-L deacetylation.

### Liver-specific knockout of DsbA-L aggravates MASH in mice

**Fig. 4:**
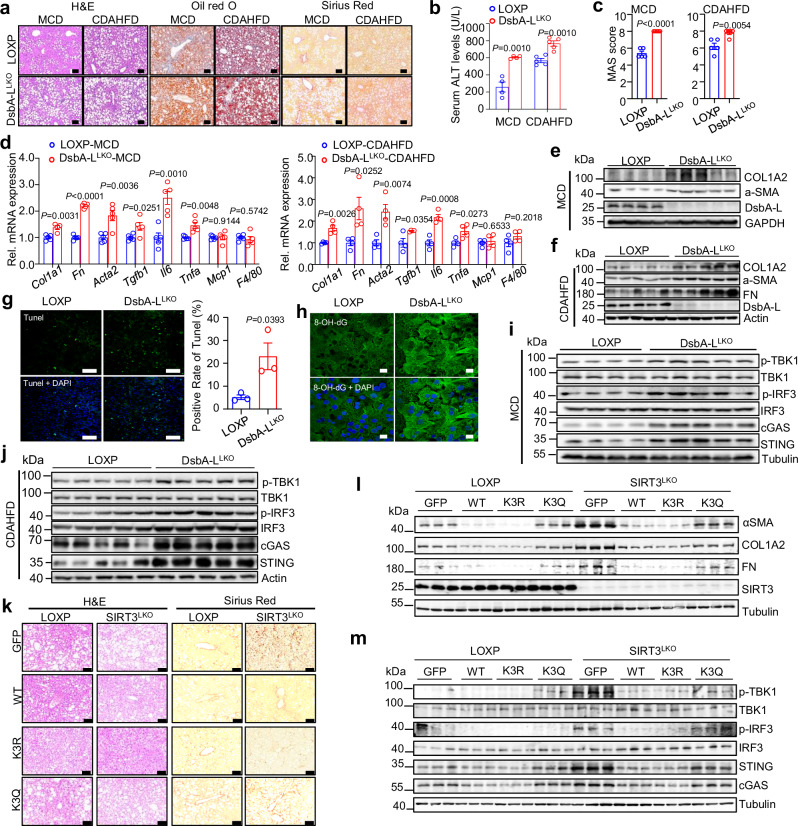
Hepatic DsbA-L deficiency exacerbates, whereas overexpression alleviates liver steatosis and fibrosis. **a** Hematoxylin and Eosin, Oil red O, and Sirius red staining of liver sections from DsbA-L^LKO^ and control wild-type mice fed an MCD or CDAHFD diet for 8 weeks. Scale bars, 100 μm. **b** Serum ALT levels in the indicated groups of mice were measured. (MCD, *n* = 4/group; CDAHFD, *n* = 5/group). **c** MAS score analysis of liver sections from DsbA-L^LKO^ and control wild-type mice fed an MCD or CDAHFD diet for 8 weeks (*n* = 5/group). **d** The mRNA expression of hepatic fibrosis genes and inflammatory genes were measured by RT-qPCR from DsbA-L^LKO^ and control wild-type mice fed an MCD or CDAHFD diet for 8 weeks (MCD, *n* = 5/group; CDAHFD, *n *= 4/group). **e**, **f** The protein expression of fibrosis genes in livers from DsbA-L^LKO^ and control wild-type mice fed an MCD (loxp, *n* = 4/group; DsbA-L^LKO^, *n* = 5/group) or CDAHFD (*n* = 5/group) diet for 8 weeks. Tunel staining (**g**) (*n* = 3/group) and 8-OH-dG staining (**h**) (*n* = 3/group) of liver sections from DsbA-L^LKO^ and control wild-type mice fed an MCD diet for 8 weeks. Scale bars, 200 μm for Tunel staining, 20 μm for 8-OH-dG staining. **i**, **j** The cGAS signaling were examined in livers from loxp and DsbA-L^LKO^ mice fed with an MCD diet or CDAHFD for 8 weeks (MCD, loxp, *n* = 4; DsbA-L^LKO^, *n* = 5; CDAHFD, *n* = 5/group). **k** Hematoxylin & Eosin and Sirius red staining in liver sections of CDAHFD-fed SIRT3^LKO^ and loxp mice i.v. injected with the indicated virus (*n* = 3/group). Scale bars, 100 μm. **l** Relative protein levels of fibrosis-related genes in the livers of CDAHFD-fed SIRT3 ^LKO^ and loxp mice (*n* = 3/group). **m** cGAS signaling activation were determined in the livers of CDAHFD-fed SIRT3 ^LKO^ and loxp mice (*n* = 3/group). All animal studies are from 2 independent experiments. In all statistical plots, data are presented as mean ± SEM. Statistical significance for (**b**, **c**, **d**, **g**) was determined by an unpaired, two-sided *t* test. Source data are provided as a Source Data file.

Based on the finding that hepatic SIRT3 suppresses MASH, we postulated that hepatic DsbA-L may be a suppressor of MASH progression. To test this possibility, liver-specific DsbA-L knockout mice (DsbA-L^LKO^) and control mice were fed an MCD or CDAHFD diet for 8 weeks. The livers of DsbA-L^LKO^ mice displayed increased collagen fibers, lipid droplets (Fig. 4a), and elevated serum ALT levels compared to those in loxp control mice under both diet conditions (Fig. 4b). The MAS score was significantly higher in the liver of DsbA-L^LKO^ mice compared to that in floxed wild-type control mice (Fig. 4c). Consistent with these findings, the mRNA levels of several fibrogenic markers such as *Col1a1, Fn, Acta2* (Fig. 4d) as well as protein levels of COL1A2, αSMA or FN (Fig. 4e, f) were significantly increased in the liver of DsbA-L^LKO^ mice compared those in loxp control littermates. Moreover, inflammatory genes such as *Il6* or *TNFα* were also significantly increased in the liver of DsbA-L^LKO^ mice compared with those in loxp control littermates (Fig. 4d). Tunel staining showed increased numbers of apoptotic cells in the liver of DsbA-L^LKO^ mice compared to those in control mice (Fig. 4g). In addition, a notably increased 8-OH-dG was detected in livers of DsbA-L^LKO^ mice, indicating an elevated mtDNA oxidative damage (Fig. 4h). Increased TBK1 and IRF3 phosphorylation as well as cGAS and STING expression were also detected in hepatocytes isolated from DsbA-L^LKO^ mice compared to those from control mice (Fig. 4i, j). Taken together, these findings provide further evidence on a functional link between DsbA-L and SIRT3 in MASH pathogenesis.

### DsbA-L^K3R^ but not DsbA-L^K3Q^ mutant alleviates SIRT3 deficiency-induced MASH pathogenesis in mice

To test if DsbA-L acetylation mediates the suppressing effect of SIRT3 on MASH, we injected adeno-associated viruses (AAV) encoding GFP or wild-type, K3R, or K3Q mutant of DsbA-L in hepatocytes of SIRT3^LKO^ mice through the tail vein. After 12 weeks of CDAHFD diet feeding, SIRT3^LKO^ mice expressing GFP exhibited increased lipid accumulation and fibrosis compared to that measured in loxp control mice expressing GFP (Fig. 4k). Overexpression of either wild-type or the K3R mutant, but not the K3Q mutant, of DsbA-L, markedly suppressed SIRT3 deficiency-induced lipid accumulation and fibrosis in SIRT3^LKO^ mice (Fig. 4k). The protein levels of fibrogenic markers, such as αSMA, COL1A2, and FN were all significantly suppressed in SIRT3^LKO^ mice overexpressing wild-type and the K3R but not K3Q mutant DsbA-L (Fig. 4l). Compared to GFP overexpressed SIRT3^LKO^ mice, the activation of the cGAS-STING signaling was also suppressed in SIRT3^LKO^ mice overexpressing either wild-type or K3R mutant of DsbA-L (Fig. 4m). Consistent with these findings, the mRNA levels of several fibrogenic markers such as *Col1a1*, *Col3a1*, *Col6a1*, *Pdgfb*, *Timp1* (Supplementary Fig. 5f–k), and inflammatory markers such as *Tnfa, Cxcl10*, *Ccl5*, *Mcp1*, *Il1a* and *Il1b* (Supplementary Fig. 5l–q) were all significantly reduced in the liver of SIRT3^LKO^ mice overexpressing wild-type or K3R mutant of DsbA-L compared to those in SIRT^LKO^ mice overexpressing the GFP. These in vivo results indicate that DsbA-L deacetylation is critical to mediate the protection effect of SIRT3 on MASH.

### Knockout of cGAS in hepatocytes protects MASH development and pathogenesis in mice

**Fig. 5:**
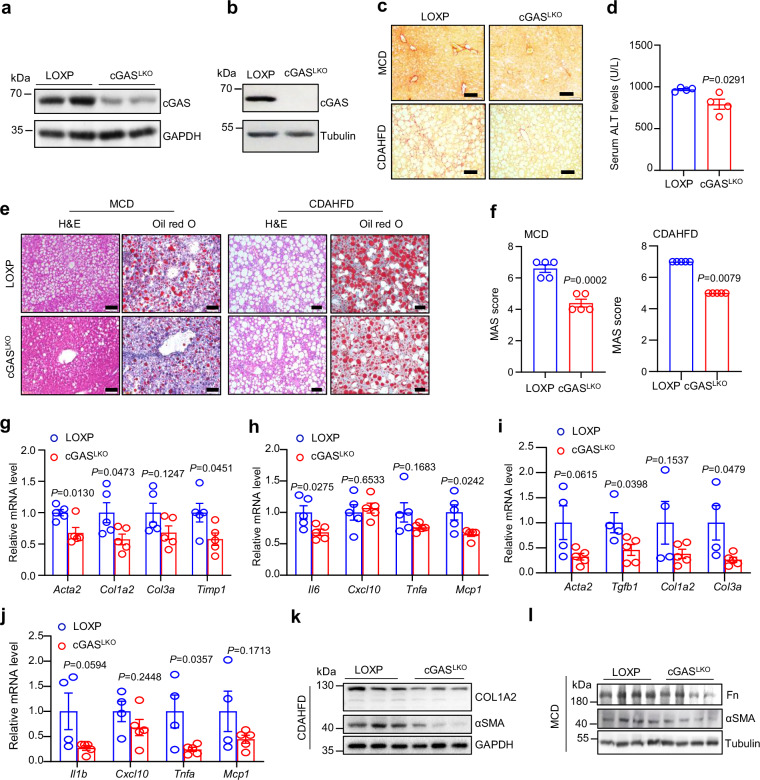
Knockout of cGAS in mouse hepatocytes protects MASH. **a**, **b** Successful knockout of cGAS in mice was confirmed by western blot in liver tissue (*n* = 2/group) (**a**) and primary hepatocytes (*n* = 1/group) (**b**) from 3 independent experiments. **c** Sirius red staining in liver sections of MCD- or CDAHFD-fed cGAS^LKO^ and control loxp mice. Scale bars, 100 μm for MCD, 50 μm for CDAHFD. **d** Serum ALT levels in CDAHFD-fed cGAS^LKO^ and control loxp mice (*n* = 4 mice/group from 2 independent experiments). **e** Hematoxylin & Eosin (H&E) and Oil red O staining in liver sections of MCD- or CDAHFD-fed cGAS^LKO^ and control loxp mice. Scale bars, 100 μm for H&E staining in the MCD group, 50 μm for the others. **f** MAS score was analyzed in liver sections from MCD- or CDAHFD -fed cGAS^LKO^ and control loxp mice. Relative mRNA levels of fibrosis- (**g**) or inflammation- (**h**) related genes in the livers of CDAHFD-fed cGAS ^LKO^ and control loxp mice (*n* = 5 mice/group from 2 independent experiments). Relative mRNA levels of fibrosis (**i**) or inflammation (**j**) -related genes in the livers of MCD-fed cGAS ^LKO^ (*n* = 4) and control loxp mice (*n* = 5) from 2 independent experiments. Relative protein levels of fibrosis- or inflammation-related genes in the livers of CDAHFD (**k**) (*n* = 3 mice/group from 2 independent experiments) or MCD-fed (**l**) (*n* = 4 mice/group from 2 independent experiments) cGAS ^LKO^ and control loxp mice. In all statistical plots, data are presented as mean ± SEM. Statistical significance for (**d**, **f**–**j**) was determined by an unpaired, two-sided *t* test. Source data are provided as a Source Data file.

To assess the role of hepatic cGAS in MASH, we generated liver-specific cGAS knockout mice (cGAS^LKO^) by crossing cGAS floxed mice with Alb-cre mice (Fig. 5a, b). Because the liver contains multiple cell types and cGAS is highly expressed in immune cells, total liver cGAS protein was reduced but not eliminated (Fig. 5a). However, complete loss of cGAS was confirmed in hepatocytes isolated from the cGAS^LKO^ mice (Fig. 5b). After 8 weeks of MCD or CDAHFD feeding, cGAS^LKO^ mice showed markedly less hepatic fibrosis and inflammation than floxed controls (Fig. 5c). Serum ALT levels were significantly lower (Fig. 5d), and H&E and Oil Red O staining revealed fewer lipid droplets, consistent with reduced MASH scores (Fig. 5e, f). Correspondingly, hepatic mRNA levels of fibrogenic genes (*Tgfb1, Acta2, Col1a2, Col3a1, Timp1*) and inflammatory genes (*Il6, Tnfa, Mcp1*) were significantly decreased (Fig. 5g–j). Protein levels of COL1A2, αSMA, and fibronectin were also reduced in cGAS^LKO^ livers (Fig. 5k, l).

### Hepatic cGAS knockout alleviates SIRT3 deficiency-induced MASH pathogenesis in mice

**Fig. 6:**
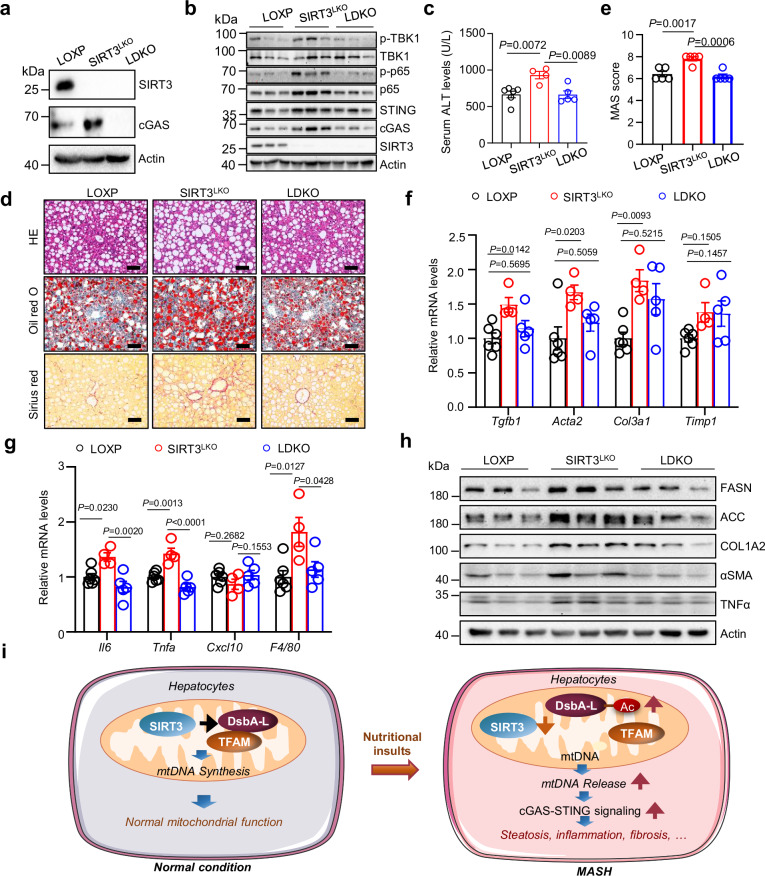
Hepatic cGAS knockout alleviates SIRT3 deficiency-induced MASH pathogenesis in mice. **a** Successful generation of cGAS-SIRT3 double knockout (SIRT3/cGAS^LDKO^) mice was confirmed by western blot in purified hepatocytes (from 3 independent experiments). **b** cGAS signaling activation were determined in the livers of CDAHFD-fed SIRT3/cGAS^LDKO^ mice, SIRT3^LKO^ mice and control loxp mice (*n* = 3 mice/group from 2 independent experiments). **c** Serum ALT levels in CDAHFD-fed SIRT3/cGAS^LDKO^ mice (*n* = 5), SIRT3^LKO^ mice (*n *= 4), and control loxp mice (*n *= 6) from 2 independent experiments. **d** Hematoxylin & Eosin, Oil red O, and Sirius red staining of liver sections from CDAHFD-fed SIRT3/cGAS^LDKO^ mice, SIRT3^LKO^ mice and control loxp mice. Scale bars, 50 μm. **e** MAS score analysis of liver sections from CDAHFD-fed SIRT3/cGAS^LDKO^ mice, SIRT3^LKO^ mice and control loxp mice (*n* = 5 mice/group from 2 independent experiments). Relative mRNA levels of fibrosis- (**f**) or inflammation- (**g**) related genes in the livers of CDAHFD-fed SIRT3/cGAS^LDKO^ mice, SIRT3^LKO^ mice and control loxp mice (*n* = 6 for loxp, *n* = 4 for SIRT3^LKO^, *n* = 5 for SIRT3/cGAS^LDKO^ from 2 independent experiments). **h** Relative protein levels of lipid metabolism, fibrosis, and inflammation-related genes in the livers of CDAHFD-fed SIRT3/cGAS^LDKO^ mice, SIRT3^LKO^ mice and control loxp mice (*n* = 3/group from 2 independent experiments). **i** A working model illustrating the regulation of mitochondrial homeostasis by the SIRT3-DsbA-L-TFAM cascade in MASH. In all statistical plots, data are presented as mean ± SEM. Statistical significance for (**c**,** e**, **f**, **g**) was determined by One-way ANOVA with Tukey’s multiple comparisons test. Source data are provided as a Source Data file.

To determine if hepatic cGAS-STING signaling activation contributes to SIRT3 deficiency-induced MASH, we generated liver-specific SIRT3/cGAS double knockout mice (SIRT3/cGAS^LDKO^) (Fig. 6a). After 8 weeks of CDAHFD diet feeding, these mice displayed markedly reduced phosphorylation of TBK1 and p65 and lower expression of cGAS and STING compared with SIRT3^LKO^ mice (Fig. 6b), concurrently with decreased serum ALT, hepatic lipid accumulation, fibrosis and MAS grading score (Fig. 6c–e). Consistently, hepatic mRNA levels of fibrogenic (*Tgfb1, Acta2*) and inflammatory (*Il6, Tnfa, F4/80*) genes, as well as protein levels of αSMA, COL1A2, TNFα, FASN, and ACC, were significantly reduced in the liver of SIRT3/cGAS^LDKO^ mice compared to those in SIRT3^LKO^ mice (Fig. 6f–h). To determine if the effect of cGAS on SIRT3 is cell-autonomous, we treated primary hepatocytes isolated from loxp, SIRT3^LKO^, or SIRT3/cGAS^LDKO^ mice with palmitic acid (PA) for 24 hrs. Knockout of cGAS greatly suppressed PA-induced lipid accumulation (Supplementary Fig. 6a), together with downregulation of lipid metabolism-related genes such as *Fasn, Scd1*, and *Acca* (Supplementary Fig. 6b), and inflammatory markers such as *Cxcl10* and *Il-6* (Supplementary Fig. 6c). Taken together, these results demonstrate that downregulation of the DsbA-L-cGAS axis mediates SIRT3 deficiency-induced inflammation, steatosis, and fibrosis.

## Discussion

In this study, we identified hepatic SIRT3 as a critical suppressor of MASH development in mice. The mechanism underlying the protective effect of SIRT3 is mediated by deacetylating the mitochondrial chaperon protein DsbA-L, which is critical for DsbA-L to enhance the capability of TFAM to promote mtDNA synthesis, thus alleviating mitochondrial stress-induced activation of the cGAS-STING signaling pathway and MASH pathogenesis. Our study not only reveals a hepatic-specific role of SIRT3 in metabolism but also uncovers a previously unrecognized functional link between SIRT3 and the cGAS-STING signaling pathway in MASH development in mice.

A previous multi-tissue proteomic study suggested that DsbA-L might be a potential target of SIRT3^12^. Here, we validate DsbA-L as a physiological substrate of SIRT3 and demonstrated that its deacetylation provides a mechanism by which SIRT3 regulates mitochondrial DNA biosynthesis and integrity. Both SIRT3^30^ and DsbA-L^31,32^ reside in the matrix of mitochondria and interact in hepatocytes. Notably, liver-specific knockout of DsbA-L exacerbated HFD-induced mitochondrial dysfunction, inflammation, hepatosteatosis, and insulin resistance in mice^33^, closely resembling the phenotypes observed in global SIRT3 knockout mice^14,34^. Mechanistically, SIRT3-mediated deacetylation enhanced DsbA-L binding to TFAM, promoting TFAM association with the mtDNA D-loop (Fig. 3d, e, h, i). In contrast, the acetylation-mimicking DsbA-L mutant weakened this interaction without affecting TFAM protein levels (Fig. 3g, j). Because TFAM–mtDNA binding is essential for nucleoid organization, mtDNA stability, and suppression of cGAS activation^27,28,35^, these findings identify DsbA-L acetylation as a critical regulatory switch. Although SIRT3 can directly deacetylate TFAM^36^, our results reveal an additional layer of regulation wherein SIRT3 indirectly modulates TFAM function through DsbA-L deacetylation, thereby preserving mitochondrial integrity and protecting against MASH-associated inflammation.

Interestingly, liver-specific knockout of DsbA-L^33^, but not SIRT3^17^, exacerbated HFD-induced metabolic abnormalities in mice. One possible explanation for this discrepancy is that SIRT3 targets numerous mitochondrial proteins, and its deacetylation effects can be either beneficial or detrimental depending on the functional context of each substrate. Thus, in hepatic SIRT3 knockout mice under HFD feeding, the detrimental effects of SIRT3 loss may be partially offset by beneficial effects resulting from increased acetylation of other mitochondrial proteins. Elucidating the specific roles of individual SIRT3 substrates, such as DsbA-L, will help clarify how SIRT3 fine-tunes metabolic homeostasis under different nutritional stresses.

The cGAS-STING pathway is the central cellular cytosolic double-stranded DNA sensor that plays a key role in immune defense in immune cells against pathogen infections^37^. In this study, liver-specific knockout of SIRT3 activated cGAS-STING signaling, whereas cGAS loss in wild-type or in SIRT3^LKO^ mice hepatocytes markedly alleviated MASH phenotypes in mice, establishing a causal role for this pathway in mitochondrial dysfunction-induced hepatic inflammation and fibrosis. Mitochondrial components such as mtDNA, ROS, and ATP act as danger-associated molecular patterns (DAMPs) that trigger inflammation^38^. In particular, mtDNA activates both cGAS-STING and TLR9 signaling, inducing NFκB and type I interferon responses, whereas the NLRP3 inflammasome is stimulated by mitochondrial-derived signals such as mtDNA, ATP, and ROS. Although SIRT3 deficiency may affect multiple inflammatory pathways, our data show increased cytosolic mtDNA and robust activation of cGAS-STING signaling, as indicated by elevated TBK1 and IRF3 phosphorylation, in SIRT3-deficient hepatocytes and livers (Fig. 2a–d). Genetic inhibition of cGAS or STING significantly reduced inflammatory gene expression, TBK1 and NFκB activation, and attenuated hepatic steatosis, injury, and fibrosis (Fig. 2e, 6b–h). While additional contributions from TLR9 or NLRP3 pathways cannot be excluded, especially given elevated mtDNA release and mtROS (Fig. 2d and Supplementary Fig. 5d), our results identify cGAS-STING as the predominant mediator of SIRT3 deficiency-induced hepatic inflammation. Future studies should further delineate the interplay among these mitochondrial stress-responsive pathways.

SIRT3 regulates glucose metabolism, mitochondrial homeostasis, and cellular stress responses^13,39,40^, yet its role in MASLD remains debated. Global SIRT3 deletion accelerates, while its overexpression mitigates, obesity, insulin resistance, steatosis, inflammation, hepatocyte death, and fibrosis compared with wild-type controls^15,16,34,41–43^. However, liver-specific studies have yielded mixed results. Fernández-Marcos et al.^17^ reported that SIRT3^LKO^ mice fed an HFD showed increased mitochondrial protein acetylation without major metabolic changes, and hepatic SIRT3 overexpression had a limited impact on substrate metabolism or HFD-induced defects^44^. Other studies found hepatic SIRT3 either alleviated steatosis in MTP + /– mice^45^ or MCD-induced MASH in AhR-transgenic mice^43^, or worsened SFA-induced liver injury by inhibiting AMPK and autophagy^46^. These discrepancies likely reflect differences in dietary stress. Under the milder metabolic stress, such as HFD, where steatosis occurs without severe inflammation or fibrosis^47–49^, SIRT3 deficiency may be compensated by alternative pathways. However, under harsher conditions, such as fructose-, sucrose-, cholesterol-, or choline-deficient diets, these compensations fail, and mitochondrial dysfunction, particularly involving the DsbA-L/TFAM axis and mtDNA homeostasis, may amplify cGAS activation and drive MASH progression. This context-dependent role of SIRT3 warrants further study.

A limitation of our study is the absence of the GAN diet in all models. Although choline deficiency may impair SIRT3 function^50,51^, our findings show that SIRT3 loss alone is sufficient to trigger MASH even under choline-sufficient GAN feeding (Fig. 1e–m). Moreover, cGAS activation in the livers of GAN-fed SIRT3^LKO^ mice (Fig. 2b) supports the conclusion that mitochondrial dysfunction-induced cGAS signaling, independent of choline status, is a key driver of MASH progression in SIRT3-deficient livers.

Although mtDNA stress and cGAS activation in hepatocytes contribute to the development of MASH in both SIRT3^LKO^ and DsbA-L^LKO^ mice, the precise connection between cGAS-STING signaling and hepatic steatosis remains elusive. Interestingly, STING has been shown to regulate lipid metabolism through mechanisms independent of its classical role in innate immunity. For example, STING fine-tunes the fatty acid desaturase 2 (FADS2)-dependent desaturation of polyunsaturated fatty acids (PUFAs), influencing lipid metabolism directly^52^, and STING deletion in *Drosophila* reduced lipid storage and downregulates lipogenic genes such as *Fasn* and *Acc*^53^. Consistent with these findings, we observed great protection when knocking out cGAS on liver lipid accumulation (Fig. 5e) and downregulated FASN and ACC expression in SIRT3^LKO^ mice (Fig. 6d, h). Although SIRT3 may also affect lipid oxidation via deacetylating other mitochondrial targets^34,54^, these results highlight cGAS activation as a major contributor to SIRT3 deficiency-induced steatosis under MASH conditions.

Both SIRT3^55–57^ and the cGAS-STING pathway^18,58–61^ are implicated in the regulation of autophagy, including lipophagy and mitophagy. SIRT3 is primarily known to promote autophagy through several pathways, including AMPK/mTORC1, FOXO1, Pink/Parkin or ERK/CREB signaling^,55–57,62,63^. However, Li et al.^46^ reported a negative role of SIRT3 in autophagy. On the other hand, while cGAS and STING are known to promote autophagy via mechanisms involving Beclin-1, ULK1, STING trafficking from Golgi to endoplasmic reticulum, as well as TBK1 activation^60,64^, interestingly, fatty acid-induced cGAS-STING signaling has been shown to activate mTORC1, which in turn inhibits autophagy and lipophagy in fatty liver^65^. Since autophagy and lipophagy are essential for triglyceride breakdown and lipid droplet degradation, it is plausible that SIRT3 regulates lipophagy and suppresses lipid accumulation through SIRT3-induced lipophagy while also suppressing cGAS-STING signaling. This dual regulation may underlie SIRT3’s hepatoprotective role in MASH, warranting further investigation into the interplay between SIRT3 and cGAS-STING in autophagy-mediated lipid metabolism.

In summary, our study identifies hepatic SIRT3 as a key protector against MASH. SIRT3-mediated deacetylation of DsbA-L enhances its interaction with TFAM, a central regulator of mtDNA maintenance and mitochondrial integrity. Loss of SIRT3 disrupts this axis, leading to mitochondrial stress, cGAS-STING activation, and consequent hepatic steatosis, inflammation, and fibrosis. The discovery of the SIRT3-DsbA-L-TFAM axis provides new insight into mitochondrial regulation in liver disease and highlights a promising therapeutic target for MASH and other mitochondrial-related disorders, including metabolic, neurodegenerative, and age-associated diseases.

## Methods

### Mouse studies

Mice were housed under a controlled environment (22  ±  1 °C, relative humidity of 50% ± 5, 12:12 light-dark cycle) with free access to food and water. C57BL/6JSIRT3 ^fl/fl^ mice (Jackson Lab Stock no.031201) were crossed with C57BL/6JAlb-Cre hemizygous mice (Jackson Lab Stock no.003574) to obtain SIRT3^LKO^ mice. Liver-specific DsbA-L knockout mice (DsbA-L^LKO^) were described previously^33^. C57BL/6JcGAS ^fl/fl^ mice (Constructed by Cyagen Biosciences Inc.) were crossed with Alb-Cre mice to obtain cGAS^LKO^ mice. cGAS^LKO^ mice and SIRT3 ^LKO^ mice were further crossed to obtain liver specific SIRT3/cGAS double knockout mice (SIRT3/cGAS^LDKO^). For the MASH model, 8- to 10-week-old male mice were fed with either MCD diet (A02082002BR, Research Diets Inc.) or CDAHFD diet (A06071302, Research Diets Inc.) for 8 weeks, or GAN diet (D09100310, Research Diets Inc.) for 28 weeks. For AAV-mediated overexpression experiment, a total of 3 ×1011 vg/mouse of AAV virus expressing GFP or different mutant DsbA-L were injected via tail vein into the 8-week-old male SIRT3^LKO^ mice or into the control loxp mice, the mice were then fed with CDAHFD diet for 12 weeks. All animal experiments abide by ARRIVE guidelines. Mice were euthanized by (carbon dioxide) CO₂ inhalation, with death confirmed by cervical dislocation. This study was designed as a mechanistic investigation of MASH, and male mice were used to minimize sex hormone-related variability and ensure robust and reproducible disease phenotypes. All animal experiments were carried out following the animal license approved by the Animal Care and Use Committee of the University of Texas Health San Antonio (UTHSA, 20180005AR) and Central South University (CSU2021193 and CSU20240204).

### Primary mouse hepatocytes isolation and cell culture

Primary hepatocytes were isolated from male mice by a modified two-step collagenase procedure as described in our previous study^66^. Briefly, the liver was perfused sequentially with Hank’s Balanced Salt Solution (HBSS) and type IV collagenase digestion medium. Following digestion, the liver was excised, minced, and passed through a 100 μm cell strainer. The cell suspension was collected and washed three times by centrifugation at 50 × *g* for 5 min at 4 °C. Hepatocyte viability, assessed by trypan blue exclusion staining, exceeded 90%. Isolated primary hepatocytes were seeded in MEM Alpha medium supplemented with 0.1 μM dexamethasone, 10% fetal bovine serum (FBS), 100 IU/mL penicillin, and 100 μg/mL streptomycin.

### Cellular fractionation and quantification of cytosolic mtDNA

Total and the cytosolic DNA were isolated from cells according to the procedures as described in our previous studies^10,20^. The obtained DNAs were used for real-time quantitative PCR, and the cycle threshold (CT) value obtained from the whole-cell total DNA was used as a normalization control for the mtDNA value obtained from the cytosolic fractions.

### RT-qPCR and RNA-Sequencing analysis

Total RNA was isolated from cells using TRIzol reagent according to the manufacturer’s suggested protocol. Quantitative PCR reactions were performed using SYBR Green (Applied Biosystems) and quantitated using an Applied Biosystems 7900 HT sequence detection system. Duplicate runs of each sample were normalized to β-actin to determine the relative expression levels. A complete list of primers and sequences is in Supplementary Table S1. For RNA-Sequencing analysis, primary hepatocytes were isolated from LOXP and STING liver-specific knockout mice and were treated with PA (0.1 mM) for 24 h. Cells total RNA were then harvested using TRIzol. RNA-Seq libraries were prepared using VAHTS Universal V6 RNA-seq Library Prep Kit, following the manufacturer’s instructions. Library construction was performed by HuNan Biotechnology Corporation (Nuo Jiu Genomics Co., Ltd., Hunan, China). Then libraries were sequenced on an Illumina NovaSeq 6000 platform, and 150 bp paired-end reads were generated. The clean reads were mapped to the reference genome using HISAT2. Differential expression analysis for mRNA was performed using the R package edgeR. Differentially expressed RNAs with |log2(FC)| value > 1, *q*-value < 0.05 and one group’s mean FPKM > 1. Enrichment analysis was conducted by RStudio.

### Co-IP and Western blot

Co-immunoprecipitation and Western Blot analysis were performed according to the procedures as described in our previous studies^10,33^. Cells or tissues were lysed in ice-cold lysis buffer [150 mM NaCl, 1% Triton X-100, 50 mM HEPES (pH 7.6), 20 mM pyrophosphate, 20 mM β-glycerophosphate, 10 mM NaF] supplemented with 1 mM phenylmethylsulfonyl fluoride (PMSF) and protease inhibitor cocktail (Roche) for 20 min at 4 °C, followed by centrifugation at 14,000 × *g* for 10 min at 4 °C. Protein concentrations were determined using a BCA Protein Assay Kit (Thermo Fisher Scientific) following the manufacturer’s instructions. For co-immunoprecipitation (co-IP), 500 μg of total protein from cell/tissue lysates was incubated with 2 μg of target-specific primary antibodies or IgG control with constant rotation overnight at 4 °C. Subsequently, 60 μL of 50% Protein A/G agarose beads (GE Healthcare Life Sciences) were added, and the mixture was incubated for a further 2 hours at 4 °C with rotation. Beads were collected by centrifugation at 500 × *g* for 3 min at 4 °C and washed five times with ice-cold lysis buffer. Precipitated proteins were eluted by resuspending the beads in 2 × SDS loading buffer, followed by boiling at 95 °C for 10 min. For Western blot analysis, immunoprecipitated eluates or input cell/tissue lysates were separated by 10% SDS-PAGE and transferred onto 0.45 μm PVDF membranes (Millipore). The membranes were blocked with 5% non-fat milk or BSA in TBST for 1 h at room temperature and incubated with the primary antibodies overnight at 4 °C, followed by incubation with a secondary antibody for 1 h at room temperature. Immunoreactive bands were visualized using the Molecular Imager Chemi Doc XRS+ System (Bio-Rad), and densitometry analysis was performed using Image Lab software (version 5.1, Bio-Rad Laboratories). Primary Antibodies and dilution used in Western Blot: cGAS, CST, 31659 s (1:1000); STING, CST, 13647 (1:1000); p-TBK1, CST, 5483 (1:1000); TBK1, CST, 3013 (1:1000); p-IRF3, CST, 4947 (1:1000); IRF3, CST, 4302 (1:1000); TNFα, Abcam, 6671 (1:1000); TGFβ, Santa Cruz, 52893 (1:1000); αSMA, Santa Cruz, 32251 (1:1000); FASN, CST, 3180 (1:1000); ACC, CST, 3662 s (1:1000); COL1A2, Santa Cruz, 393573 (1:1000); FN, Santa Cruz, 8422 (1:1000); p-P65, CST, 3033 (1:1000); P65, CST, 4767 (1:1000); Sirt3, CST, 5490 (1:1000); DsbA-L, homemade (1:1000); TFAM, Abcam, 138351 (1:1000); Acetylated-Lysine, CST, 9441 s (1:1000); HA, CST, 3724 (1:1000); Myc, Homemade (1:1000); GAPDH, CST, 5174 (1:1000); Tubulin, Homemade (1:1000); Actin, Sigma, A3854 (1:1000). Primary antibodies used for Co-IP: Sirt3, CST, 5490 (IP, 1:200); DsbA-L, homemade (IP: 1:200); HA, CST, 3724 (IP, 1:200); Myc, Homemade (IP, 1:200). Secondary Antibodies and dilution used in Western Blot: Anti-Rabbit IgG HRP-linked, CST, 7074P2 (WB, 1:5000); Anti-Mouse IgG HRP-linked, CST, 7076P2 (WB, 1:5000); Mouse monoclonal [2A9] Anti-Rabbit IgG heavy chain (HRP), Abcam, 99702 (WB, 1:5000). Detailed information of all antibodies used is also provided in Reporting Supplementary. Uncropped and unprocessed blot images are available in the Source Data file.

### Immunofluorescence and 8-OH-dG staining

Cells were fixed with 4% paraformaldehyde at room temperature for 10 min. After being washed with PBS buffer twice, cells were permeabilized with 0.1% Triton X-100 and then washed with PBS for three times. Cells were subsequently blocked with 2% bovine serum albumin at room temperature for 1 h, then incubated with the primary antibodies at 4 °C overnight. Cells were then washed with PBS and incubated with a secondary antibody at room temperature for 1 h. Cells were then washed with PBS and incubated with DAPI at room temperature for 2 min. After washing with PBS, images were obtained by using a fluorescence microscope or a laser scanning confocal microscope. Primary antibodies used in IF: Sirt3, Santa Cruz, sc-365175 (IF, 1:100); DsbA-L, homemade (IF, 1:100); Anti-8-OHdG, Bioss, bs-1278R (IF, 1:100). Secondary antibodies used in IF: Goat anti-Rabbit IgG (H + L) Highly Cross-Adsorbed Secondary Antibody, Alexa Fluor 488, Invitrogen, A-11034 (IF, 1:200); Goat anti-Mouse IgG (H + L) Highly Cross-Adsorbed Secondary Antibody, Alex Fluor 568, Invitrogen, A-11031 (IF, 1:200). Detailed information of all antibodies used is also provided in Reporting Supplementary.

### Tunel staining

Assessment of cell death in frozen liver tissue slices was done using TUNEL staining (11684817910, Roche) following the manufacturer’s instructions. The sections were fixed with 4% paraformaldehyde for 20 min, then incubated with proteinase K for 30 min, followed by incubation with TUNEL mixed reaction solution for 2 hat 37 °C. The sections were then washed with PBS and incubated with DAPI at room temperature for 2 min. After washing with PBS, images were obtained by using a fluorescence microscope.

### Histological and MAS-score analysis

Liver tissues were fixed in 4% paraformaldehyde, embedded in paraffin and sectioned (4 μm thickness). The sections were subjected to hematoxylin-eosin staining (H&E) staining and Sirius Red using staining standard procedures. Images were acquired by Image Scope (Leica Biosystems Imaging, Inc., USA). Liver pathology was evaluated by pathologists according to the MASLD activity scoring system (MAS)^67^. The MAS criteria were applied to assess the severity of MASH, focusing on three key aspects of liver histology: steatosis, hepatocyte ballooning, and inflammation.

### Oil red O staining

Liver tissues were fixed in liquid nitrogen, embedded in ornithine carbamoyl transferase (OCT) medium and sectioned at − 20  °C at a thickness of 8 μm. Frozen liver sections were stained with Oil Red O (ORO; Sigma O-0625, Sigma-Aldrich) for quantifying lipid droplets in hepatocytes.

### Plasmids, mutagenesis, RNAi generation

Site-directed mutagenesis was performed by using the Quick-Change site-directed mutagenesis kit (Agilent Technologies) according to the Manufacturer’s instructions (A complete list of primers and sequences are shown in Supplementary Table S1). The PCR was performed in 40 cycles using 55 °C (30 sec) for annealing, 72 °C (6 min) for elongation, and 95 °C (30 sec) for denaturation. The HepIR (insulin-resistant mouse hepatocyte) cell line was a generous gift from Dr. Domenico Accili at the National Institutes of Health (NIH, Bethesda, MD, USA). The DsbA-L knockdown HepIR stable cell line was generated by cloning shRNA targeting DsbA-L into the pSIREN-RetroQ vector (BD Knockout RNAi System; BD Biosciences). The target sequences were as follows: DsbA-L, 5’-GCATGGAGCAACCAGAGAT-3’; Scramble control, 5’-GCCGTAGCACTGGAAGAAA-3’. HepIR cells were transfected with the respective DsbA-L shRNA or scrambled control plasmids, followed by selection with 3 μg/ml puromycin. Individual stable clones were isolated by limited dilution and validated for efficient DsbA-L knockdown by western blot analysis, as indicated in our previous study^33^.

### Mitochondrial isolation

Trypsinized cells (2 × 10^6^) were centrifuged at 300 × *g* for 3 min at 4 °C, the pellet was washed twice with ice-cold PBS buffer. Cells were lysed with 1 mL ice-cold hypotonic buffer (3.5 mM Tris-HCl, pH 7.8, 2.5 mM NaCl, 0.5 mM MgCl_2_) and homogenized with a homogenizer for 10 times on ice. The lysates were suspended in 100 μL hypertonic buffer (350 mM Tris-HCl, pH 7.8, 250 mM NaCl, 50 mM MgCl_2_) and centrifuged at 1200 x *g* for 3 min at 4 °C. The supernatant was continually centrifuged at 1200 x *g* for 3 min and 15,000 x *g* for 2 min. The pellet was then washed with ice-cold buffer A (10 mM Tris-HCl, pH 7.4, 1 mM EDTA, 320 mM sucrose) three times. After centrifugation at 15,000 x *g* for 2 min, the mitochondrial pellet was resuspended in ice-cold buffer for subsequent experiments.

### ATP measurement

ATP levels in aliquots of freshly isolated mitochondria were measured by a chemiluminescent assay with an ATP determination kit (Thermo Fisher Scientific) following the manufacturer’s protocol. Briefly, mitochondria were incubated with the reaction buffer containing luciferin-luciferase ATP-monitoring reagent in an opaque 96-well plate, and the luciferase signals were detected at 560 nm using the Synergy HT Multi-Detection Microplate Reader (BioTek Instruments).

### TFAM and mtDNA binding assay

Cells were lysed with ice-cold lysis buffer (150 mM NaCl, 1% Triton X-100, 50 mM Tris-HCl, pH 8.0) containing protease and phosphatase inhibitors. The supernatant was collected and incubated with a biotinylated-LSP-DNA probe at 4 °C for 1 h. The biotinylated-LSP-DNA was pulled down by incubation with the streptavidin-conjugated agarose beads at 4 °C for 1 h. After centrifugation at 3300 x *g* for 1 min, the pellet was washed with lysis buffer and then resolved by SDS-PAGE. The LSP-DNA bound TFAM was determined by Western blot using an anti-TFAM antibody. The relative protein abundance was analyzed by Image Lab software. The relative TFAM binding was calculated as the ratio of the amount of TFAM protein pulled down to the total TFAM protein levels in the input.

### Oxygen consumption analysis

Cells were plated in a XF 24-well microplate (Seahorse Bioscience), followed by OCR measurement at 37 °C using an XF 24 Analyzer (Seahorse Bioscience) in accordance with the manufacturer’s instructions. Then, 0.5 μM oligomycin, 2 μM FCCP, 1 μM rotenone and antimycin were used to measure OCR at various states of mitochondrial respiration.

### Measurement of mitochondrial membrane potential and mtROS

Cells were incubated with the dye working solution (free-serum culture medium containing 200 nM TMRM or 5 μM MitoSOX^TM^ regent and 10 ng/mL Hoechst-33342) in a 37 °C, 5% CO_2_ incubator for 20 min. Cells were collected by centrifugation at 300 x *g* for 5 min and then washed three times with PBS. Cells were resuspended in PBS, and equal numbers of the cells were aliquoted to an opaque 96-well plate. The fluorescence signals were detected at Ex540/Em575 nm (for TMRM), Ex510/Em580 nm (for MitoSOX^TM^), and Ex350/Em461 nm (for Hoechst-33342) using the Synergy HT Multi-Detection Microplate Reader (BioTek Instruments).

### Statistics and reproducibility

All statistical analyses were performed using Prism software (GraphPad Prism 10). Significance was assessed using the unpaired, two-sided *t* test, One-way ANOVA, or Two-way ANOVA, as indicated in individual figures. Statistical significance was set at * *P* < 0.05, ** *P* < 0.01, *** *P* < 0.001, **** *P* < 0.0001. Quantitative data were presented as mean ± SEM. All experiments are independently repeated for 2-3 times.

### Reporting summary

Further information on research design is available in the Nature Portfolio Reporting Summary linked to this article.

## Supplementary information

Supplementary Information

Reporting Summary

Transparent Peer Review file

## Source data

Source Data

## Data Availability

Data supporting the findings of this study are available within the article and its supplementary information. The RNA-seq data have been deposited in the Gene Expression Omnibus (GEO) under accession number GSE281667. Source data are provided in the Supplementary Information/Source Data file. Source data are provided in this paper.

## References

[1] Sheka, A. C. et al. Nonalcoholic steatohepatitis: a review. *JAMA* **323**, 1175–1183 (2020). 32207804 10.1001/jama.2020.2298

[2] Grattagliano, I. et al. Targeting mitochondria to oppose the progression of nonalcoholic fatty liver disease. *Biochem. Pharm.* **160**, 34–45 (2019). 30508523 10.1016/j.bcp.2018.11.020

[3] Wallace, D. C. A mitochondrial bioenergetic etiology of disease. *J. Clin. Invest.* **123**, 1405–1412 (2013). 23543062 10.1172/JCI61398 PMC3614529

[4] Barshad, G., Marom, S., Cohen, T. & Mishmar, D. Mitochondrial DNA transcription and its regulation: an evolutionary perspective. *Trends Genet*.**34**, 682–692 (2018). 29945721 10.1016/j.tig.2018.05.009

[5] Picca, A. & Lezza, A. M. Regulation of mitochondrial biogenesis through TFAM-mitochondrial DNA interactions: Useful insights from aging and calorie restriction studies. *Mitochondrion* **25**, 67–75 (2015). 26437364 10.1016/j.mito.2015.10.001

[6] West, A. P. et al. Mitochondrial DNA stress primes the antiviral innate immune response. *Nature* **520**, 553–557 (2015). 25642965 10.1038/nature14156 PMC4409480

[7] Li, T. & Chen, Z. J. The cGAS-cGAMP-STING pathway connects DNA damage to inflammation, senescence, and cancer. *J. Exp. Med.* **215**, 1287–1299 (2018). 29622565 10.1084/jem.20180139 PMC5940270

[8] Yu, Y. et al. STING-mediated inflammation in Kupffer cells contributes to progression of nonalcoholic steatohepatitis. *J. Clin. Invest.* **129**, 546–555 (2019). 30561388 10.1172/JCI121842 PMC6355218

[9] Luo, X. et al. Expression of STING Is Increased in Liver Tissues From Patients With NAFLD and Promotes Macrophage-Mediated Hepatic Inflammation and Fibrosis in Mice. *Gastroenterology* **155**, 1971–1984 (2018). 30213555 10.1053/j.gastro.2018.09.010 PMC6279491

[10] Bai, J. et al. DsbA-L prevents obesity-induced inflammation and insulin resistance by suppressing the mtDNA release-activated cGAS-cGAMP-STING pathway. *Proc. Natl. Acad. Sci. USA* **114**, 12196–12201 (2017). 29087318 10.1073/pnas.1708744114 PMC5699051

[11] Hebert, A. S. et al. Calorie restriction and SIRT3 trigger global reprogramming of the mitochondrial protein acetylome. *Mol. Cell* **49**, 186–199 (2013). 23201123 10.1016/j.molcel.2012.10.024 PMC3704155

[12] Dittenhafer-Reed, K. E. et al. SIRT3 mediates multi-tissue coupling for metabolic fuel switching. *Cell Metab.* **21**, 637–646 (2015). 25863253 10.1016/j.cmet.2015.03.007 PMC4393847

[13] Guarente, L. Franklin H. Epstein Lecture: Sirtuins, aging, and medicine. *N. Engl. J. Med.* **364**, 2235–2244 (2011). 21651395 10.1056/NEJMra1100831

[14] Liu, L. et al. Nutrient sensing by the mitochondrial transcription machinery dictates oxidative phosphorylation. *J. Clin. Invest.* **124**, 768–784 (2014). 24430182 10.1172/JCI69413 PMC4381729

[15] Hirschey, M. D. et al. SIRT3 deficiency and mitochondrial protein hyperacetylation accelerate the development of the metabolic syndrome. *Mol. Cell* **44**, 177–190 (2011). 21856199 10.1016/j.molcel.2011.07.019 PMC3563434

[16] Chen, M. et al. SIRT3 Deficiency promotes high-fat diet-induced nonalcoholic fatty liver disease in correlation with impaired intestinal permeability through gut microbial dysbiosis. *Mol. Nutr. Food Res.* **63**, e1800612 (2019). 30525304 10.1002/mnfr.201800612

[17] Fernandez-Marcos, P. J. et al. Muscle or liver-specific Sirt3 deficiency induces hyperacetylation of mitochondrial proteins without affecting global metabolic homeostasis. *Sci. Rep.* **2**, 425 (2012). 22645641 10.1038/srep00425 PMC3361023

[18] Bai, J. & Liu, F. The cGAS-cGAMP-STING pathway: a molecular link between immunity and metabolism. *Diabetes* **68**, 1099–1108 (2019). 31109939 10.2337/dbi18-0052 PMC6610018

[19] Bause, A. S. & Haigis, M. C. SIRT3 regulation of mitochondrial oxidative stress. *Exp. Gerontol.* **48**, 634–639 (2013). 22964489 10.1016/j.exger.2012.08.007

[20] Bai, J. et al. Mitochondrial stress-activated cGAS-STING pathway inhibits thermogenic program and contributes to overnutrition-induced obesity in mice. *Commun. Biol.* **3**, 257 (2020). 32444826 10.1038/s42003-020-0986-1 PMC7244732

[21] Qiao, J. T. et al. Activation of the STING-IRF3 pathway promotes hepatocyte inflammation, apoptosis and induces metabolic disorders in nonalcoholic fatty liver disease. *Metabolism* **81**, 13–24 (2018). 29106945 10.1016/j.metabol.2017.09.010

[22] Donne, R. et al. Replication stress triggered by nucleotide pool imbalance drives DNA damage and cGAS-STING pathway activation in NAFLD. *Dev. Cell* **57**, 1728–1741 e1726 (2022). 35768000 10.1016/j.devcel.2022.06.003

[23] Ma, H., Kang, Z., Foo, T. K., Shen, Z. & Xia, B. Disrupted BRCA1-PALB2 interaction induces tumor immunosuppression and T-lymphocyte infiltration in HCC through cGAS-STING pathway. *Hepatology* **77**, 33–47 (2023). 35006619 10.1002/hep.32335 PMC9271123

[24] Schulze, S. et al. Cytosolic nucleic acid sensors of the innate immune system promote liver regeneration after partial hepatectomy. *Sci. Rep.* **8**, 12271 (2018). 30115978 10.1038/s41598-018-29924-3 PMC6095902

[25] Chen, R., Du, J., Zhu, H. & Ling, Q. The role of cGAS-STING signalling in liver diseases. *JHEP Rep.* **3**, 100324 (2021). 34381984 10.1016/j.jhepr.2021.100324 PMC8340306

[26] Rardin, M. J. et al. Label-free quantitative proteomics of the lysine acetylome in mitochondria identifies substrates of SIRT3 in metabolic pathways. *Proc. Natl. Acad. Sci. USA* **110**, 6601–6606 (2013). 23576753 10.1073/pnas.1302961110 PMC3631688

[27] Kaufman, B. A. et al. The mitochondrial transcription factor TFAM coordinates the assembly of multiple DNA molecules into nucleoid-like structures. *Mol. Biol. Cell* **18**, 3225–3236 (2007). 17581862 10.1091/mbc.E07-05-0404 PMC1951767

[28] Rebelo, A. P., Dillon, L. M. & Moraes, C. T. Mitochondrial DNA transcription regulation and nucleoid organization. *J. Inherit. Metab. Dis.* **34**, 941–951 (2011). 21541724 10.1007/s10545-011-9330-8

[29] Fisher, R. P. & Clayton, D. A. A transcription factor required for promoter recognition by human mitochondrial RNA polymerase. Accurate initiation at the heavy- and light-strand promoters dissected and reconstituted in vitro. *J. Biol. Chem.* **260**, 11330–11338 (1985). 4030791

[30] Kincaid, B. & Bossy-Wetzel, E. Forever young: SIRT3 a shield against mitochondrial meltdown, aging, and neurodegeneration. *Front. Aging Neurosci.* **5**, 48 (2013). 24046746 10.3389/fnagi.2013.00048 PMC3764375

[31] Thomson, R. E. et al. Tissue-specific expression and subcellular distribution of murine glutathione S-transferase class kappa. *J. Histochem. Cytochem.* **52**, 653–662 (2004). 15100242 10.1177/002215540405200509

[32] Harris, J. M., Meyer, D. J., Coles, B. & Ketterer, B. A novel glutathione transferase (13-13) isolated from the matrix of rat liver mitochondria having structural similarity to class theta enzymes. *Biochem. J.* **278**, 137–141 (1991). 1883325 10.1042/bj2780137 PMC1151459

[33] Chen, H. et al. Hepatic DsbA-L protects mice from diet-induced hepatosteatosis and insulin resistance. *FASEB J.* **31**, 2314–2326 (2017). 28232481 10.1096/fj.201600985R PMC5434661

[34] Hirschey, M. D. et al. SIRT3 regulates mitochondrial fatty-acid oxidation by reversible enzyme deacetylation. *Nature* **464**, 121–125 (2010). 20203611 10.1038/nature08778 PMC2841477

[35] Larsson, N. G. et al. Mitochondrial transcription factor A is necessary for mtDNA maintenance and embryogenesis in mice. *Nat. Genet.* **18**, 231–236 (1998). 9500544 10.1038/ng0398-231

[36] Liu, H., Li, S., Liu, X., Chen, Y. & Deng, H. SIRT3 overexpression inhibits growth of kidney tumor cells and enhances mitochondrial biogenesis. *J. Proteome Res.* **17**, 3143–3152 (2018). 30095923 10.1021/acs.jproteome.8b00260

[37] Ablasser, A. & Chen, Z. J. cGAS in action: Expanding roles in immunity and inflammation.* Science* **363**, eaat8657 (2019). 10.1126/science.aat8657 30846571

[38] Marchi, S., Guilbaud, E., Tait, S. W. G., Yamazaki, T. & Galluzzi, L. Mitochondrial control of inflammation. *Nat. Rev. Immunol.* **23**, 159–173 (2023). 35879417 10.1038/s41577-022-00760-x PMC9310369

[39] McDonnell, E., Peterson, B. S., Bomze, H. M. & Hirschey, M. D. SIRT3 regulates progression and development of diseases of aging. *Trends Endocrinol. Metab.* **26**, 486–492 (2015). 26138757 10.1016/j.tem.2015.06.001 PMC4558250

[40] Kitada, M., Ogura, Y., Monno, I. & Koya, D. Sirtuins and type 2 diabetes: role in inflammation, oxidative stress, and mitochondrial function. *Front. Endocrinol.* **10**, 187 (2019). 10.3389/fendo.2019.00187 PMC6445872 30972029

[41] Barroso, E. et al. SIRT3 deficiency exacerbates fatty liver by attenuating the HIF1alpha-LIPIN 1 pathway and increasing CD36 through Nrf2. *Cell Commun. Signal* **18**, 147 (2020). 32912335 10.1186/s12964-020-00640-8 PMC7488148

[42] Pinteric, M. et al. Chronic high fat diet intake impairs hepatic metabolic parameters in ovariectomized sirt3 KO mice.* Int. J. Mol. Sci.*** 22**, 4277 (2021). 10.3390/ijms22084277 PMC8074326 33924115

[43] He, J. et al. Activation of the aryl hydrocarbon receptor sensitizes mice to nonalcoholic steatohepatitis by deactivating mitochondrial sirtuin deacetylase Sirt3. *Mol. Cell Biol.* **33**, 2047–2055 (2013). 23508103 10.1128/MCB.01658-12 PMC3647969

[44] Osborne, B. et al. Liver-specific overexpression of SIRT3 enhances oxidative metabolism, but does not impact metabolic defects induced by high fat feeding in mice. *Biochem. Biophys. Res. Commun.* **607**, 131–137 (2022). 35367825 10.1016/j.bbrc.2022.03.088

[45] Nassir, F., Arndt, J. J., Johnson, S. A. & Ibdah, J. A. Regulation of mitochondrial trifunctional protein modulates nonalcoholic fatty liver disease in mice. *J. Lipid Res.* **59**, 967–973 (2018). 29581157 10.1194/jlr.M080952 PMC5983392

[46] Li, S. et al. Sirtuin 3 acts as a negative regulator of autophagy dictating hepatocyte susceptibility to lipotoxicity. *Hepatology* **66**, 936–952 (2017). 28437863 10.1002/hep.29229 PMC5570642

[47] Machado, M. V. et al. Mouse models of diet-induced nonalcoholic steatohepatitis reproduce the heterogeneity of the human disease. *PLoS ONE* **10**, e0127991 (2015). 26017539 10.1371/journal.pone.0127991 PMC4446215

[48] Matsumoto, M. et al. An improved mouse model that rapidly develops fibrosis in non-alcoholic steatohepatitis. *Int. J. Exp. Pathol.* **94**, 93–103 (2013). 23305254 10.1111/iep.12008 PMC3607137

[49] Clapper, J. R. et al. Diet-induced mouse model of fatty liver disease and nonalcoholic steatohepatitis reflecting clinical disease progression and methods of assessment. *Am. J. Physiol. Gastrointest. Liver Physiol.* **305**, G483–G495 (2013). 23886860 10.1152/ajpgi.00079.2013

[50] Song, Y. F. et al. Dietary choline alleviates high-fat diet-induced hepatic lipid dysregulation via UPRmt modulated by SIRT3-mediated mtHSP70 deacetylation. *Int. J. Mol. Sci.*** 23**, 4204 (2022). 10.3390/ijms23084204 PMC9025889 35457022

[51] Xu, M. et al. Choline ameliorates cardiac hypertrophy by regulating metabolic remodelling and UPRmt through SIRT3-AMPK pathway. *Cardiovasc. Res.* **115**, 530–545 (2019). 30165480 10.1093/cvr/cvy217

[52] Vila, I. K. et al. STING orchestrates the crosstalk between polyunsaturated fatty acid metabolism and inflammatory responses. *Cell Metab.* **34**, e67358 125–139 (2021). 34986331 10.1016/j.cmet.2021.12.007 PMC8733004

[53] Akhmetova, K., Balasov, M. & Chesnokov, I. Drosophila STING protein has a role in lipid metabolism.* Elife*** 10**, e67358 (2021). 10.7554/eLife.67358 PMC8443252 34467853

[54] Xu, G. et al. Mitochondrial ACSS1-K635 acetylation knock-in mice exhibit altered metabolism, cell senescence, and nonalcoholic fatty liver disease. *Sci. Adv.* **10**, eadj5942 (2024). 38758779 10.1126/sciadv.adj5942 PMC11100568

[55] Liu, J. et al. Honokiol attenuates lipotoxicity in hepatocytes via activating SIRT3-AMPK mediated lipophagy. *Chin. Med.* **16**, 115 (2021). 34758848 10.1186/s13020-021-00528-w PMC8579168

[56] Li, R. et al. Therapeutic effect of Sirtuin 3 on ameliorating nonalcoholic fatty liver disease: The role of the ERK-CREB pathway and Bnip3-mediated mitophagy. *Redox Biol.* **18**, 229–243 (2018). 30056271 10.1016/j.redox.2018.07.011 PMC6079484

[57] Zhang, T. et al. SIRT3 promotes lipophagy and chaperon-mediated autophagy to protect hepatocytes against lipotoxicity. *Cell Death Differ.* **27**, 329–344 (2020). 31160717 10.1038/s41418-019-0356-z PMC7206074

[58] Watson, R. O., Manzanillo, P. S. & Cox, J. S. Extracellular M. tuberculosis DNA targets bacteria for autophagy by activating the host DNA-sensing pathway. *Cell* **150**, 803–815 (2012). 22901810 10.1016/j.cell.2012.06.040 PMC3708656

[59] Liang, Q. et al. Autophagy side of MB21D1/cGAS DNA sensor. *Autophagy* **10**, 1146–1147 (2014). 24879161 10.4161/auto.28769 PMC4091176

[60] Gui, X. et al. Autophagy induction via STING trafficking is a primordial function of the cGAS pathway. *Nature* **567**, 262–266 (2019). 30842662 10.1038/s41586-019-1006-9 PMC9417302

[61] Sliter, D. A. et al. Parkin and PINK1 mitigate STING-induced inflammation. *Nature* **561**, 258–262 (2018). 30135585 10.1038/s41586-018-0448-9 PMC7362342

[62] Zhang, T., Liu, J., Tong, Q. & Lin, L. SIRT3 Acts as a positive autophagy regulator to promote lipid mobilization in adipocytes via Activating AMPK.* Int. J. Mol. Sci.*** 21**, 372 (2020). 10.3390/ijms21020372 PMC7013837 31936019

[63] Xi, S., Chen, W. & Ke, Y. Advances in SIRT3 involvement in regulating autophagy-related mechanisms. *Cell Div.* **19**, 20 (2024). 38867228 10.1186/s13008-024-00124-y PMC11170824

[64] Konno, H., Konno, K. & Barber, G. N. Cyclic dinucleotides trigger ULK1 (ATG1) phosphorylation of STING to prevent sustained innate immune signaling. *Cell* **155**, 688–698 (2013). 24119841 10.1016/j.cell.2013.09.049 PMC3881181

[65] Liu, K. et al. Lipotoxicity-induced STING1 activation stimulates MTORC1 and restricts hepatic lipophagy. *Autophagy* **18**, 860–876 (2022). 34382907 10.1080/15548627.2021.1961072 PMC9037528

[66] Luo, L. et al. De-silencing Grb10 contributes to acute ER stress-induced steatosis in mouse liver. *J. Mol. Endocrinol.* **60**, 285–297 (2018). 29555819 10.1530/JME-18-0018

[67] Kleiner, D. E. et al. Design and validation of a histological scoring system for nonalcoholic fatty liver disease. *Hepatology* **41**, 1313–1321 (2005). 15915461 10.1002/hep.20701

