# Supplementary material for: The SIRT3-DsbA-L-TFAM axis restrains cGAS-driven metabolic dysfunction-associated steatohepatitis in male mice
Source: Nat Commun. 2026 May 5;17:8421. doi: 10.1038/s41467-026-72395-8 (PMC13478530; doi:10.1038/s41467-026-72395-8)

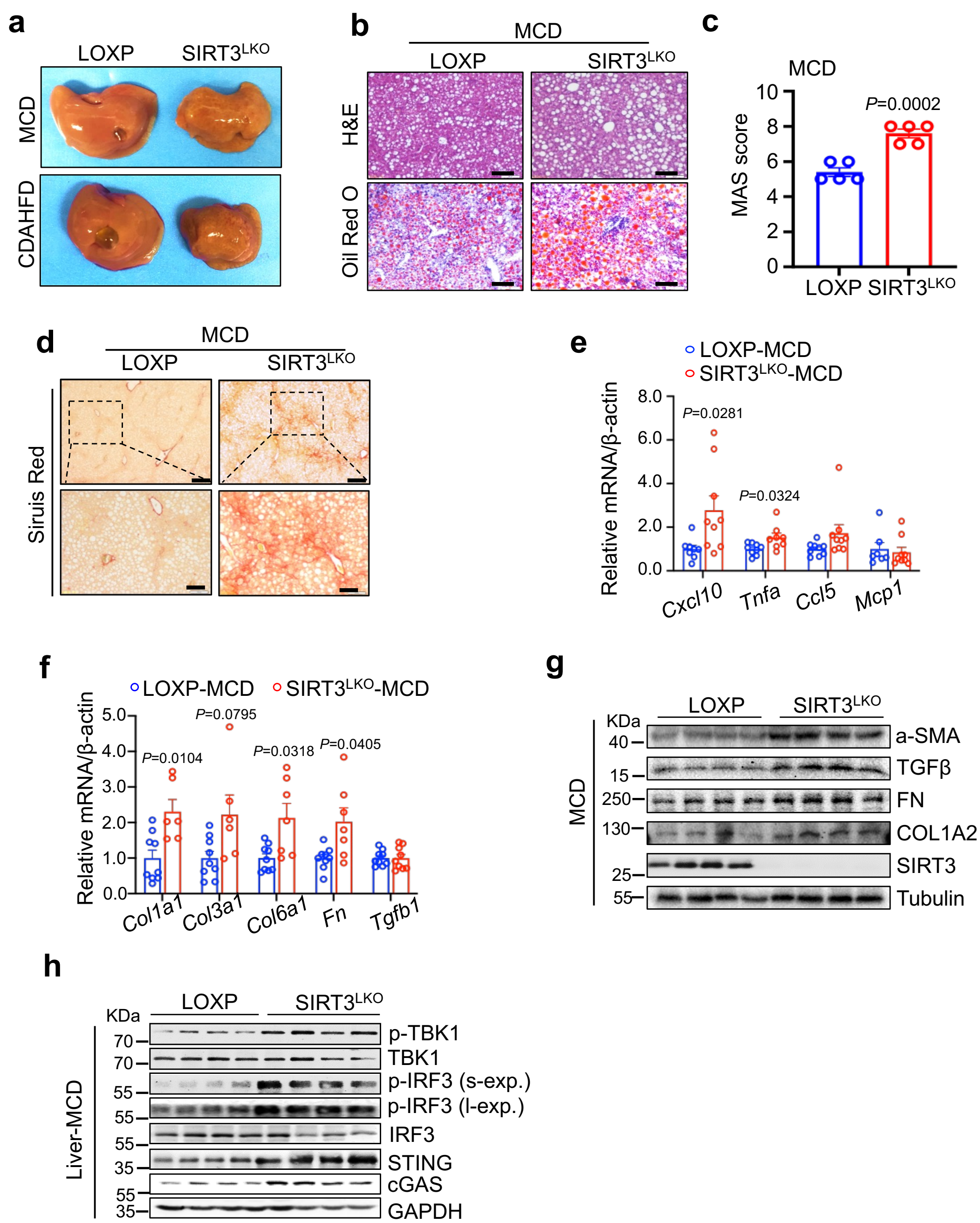

Supplementary Fig 1. Hepatic SIRT3 deficiency exacerbates MCD diet-induced MASH. a Livers of SIRT3^{LKO} and control wild-type mice fed an MCD or CDAHFD diet for 8 weeks. **b** H&E and Oil Red O staining of liver sections from MCD-fed SIRT3^{LKO} and control (loxp) mice (n=5/group). Scale bars, 100 µm. **c** MAS grading scores of MCD-fed SIRT3^{LKO} and control mice (n=5/group). **d** Sirius Red staining showing hepatic fibrosis in MCD-fed SIRT3^{LKO} and control mice (n=5/group). Scale bars, 50 µm for the upper layer, 100 µm for the lower layer. **e–g** Relative mRNA levels of inflammatory (**e**) and fibrotic (**f**)

genes, and protein levels of fibrosis markers (**g**) in livers of MCD-fed $SIRT3^{LKO}$ and control mice (n=9/group). **h** Enhanced cGAS signaling in livers of MCD-fed $SIRT3^{LKO}$ mice (n=4/group). Data are mean ± SEM; $P < 0.05$, $P < 0.01$ (unpaired two-sided *t-test* for panels c, e, f). Source data are provided as a Source Data file.

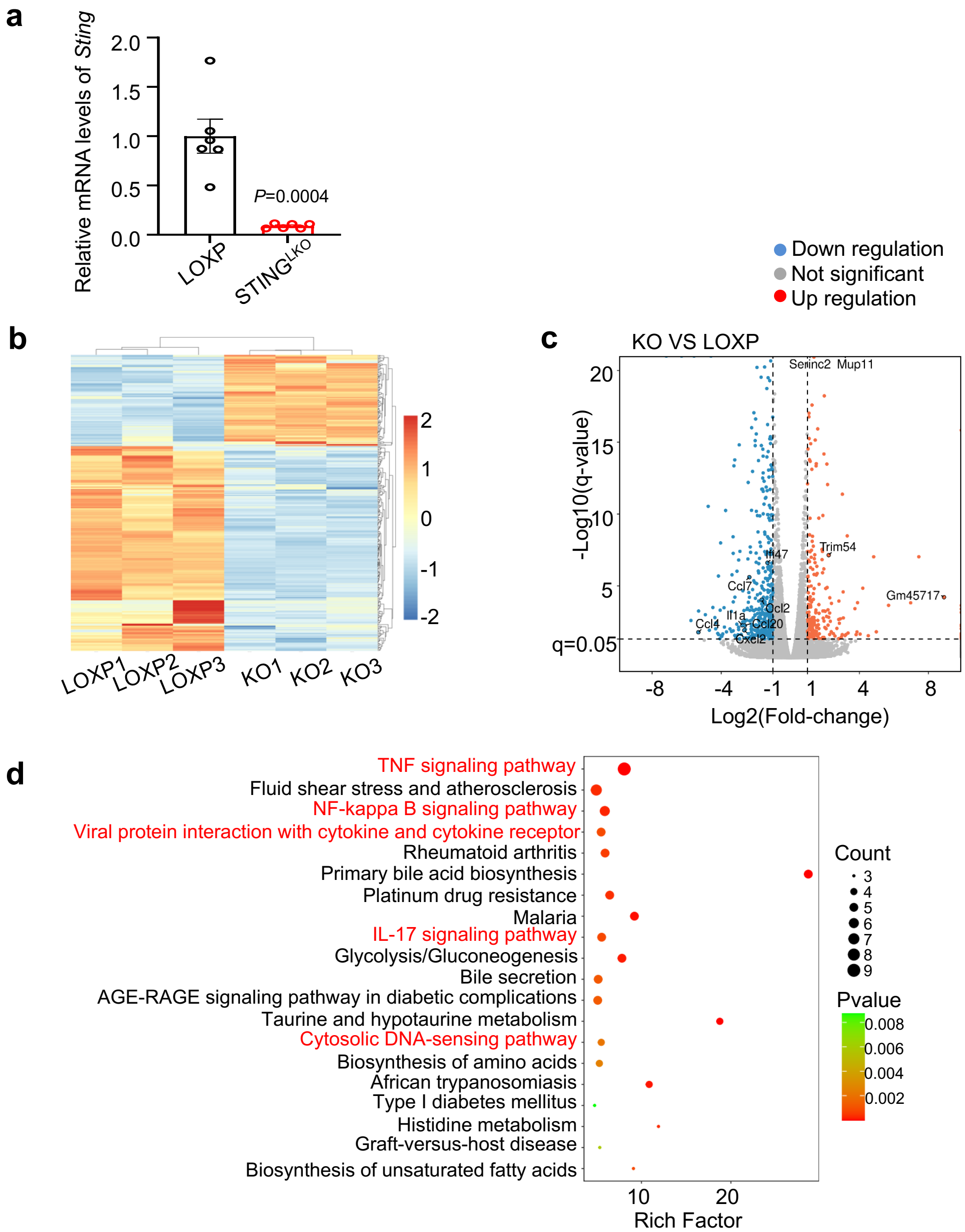

Supplementary Fig. 2. STING is expressed in hepatocytes and knockout of STING downregulates inflammatory genes. The hepatocytes-specific STING knockout (STING^{LKO}) mice were generated by crossing STING floxed mice with Albumin-Cre mice. Mouse hepatocytes were purified from both STING^{Loxp} and STING^{LKO} mice, followed by 0.1 mM palmitate treatment for 24 hrs. Cells were collected for RNA sequencing. **a** STING mRNA expression in purified hepatocytes from STING^{Loxp} and STING^{LKO} mice (n=6/group). data are presented as mean ± SEM. ** $P < 0.01$. **b** Heatmap showed 97 upregulated genes and 218 downregulated gene in STING-knockout hepatocytes compared to control group

(n=3/group). **c** Vocalo plot of DEGs in control and STING-knockout hepatocytes (n=3/group). **d** KEGG enrichment analysis made by downregulated DEGs showing pathways including TNF signaling, NF-kappa B signaling, viral protein related to cytokine and cytokine receptor, IL-17 signaling as well as cytosolic DNA sensing pathway were highly enriched. Statistical significance for (**a**) was determined by unpaired, two-sided *t*-test, and for (**b**, **c**, **d**) was determined by fold change > 2 and q value < 0.05. Source data are provided as a Source Data file.

a

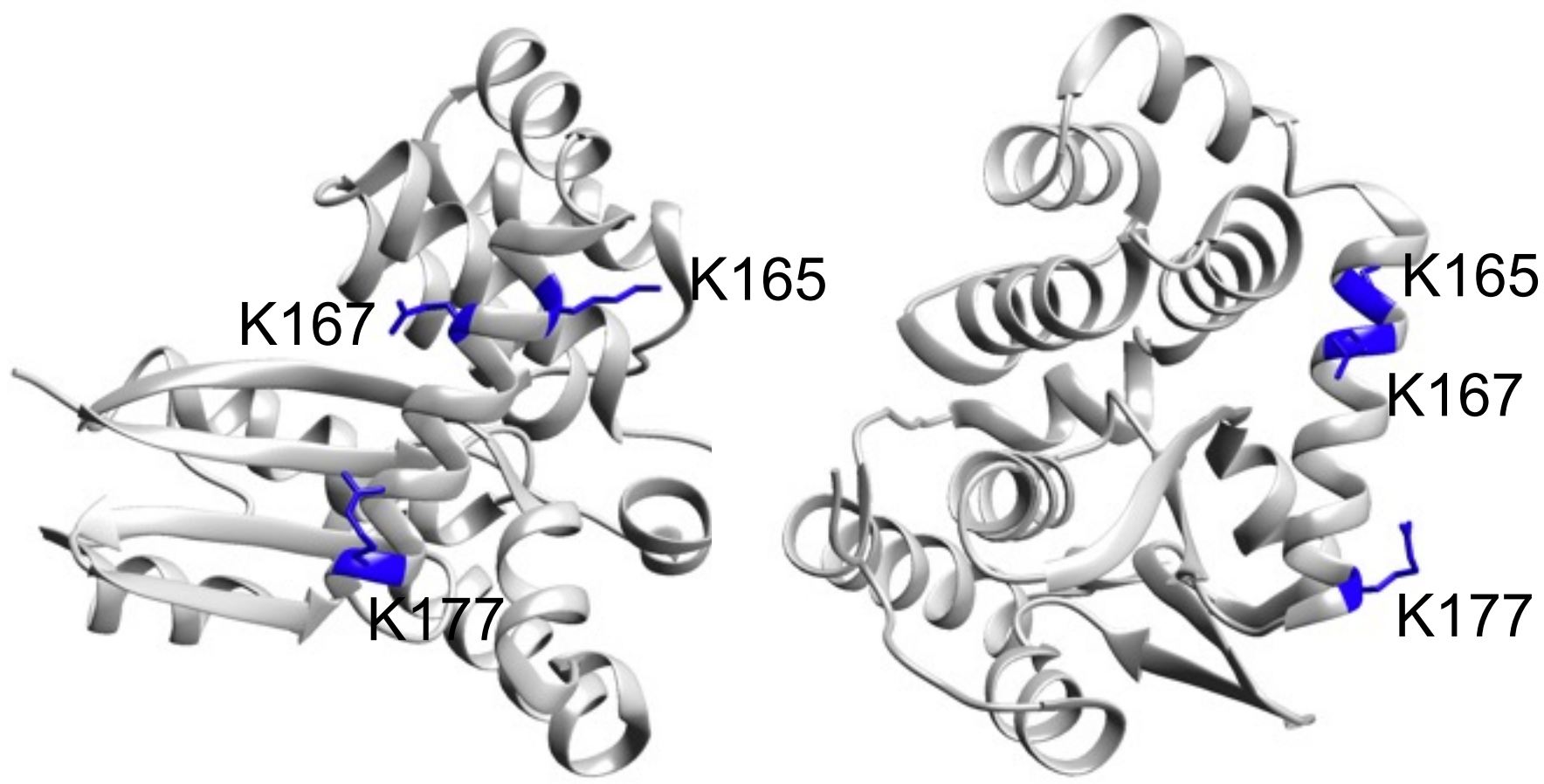

b

	165 167 177
Human	KI ATPKVKNQLKETTEAACRYGAFG
Chimpanzee	KI ATPKVKNQLKETTEAACRYGAFG
Horse	KISTPQVKNQLKETTEAACRYGAFG
Pig	RVSTPEVKNQLKETTEAACKYGAFG
Sheep	RVSTPQVKNQLKETTEAACKYGAFG
Dog	KI VTPKVKNKLKENTEAACKYGAFG
Rat	KI STELVKSKLRETTGAACKYGAFG
Mouse	KI STQQVKNKLI ENTDAACKYGAFG
Frog	STALPEVKNKLKQNTDEALKYGAFG
Zebrafish	LATSQPI KDKLKSVTSEA I KYKCFG

Supplementary Fig. 3. The position of putative acetylated lysine site in the structure of DsbA-L protein. a Cartoon representation of DsbA-L structure made by Pymol (www.pymol.org). Three putative lysine residues (i.e., K165, K167, and K177) were labeled in blue. **b** Sequence alignment of the putative acetylation sites of K165, K167, and K177 in DsbA-L from different species.

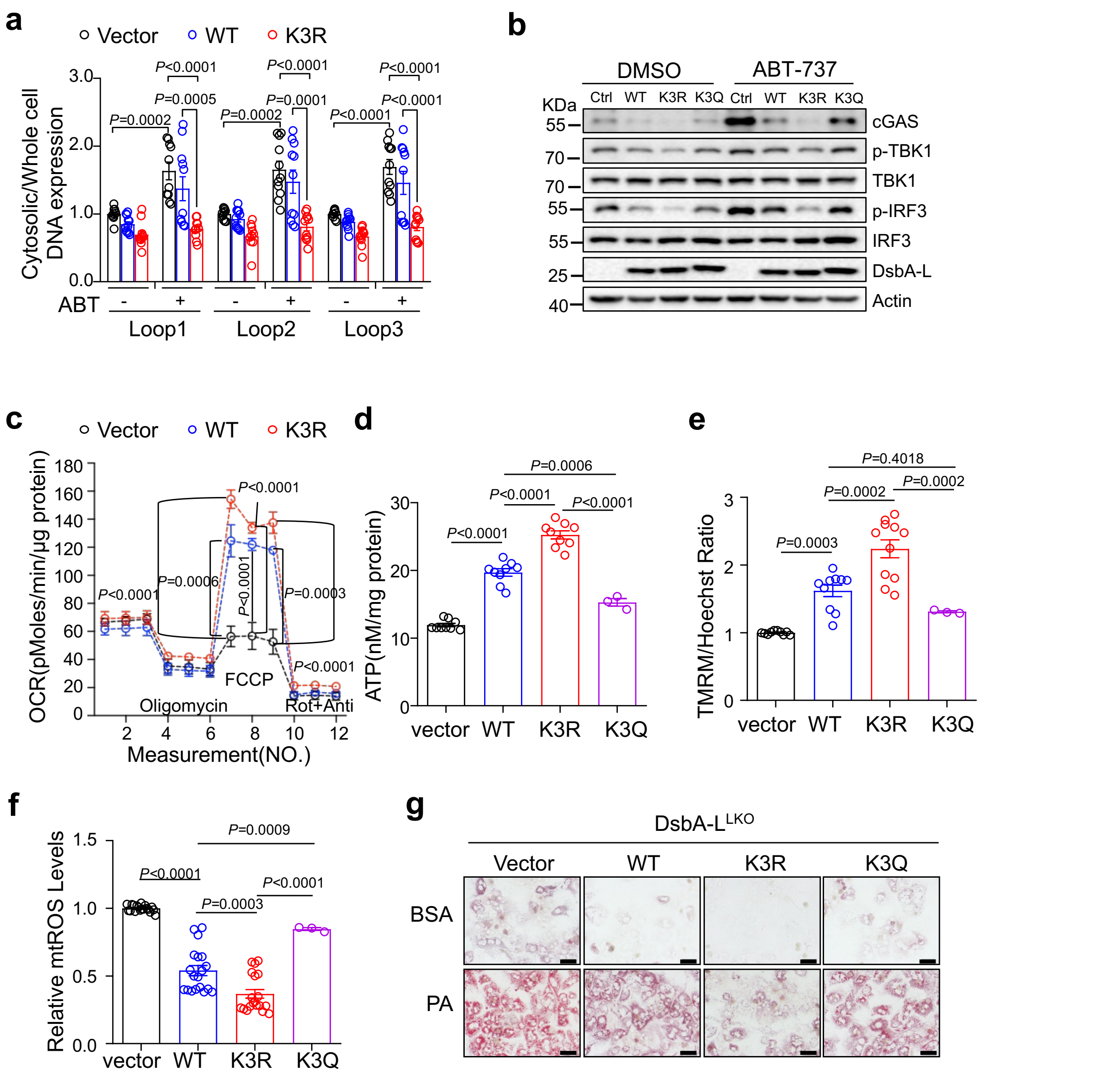

Supplementary Fig. 4. K3R but not K3Q mutant of DsbA-L alleviates mitochondrial dysfunction in DsbA-L deficient hepatocytes. a mtDNA release in DsbA-L knockout primary hepatocytes transiently overexpressing wild-type (WT) or K3R mutant of DsbA-L was determined by RT-qPCR (n=11/group). **b** Expression and/or phosphorylation of components in the cGAS pathway were detected by Western blot in DsbA-L knockout primary hepatocytes transiently overexpressing wild-type (WT), the K3R, or the K3Q mutant of DsbA-L (n=3 independent experiments). **c** Oxygen consumption rate was measured in DsbA-L knockout primary hepatocytes transiently overexpressing wild-type (WT) or the K3R mutant of DsbA-L (Vector, n=4; WT, n=4; K3R, n=6). ATP levels (**d**) (Vector, n=9; WT, n=9; K3R, n=9; K3Q, n=3), mitochondrial membrane potential (**e**) (Vector, n=10; WT, n=10; K3R, n=11; K3Q, n=3), and

mitochondrial ROS levels (**f**) (Vector, n=19; WT, n=19; K3R, n=19; K3Q, n=3) were measured in DsbA-L knockout primary hepatocytes transiently overexpressing wild-type (WT) or K3R or the K3Q mutant of DsbA-L. **g** Oil red O staining in DsbA-L knockout primary hepatocytes transiently overexpressing wild-type (WT), the K3R, or the K3Q mutant of DsbA-L treated with palmitic acid (PA, 0.2 mM) (n=3 independent experiments). Scale bars, 50 µm. In all statistical plots, data are presented as mean ± SEM. * $P < 0.05$, ** $P < 0.01$, *** $P < 0.001$, **** $P < 0.0001$. Statistical significance for (**a**, **c**, **d**, **e**, **f**) was determined by One-way ANOVA. Source data are provided as a Source Data file.

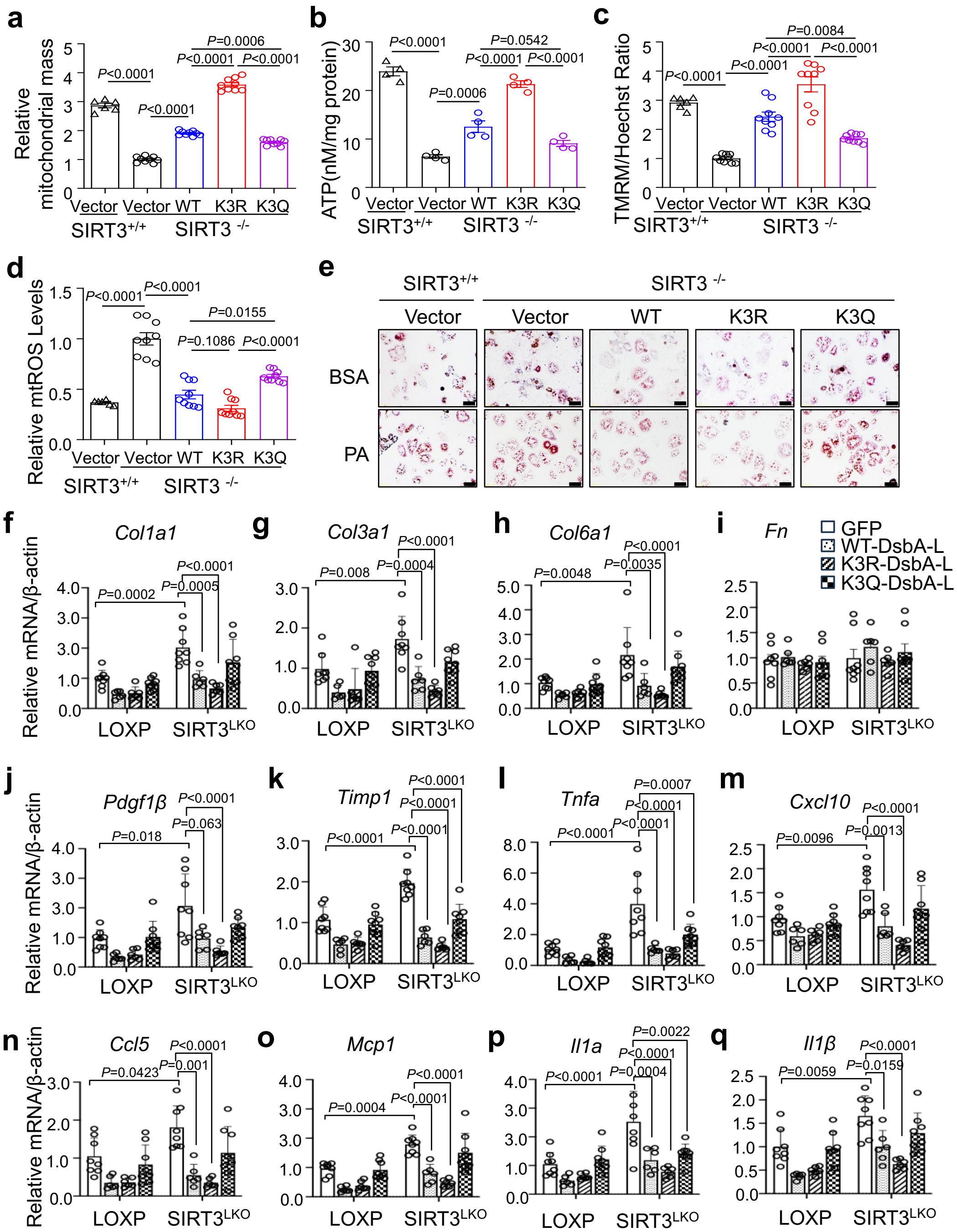

Supplementary Fig. 5. K3R but not K3Q mutant of DsbA-L alleviates SIRT3 deficiency-induced mitochondrial dysfunction and protects against CDAHFD-induced liver inflammation and fibrosis in SIRT3^{LKO} mice. Mitochondrial mass (**a**) (SIRT3$^{+/+}$ Vector, n=6; SIRT3$^{-/-}$ Vector, n=9; SIRT3$^{-/-}$ WT, n=9; SIRT3$^{-/-}$ K3R, n=9; SIRT3$^{-/-}$ K3Q, n=9), ATP levels (**b**) (n=4/group), mitochondrial membrane potential (**c**) (SIRT3$^{+/+}$ Vector, n=6; SIRT3$^{-/-}$ Vector, n=9; SIRT3$^{-/-}$ WT, n=9; SIRT3$^{-/-}$ K3R, n=9; SIRT3^{-}

$^{/-}$ K3Q, n=9), and mitochondrial ROS levels (**d**) ($SIRT3^{+/+}$ Vector, n=6; $SIRT3^{-/-}$ Vector, n=9; $SIRT3^{-/-}$ WT, n=9; $SIRT3^{-/-}$ K3R, n=9; $SIRT3^{-/-}$ K3Q, n=9) were measured in SIRT3 knockout primary hepatocytes transiently overexpressing wild-type (WT) or K3R or K3Q of DsbA-L. **e** Oil red O staining in SIRT3 knockout primary hepatocytes transiently overexpressing wild-type (WT) or K3R or K3Q of DsbA-L treated with PA (0.2 mM) (n= 3 independent experiments). Scale bars, 50 µm. **f-k** Relative mRNA levels of fibrosis-related genes in the livers of CDAHFD-fed $SIRT3^{LKO}$ and control loxp mice overexpressing different DsbA-L mutants (GFP, n=8; n=6; K3R, n=6; K3Q, n=8). **l-q** Relative mRNA levels of inflammatory genes in the livers of CDAHFD-fed $SIRT3^{LKO}$ and control loxp mice overexpressing different DsbA-L mutants (GFP, n=8; n=6; K3R, n=6; K3Q, n=8). In all statistical plots, data are presented as mean ± SEM, * $P < 0.05$, ** $P < 0.01$, *** $P < 0.001$, **** $P < 0.0001$. Statistical significance for (**a**, **b**, **c**, **d**) was determined by One-way ANOVA, and for (**f-q**) was determined by Two-way ANOVA. Source data are provided as a Source Data file.

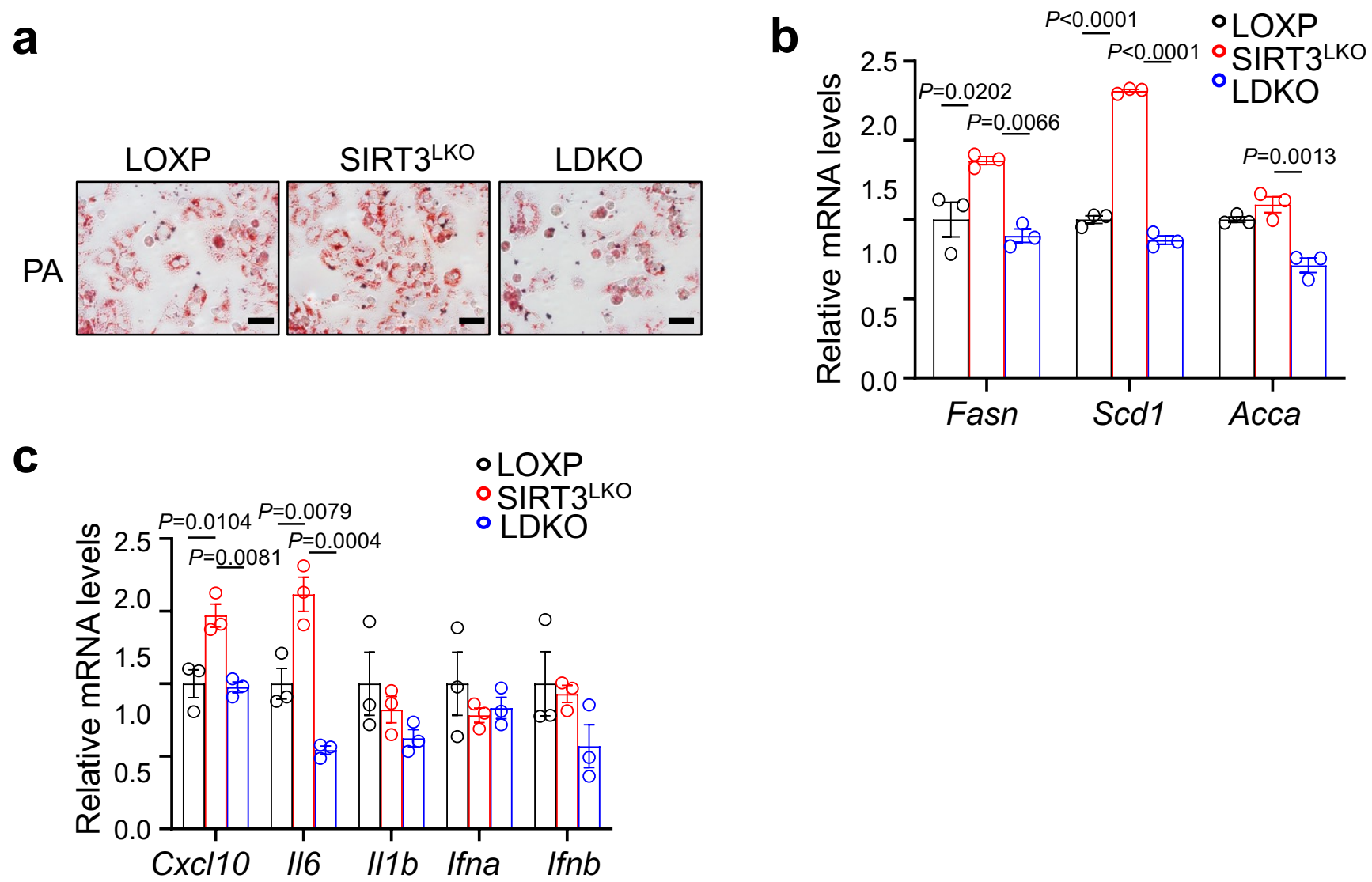

Supplementary Fig. 6. Ablation of cGAS diminished SIRT3 deficiency-induced lipid accumulation and inflammation in hepatocytes. a Oil red O staining in loxp, $SIRT3^{LKO}$ and $SIRT3/cGAS^{LDKO}$ hepatocytes treated with palmitic acid (PA, 0.2 mM). Scale bars, 50 μm (n= 3/group, from 2 independent experiments). **b** Lipogenesis and **c** inflammation related genes expression in loxp, $SIRT3^{LKO}$, and $SIRT3/cGAS^{LDKO}$ hepatocytes were measured by RT-qPCR (n= 3/group, from 2 independent experiments). In all statistical plots, data are presented as mean ± SEM. * $P < 0.05$, ** $P < 0.01$. Statistical significance for (**b**, **c**) was determined by One-way ANOVA. Source data are provided as a Source Data file.

Supplementary Table 1

Name	Primer Sequence	Application
Col1a1 F	ATGGATTCCCGTTCGAGTACG	RT-qPCR
Col1a1 R	CAGCTGGATAGCGACATCG	RT-qPCR
Col3a1 F	GACCAAAAGGTGATGCTGGACAG	RT-qPCR
Col3a1 R	CAAGACCTCGTGCTCCAGTTAG	RT-qPCR
Col6a1 F	GACACCTCTCAGTGTGCTCTGT	RT-qPCR
Col6a1 R	GCGATAAGCCTTGGCAGGAAATG	RT-qPCR
Fn F	AGACCATACCTGCCGAATGTAG	RT-qPCR
Fn R	GAGAGCTTCCTGTCCTGTAGAG	RT-qPCR
Tgfβ1 F	CCACCTGCAAGACCATCGAC	RT-qPCR
Tgfβ1 R	CTGGCGAGCCTTAGTTTGGAC	RT-qPCR
Timp1 F	TCTTGGTTCCCTGGCGTACTCT	RT-qPCR
Timp1 R	GTGAGTGTCACTCTCCAGTTTGC	RT-qPCR
Cxcl10 F	ATCATCCCTGCGAGCCTATCCT	RT-qPCR
Cxcl10 R	GACCTTTTTTGGCTAAACGCTTTC	RT-qPCR
Pdgf1β F	AATGCTGAGCGACCACTCCATC	RT-qPCR
Pdgf1β R	TCGGGTCATGTTCAAGTCCAGC	RT-qPCR
Tnfα F	GAGAAAGTCAACCTCCTCTCTG	RT-qPCR
Tnfα R	GAAGACTCCTCCCAGGTATATG	RT-qPCR
Ccl5 F	CCTGCTGCTTTGCCTACCTCTC	RT-qPCR
Ccl5 R	ACACACTTGGCGGTTCCTTCGA	RT-qPCR
Mcp1 F	CAGCCAGATGCAGTTAACGC	RT-qPCR
Mcp1 R	GCCTACTCATTGGGATCATCTTG	RT-qPCR
Il1α F	TGTAACAGACCTCAAGAA	RT-qPCR
Il1α R	CATCTAACTGACCACCTA	RT-qPCR
Il1β F	TGGACCTTCCAGGATGAGGACA	RT-qPCR
Il1β R	GTTCATCTCGGAGCCTGTAGTG	RT-qPCR
Il6 F	TTCCAATGCTCTCCTAACA	RT-qPCR
Il6 R	GTCCACAAACTGATATGCTTA	RT-qPCR
Acta2 F	AGAGCTACGAACTGCCTGAC	RT-qPCR
Acta2 R	CCTGTTTTGGCTCCCTATGTCT	RT-qPCR
F4/80 F	CTTTGGCTATGGGCTTCCAGTC	RT-qPCR
F4/80 R	GCAAGGAGGACAGAGTTTATCGTG	RT-qPCR
Col1a2 F	TTCTGTGGGTCCTGCTGGGAAA	RT-qPCR
Col1a2 R	TTGTCACCTCGGATGCCTTGAG	RT-qPCR
mtDNA Dloop1 F	AATCTACCATCCTCCGTGAAACC	RT-qPCR
mtDNA Dloop1 R	TCAGTTTAGCTACCCCCAAGTTTAA	RT-qPCR
mtDNA Dloop2 F	CCCTTCCCCATTTGGTCT	RT-qPCR
mtDNA Dloop2 R	TGGTTTCACGGAGGATGG	RT-qPCR
mtDNA Dloop3 F	TCCTCCGTGAAACCAACAA	RT-qPCR
mtDNA Dloop3 R	AGCGAGAAGAGGGGCATT	RT-qPCR
Cytb F	AAAGCCACCTTGACCCGATT	RT-qPCR
Cytb R	GATTCGTAGGGCCGCGATA	RT-qPCR
ND1 F	CTCAACCTAGCAGAAACAAACC	RT-qPCR
ND1 R	GGCCGGCTGCGTATTCTAC	RT-qPCR
ND4 F	AACGGATCCACAGCCGTA	RT-qPCR

Continued		
**Name**	**Primer Sequence**	**Application**
ND4 R	AGTCCTCGGGCCATGATT	RT-qPCR
ND6 F	TTGGGAGATTGGTTGATGTAT	RT-qPCR
ND6 R	TGCCGCTACCCCAATCC	RT-qPCR
CoxI F	TCAGTATCGTATGCTTCAACAAATTTAGA	RT-qPCR
CoxI R	TGGTTCCTCGAATGTGTGATATG	RT-qPCR
CoxII F	GAGCAGTCCCCTCCCTAGGA	RT-qPCR
CoxII R	GTCGGTTTGATGTTACTGTTGCTT	RT-qPCR
ATPase6 F	TCGTTGTAGCCATCATTATATTTCCT	RT-qPCR
ATPase6 R	GAAAGAATGGAGACGGTTGTTGA	RT-qPCR
Cox5α F	CTGCCCGGAGTCTGGTAATG	RT-qPCR
Cox5α R	CAGTCAACGTAGGGGGTCATC	RT-qPCR
TFAM F	ATTCCGAAGTGTTTTTCCAGCA	RT-qPCR
TFAM R	TCTGAAAGTTTTGCATCTGGGT	RT-qPCR
ERRα F	GACGGCAGAAGTACAAACGG	RT-qPCR
ERRα R	CAACCAGCAGATGCGACAC	RT-qPCR
Fasn F	GGAGGTGGTGATAGCCGGTAT	RT-qPCR
Fasn R	TGGGTAATCCATAGAGCCCAG	RT-qPCR
Scd1 F	CTGTACGGGATCATACTGGTTC	RT-qPCR
Scd1 R	GCCGTGCCTTGTAAGTTCTG	RT-qPCR
Acc1 F	TGCCACCACCTTATCACTATGTA	RT-qPCR
Acc1 R	CCTGCCTGTCTCCATCCA	RT-qPCR
Ifnα F	GGATGTGACCTTCCTCAGACTC	RT-qPCR
Ifnα R	ACCTTCTCCTGCGGGAATCAA	RT-qPCR
Ifnβ F	GCCTTTGCCATCCAAGAGATGC	RT-qPCR
Ifnβ R	ACACTGTCTGCTGGTGGAGTTC	RT-qPCR
DsbA-L F	GCAGTTGCGGCCCACTT	RT-qPCR
DsbA-L R	TGGTTGGTTTCCGCTGTCTT	RT-qPCR
β-Actin F	GAGACCTTCAACACCCCAGC	RT-qPCR
β-Actin R	ATGTCACGCACGATTTCCC	RT-qPCR
Gapdh F	GACTTCAACAGCAACTCCCAC	RT-qPCR
Gapdh R	TCCACCACCCTGTTGCTGTA	RT-qPCR
nDNA Tert F	CTAGCTCATGTGTCAAGACCCTCTT	RT-qPCR
nDNA Tert R	GCCAGCACGTTTCTCTCGTT	RT-qPCR
K49R-Top	CTTTAATCGCTGGGATCATGAGAGACAGCGGAAACC AACCAC	PCR
K49R-Bot	GTGGTTGGTTTCCGCTGTCTCTCATGATCCCAGCGATT AAAG	PCR
K62R-Top	CACCTGCTATGGTTCCCCGAAGAGGCCAGTACATCTT CAAAG	PCR
K62R-Bot	CTTTGAAGATGTACTGGCCTCTTCGGGGAACCATAGC AGGTG	PCR
K68R-Top	GAAAAGGCCAGTACATCTTCAGAGAGATTCCTCTCCT GAAG	PCR
K68R-Bot	CTTCAGGAGAGGAATCTCTCTGAAGATGTACTGGCCT TTTC	PCR

Continued		
**Name**	**Primer Sequence**	**Application**
K74R-Top	CAAAGAGATTCCTCTCCTGAGGCAGTTCTTCCAGGTTCCC	PCR
K74R-Bot	GGGAACCTGGAAGAACTGCCTCAGGAGAGGAATCTCTTTG	PCR
K85R-Top	GTTCCCCTCAACATACCCAGGGATTTCTTTGGTGAGAC	PCR
K85R-Bot	GTCTCACCAAAGAAATCCCTGGGTATGTTGAGGGGAAC	PCR
K93R-Top	GATTTCTTTGGTGAGACTGTGAGGAAAGGAAGTATAAATGCC	PCR
K93R-Bot	GGCATTTATACTTCCTTTCCTCACAGTCTCACCAAAGAAATC	PCR
K94R-Top	CTTTGGTGAGACTGTGAAGAGAGGAAGTATAAATGCCATG	PCR
K94R-Bot	CATGGCATTTATACTTCCTCTCTTCACAGTCTCACCAAAG	PCR
K116R-Top	CAACCAGAGATGCTGGAGAGGGTGTCCAGAGAGATATGG	PCR
K116R-Bot	CCATATCTCTCTGGACACCCTCTCCAGCATCTCTGGTTG	PCR
K144R-Top	CATTCTGGCTGCAGCAGTGAGGGCTGGAATGTCCACAGCGCAAG	PCR
K144R-Bot	CTTGCGCTGTGGACATTCCAGCCCTCACTGCTGCAGCCAGAATG	PCR
K158R-Top	CAAGCCCAACACTTTCTGGAGAGGATCTCCACACAACAGGTG	PCR
K158R-Bot	CACCTGTTGTGTGGAGATCCTCTCCAGAAAGTGTTGGGCTTG	PCR
K165R-Top	GATCTCCACACAACAGGTGAGGAACAAGCTCATTGAGAAC	PCR
K165R-Bot	GTTCTCAATGAGCTTGTTCCTCACCTGTTGTGTGGAGATC	PCR
K167R-Top	CACACAACAGGTGAAGAACAGGCTCATTGAGAACACGGATG	PCR
K167R-Bot	CATCCGTGTTCTCAATGAGCCTGTTCTTCACCTGTTGTGTG	PCR
K177R-Top	GAACACGGATGCAGCCTGCAGATATGGGGCCTTTGGGCTAC	PCR
K177R-Bot	GTAGCCCAAAGGCCCCATATCTGCAGGCTGCATCCGTGTTC	PCR
K165+167R-Top	CACACAACAGGTGAGGAACAGGCTCATTGAGAACACGGATG	PCR
K165+167R-Bot	CATCCGTGTTCTCAATGAGCCTGTTCCTCACCTGTTGTGTG	PCR
K213R-Top	GCTTACCTGCTAGGAGAGAGGTGGATGGGCCCTGTAC	PCR
K213R-Bot	GTACAGGGCCCATCCACCTCTCTCCTAGCAGGTAAGC	PCR

Continued		
**Name**	**Primer Sequence**	**Application**
K177Q-Top	GAACACGGATGCAGCCTGCCAATATGGGGCCTTTGG GCTAC	PCR
K177Q-Bot	GTAGCCCAAAGGCCCCATATTGGCAGGCTGCATCCGT GTTC	PCR
K165+167Q-Top	CACACAACAGGTGCAGAACCAGCTCATTGAGAACAC GGATG	PCR
K165+167Q-Bot	CATCCGTGTTCTCAATGAGCTGGTTCTGCACCTGTTGT GTG	PCR
Biotin-LSP-F	BIOTIN-TGTGTTAGTTGGGGGGTGACTGTTAAA	IP
Biotin-LSP-R	TTTAACAGTCACCCCCCAACTAACACA	IP
Biotin-ND1-F	BIOTIN- ACTACAACCCTTCGCTGACGCCATAAAACTCTTCA	IP
Biotin-ND1-R	TGAAGAGTTTTATGGCGTCAGCGAAGGGTTGTAGT	IP

Supplementary Source Data

Supplementary Figure 1

S1g

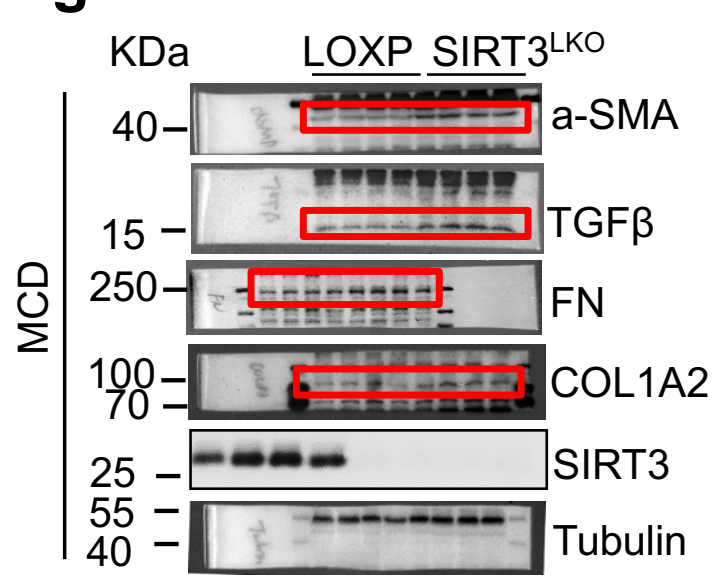

S1h

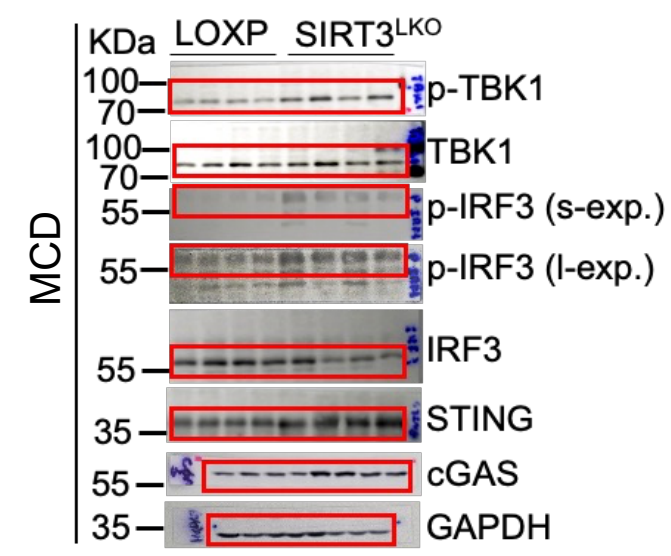

Supplementary Figure 4

S4b

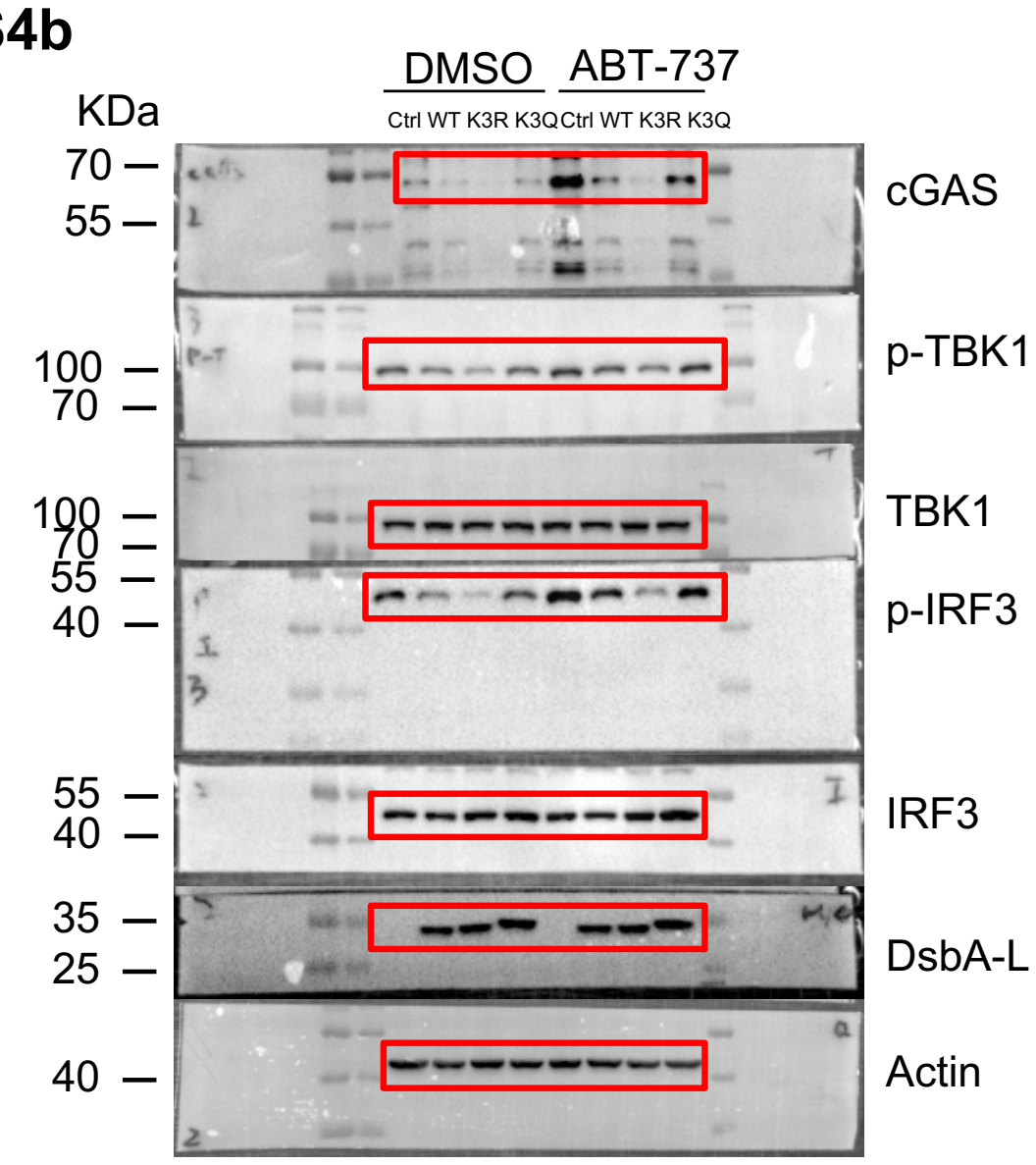

Supplement: Supplementary file 1 [file 41467_2026_72395_MOESM1_ESM.pdf]
