# Supplementary material for: The SIRT3-DsbA-L-TFAM axis restrains cGAS-driven metabolic dysfunction-associated steatohepatitis in male mice
Source: Nat Commun. 2026 May 5;17:8421. doi: 10.1038/s41467-026-72395-8 (PMC13478530; doi:10.1038/s41467-026-72395-8)
Supplement: Supplementary file 3 [file 41467_2026_72395_MOESM3_ESM.pdf]

nature portfolio

Peer Review File

The SIRT3-DsbA-L-TFAM Axis Preserves Mitochondrial DNA Integrity and Restrains cGAS-Driven MASH in Male Mice

Corresponding Author: Dr Juli Bai

This file contains all reviewer reports in order by version, followed by all author rebuttals in order by version.

Version 0:

Reviewer comments:

Reviewer #1

(Remarks to the Author)
Manuscript title: The SIRT3-DsbA-L-TFAM axis is a crucial suppressor of liver cGAS signaling and NASH progression in mice

General comments: In this study the authors aim to identify SIRT3-DsbA-L-TFAM as the regulator for mitochondrial stress cGAS-STING pathway activation in liver. They describe that SIRT3 deacetylates DsbA-L at Lys165, Lys167, and Lys177 inside mitochondria. DsbA-L stabilizes TFAM and enables stability of mitochondrial DNA in the midst of ROS in NASH environment. They utilize liver specific knockout of SIRT3, cGAS and Lysine mutants of DsbAL to elucidate the interplay between these proteins to negatively regulate NASH/fibrosis progression. Though the study seems novel and shows elaborate planning and sufficient invivo and invitro demonstrations of reduced NASH phenotype, there are several issues at exeperimental and molecular level, which needs to be addressed. The expression of STING in hepatocytes has been disputed by various previous studies, which raises a serious question on the validity of the active cGAS-STING-IRF3 pathway in hepatocytes. Also in liver, the Kupffer cells and endothelial cells are known to be the chief source of Interferon production upon stimulus and hepatocytes are not known to produce interferon. The authors need to validate an active STING pathway in hepatocytes and response to high fat metabolism stress using a STING knockout Hepatocyte model with palmitate stimulus and by generating bulk RNA sequence data for differential gene expression.

Specific comments:
1. In Fig1, the authors have not mentioned the duration of the MCD and HF-CD diets.
2. In the western blot Fig1a an d 1g the protein loads are uneven as indicated by Tubulin expression.
3. Fig1c: The blue filter on light source need to be removed before imaging to avoid the orange hue on the image. This makes it difficult to visually evaluate the minor differences in fibrosis. This is also found in various images in the manuscript.
4. Fig1.g TGF beta expression shows increased protein expression with SIRT3 KO but Fig 1e and 1f do not change in TGF beta RNA expression – needs explanation.
5. Fig1h western blots of alpha SMA, FN and Col1A2 do not seem convincing due to low quality.
6. Overall expression of total SIRT3, DsbAL and STING in hepatocytes under palmitate or other NASH stimulus should be compared in Figure 1 prior to any knockdown/knockout experiments.
7. Fig.2 a. The MCD diet for 8 weeks in liver did not induce p-IRF3 but did so only in SIRT3 KO, this is questionable because MCD diet for 8 weeks is harsh enough to induce significant NASH and IRF3 from Kupffer cells and other infiltrating cells. These blots need Flox chow diet control for comparison.
8. Fig3 a. The immunofluorescence is highly unconvincing. The immunofluorescence pattern for DsbA-L and Flag-SIRT3 match exactly which is not possible using antigen antibody reaction. The immunofluorescence images will always show certain minor difference of expression also SIRT3 will be present. Also the experiment does not show any controls or different stimulus conditions.
9. Fig 3. C reduction of SIRT3 in HF-CD diet needs explanation.
10. Fig 4 b, 8 weeks MCD diet is known to induce more severe steatosis and Sirius red staining, the Sirius red staining shown is far less severe.
11. Fig 4.h. TUNEL assay to show mitochondrial DNA damage in liver is not sufficient as it might also indicate apoptosis signals in a NASH liver. The authors need to demonstrate mitoDNA damage with immunofluorescence in isolated hepatocytes form DsbAL-KO mice using mitochondrial and nucleus counter stain.
12. Most of mRNA expression in Fig 5 can be in supplementary data and instead need to include bulk RNA seq for the same

data.
13. Fig.6 c. MCD diet for 8 weeks should induce far severe steatosis and inflammation than shown.
14. Fig. 7. The link between cGAS signaling and hepatic steatosis is not clear. There is not literature evidence for link between cGAS-STING pathway and steatosis upregulation. The possible upregulation of FASN and ACC might be due to other targets of SIRT3 being deactylated. The authors need to clarify this crucial point.

Reviewer #2

(Remarks to the Author)
In this manuscript by Li Hu and co-workers, entitled “The SIRT3-DsbA-L-TFAM Axis is a Crucial Suppressor of Liver cGAS Signaling and NASH Progression in Mice”, authors aimed to elucidate the role of the SIRT3-DsbA-L-TFAM axis in experimental MASH. Results showed that hepatocyte-specific knockout of SIRT3 or DsbA- L in mice promotes mitochondrial DNA release into the cytosol, resulting in activation of the cGAS- STING pathway and exacerbation of MASH. This is a relevant study, as it unveils the mechanisms by which hepatic SIRT3, known to be downregulated in MASH patients, is essential for the preservation of mitochondrial integrity and function. This adds to the relevance of targeting SIRT3 (and/or cGAS) for treating MASH. Specific points could be improved:

1 - A new nomenclature for has NAFLD been advocated by multinational liver societies, with metabolic dysfunction-associated steatotic liver disease (MASLD) and metabolic dysfunction-associated steatohepatitis (MASH) replacing nonalcoholic fatty liver disease (NAFLD) and nonalcoholic steatohepatitis (NASH), respectively (DOI:https://doi.org/10.1016/j.jhep.2023.06.003). I would suggest authors to use the new nomenclature throughout the manuscript.

2 - Can authors elaborate on the reasoning for choosing the high-fat and choline- deficient diet (HF-CD) and methionine-choline-deficient diet (MCD) as the diet models of MASH for this study? These models fail to recapitulate the broad-spectrum characteristics of human MASH comparing to other models (e.g. “western diets”) (DOI: 10.1038/s42255-022-00700-y).
- Also, could the lack of choline (in both models) affect results on mitochondrial dynamics and function?
- Finally, is there also any reasoning for specifically using the MCD model in assessing SIRT3 deficiecy association the cGAS-STING signaling (Figure 2) and as a model of MASH for assesing the effects of fiver-specific DsbA-L knockout (Figure 4), wilhe the HF-CD was used for assessing the interaction between DsbA-L and TFAM (Figure 3c); to test whether DsbA-L acetylation mediates the suppressing effect of SIRT3 on NASH (Figure 5); and to test whether hepatic cGAS knockout alleviates SIRT3 deficiency-induced NASH pathogenesis in mice (Figure 7)?

3 - It would be interesting to see whether the absence of hepatocyte SIRT3 alone (Figure 1); of DsbA-L alone (Figure 4); as well as of cGAS alone (Figure 6), induces changes in the liver phenotype (including steatosis, inflammation and fibrosis), comparing to WT animals (under a control diet).

4 - Concerning liver histology of the different models, it could be relevant if authors quantified steatosis (Oli Red O), fibrosis (Sirirus red staining), hepatocyte ballooning, lobular inflammation and/or the NAS score, rather than solely presenting a representative image. This would be particularly important for the SIRT3/cGAS double knockout mice (where visible differences are more subtle).

5 - Discussion - Several reports have already alluded to the critical role of SIRT3 in NASH development, including manuscript references 15; 16; and 21; but also DOI: 10.1016/j.redox.2018.07.011; DOI: 10.1111/hepr.12634; DOI: 10.1194/jlr.M080952; DOI: 10.1186/s12964-020-00640-8; DOI: 10.1016/j.redox.2018.07.011; and DOI: 10.1002/hep.29229). Authors could better discuss their findings in light of at least some of these previous studies.
- Also, the Sirt3-autophagy duality in the context of MASLD is well established (e.g. DOI: 10.1080/15548627.2023.2254191; DOI: 10.1186/s13020-021-00528-w; DOI: 10.1186/s12986-021-00612-w; DOI: 10.1016/j.redox.2018.07.011; DOI: 10.1002/hep.29229; and DOI: 10.1111/hepr.12634). Could the results presented by the authors in this manuscript add new perspectives to this topic?

Additional comments:

1 - Abstract: Authors state “Here, we identify the SIRT3-DsbA-L-TFAM axis as a crucial suppressor for mitochondrial stress-induced cGAS immune signaling activation in the liver, thereby alleviating nonalcoholic steatohepatitis (NASH) progression in mice.” Given that no immunological studies were performed, this sentence may be misleading. I would suggest deleting “immune signaling”.
Authors also state “Conversely, hepatocyte-specific knockout of cGAS or overexpression of the deacetylation mimic mutant, but not the acetylation mimic mutant, of DsbA-L mitigated NASH diet- or SIRT3 deficiency-induced NASH progression.”. This sentence is somewhat difficult to understand. Perhaps changing to: “or overexpression of DsbA-L mitigated diet- and SIRT3 deficiency-induced NASH progression.”

2 - Figure 2g: Authors state that “DsbA-L deficiency attenuated the inhibitory effect of SIRT3 on ABT-737-induced cGAS-

STING signal activation". However, this is difficult to grasp from the presented panel.

3 - Figure 4 (DsbA-LLKO model) and Figure 6 (cGASLKO model) - Ideally, authors should evaluate the same panel of mRNA and protein fibrogenic markers, as well inflammatory genes, as for the Sirt3LKO model (Figure 1).

4 - Figure 1e-h; j and k - Ideally, the same genes/proteins should be shown in both models; Figure 6f-k - Ideally, the same genes/proteins should be shown in both models

Reviewer #3

(Remarks to the Author)
Hu et al link SIRT3's deacetylase activity to the regulation of interactions between DsbA-L and critical mitochondrial transcriptional regulator TFAM. They go on to show that cGAS-STING inflammatory pathway is activated in the absence of DsbA-L:TFAM interactions, which is otherwise facilitated by the deacetylation of specific lysine residues of DsbA-L by hepatic SIRT3. Authors adequately link SIRT3 deficiency to cGAS-STING signaling; SIRT3-mediated DsbA-L deacetylation to DsbA-L:TFAM interactions; DsbA-L^K3R to mitochondrial dysfunction resulting from hepatic SIRT3 deficiency; and extended the cGAS-STING pathway activation to NASH pathogenesis. However, the biological consequence of the DsbA-L:TFAM interaction is left unexplored and superficial (See Major Comment 1). Data analysis, interpretation, and conclusions are fairly sound; albeit with some gaps in logic: the lack of clear rationale for the direction and the flow of the study (see Major Concerns 1, 2 and 6) can reduce the full objectivity of the study. A key data missing from the manuscript is the increased acetylation of DsbA-L in mice with liver specific deletion of Sirt3 (mice in Figure 1). Because this is anticipated to be the major driver of the newly proposed Sirt3 mechanism, the inclusion of this data is rather essential (Major concern 6).

significance: (high)
Insofar as NASH currently represents a largely unmet challenge to medicine, these results are noteworthy for providing a mechanistic understanding of deleterious hepatic inflammation characteristic of NASH pathogenesis.

Novelty: (medium to high)
Although the SIRT3 deacetylation of DsbA-L and its impact on DsbA-L:TFAM interaction is novel, the link between DsbAL and obesity-induced inflammation via cGAS-STING has been known since 2017. (Bai, J., Cervantes, C., Liu, J., He, S., Zhou, H., Zhang, B., Cai, H., Yin, D., Hu, D., Li, Z., et al. (2017). DsbA-L prevents obesity-induced inflammation and insulin resistance by suppressing the mtDNA release-activated cGAS-cGAMP-STING pathway). This is cited by the authors but does impact the novelty slightly.

Methodology: (appropriate)
The authors support their model using standard techniques consistent with the state-of-the-art in the field including transgenic mouse models and in vitro experiments using cultured mouse primary hepatocytes. The methods section is sufficient and provides the necessary information to likely reproduce the work.

Major Concerns:
1. While DsbA-L binding to TFAM is novel, the impact of this interaction on TFAM activity/stability should be elucidated. Prior reference indicates that TFAM heterozygosity is associated with cytosolic mtDNA, which activates cGAS-STING. Is the rationale for DsbA-L to bind and stabilize TFAM?
2. The leap from SIRT3LKO phenotyping (Figure 1) to cGAS-STING (Figure 2) is abrupt and does not consider other pathways that could be involved. Authors should do better to rule out other links between mitochondrial function and inflammation (NFkB, NLRP3, TLRs, TGFb, PGE2, lipid peroxidation, oxilipins, ROS etc.) or provide rationale for following this path alone. Ideally other pathways would be ruled out leaving only cGAS-STING, which would lead logically to cytosolic mtDNA and the potential involvement of TFAM.
3. In Fig. 2b and d, experiments should be conducted +/- ABT-737 and +/- SIRT3LKO. ABT-737 bypasses the protective effect of SIRT3 on release of mtDNA into the cytosol, therefore, it should nullify loss of SIRT3 or be additive. In either case, cGAS-STING inhibition should reduce Il6 and Il1b induction. To make this less convoluted, SIRT3LKO should be tested with cGAS-STING inhibitors without ABT-737.
4. Figure 3a indicates almost 100% overlap between SIRT3 and DsbA-L in the mitochondrial matrix. The merged figure (yellow) is of uniform intensity throughout which is not to be expected. While technically not impossible, it is surprising to have this level of perfect overlap and raises questions about the methodology used to acquire these images. Authors should provide additional images and ensure proper consideration of analyzed probes and wavelengths.
5. In Fig 3C, input levels of SIRT3 and TFAM in co-IP experiments is affected by HF-CD diet, confounding these results. To properly interpret these results, authors should provide densitometry and normalization to input.
6. The NASH model reduces SIRT3 expression and leads to increased DsbA-L-Ac in WT mice. It's imperative to show that in the SIRT3LKO model with HF-CD (Mice in figure 1), DsbA-L-Ac is higher in the livers of SIRT3LKO mice compared to LOXP controls (WT). Without this data, the rationale for the Sirt3-mediated protection against NASH via DsbA-L deacetylation would not be very strong.

Minor Comments:
1. In Fig.3f,g, interpretation of DsbA-L-Ac upon K3Q over expression is not straightforward. Increased density could be due to the Ab recognizing the Glutamine residue with similar efficacy to that of an acetylated lysine. On the other hand, the Ab might not recognize glutamine at all, and the observed band density could be due to increased lysine acetylation in residues other than 165, 167 and 177.

2. In Fig.5, conclusions could be solidified with the use of a SIRT3;DsbA-L LDKO. Rescue of DsbA-L with a WT or mutant DsbA-L is irrelevant unless the investigation is about assessing dominant negative effect.
3. In Fig.7b, cGAS is not fully ablated. It is clearly knocked down; however, it should be mentioned somewhere in text that cGAS ablation was incomplete.

Reviewer #4

(Remarks to the Author)
I co-reviewed this manuscript with one of the reviewers who provided the listed reports. This is part of the Nature Communications initiative to facilitate training in peer review and to provide appropriate recognition for Early Career Researchers who co-review manuscripts.

Version 1:

Reviewer comments:

Reviewer #1

(Remarks to the Author)
This manuscript is from an experienced group of investigators, and they provide a substantial amount of data. I see this as having two components, a biochemistry component of the proposed interactions between SIRT3, DsbA-1 and TFAM and the relevance of this to the development of MASH. Of the two the second component is novel. For the first component, SIRT3/DsbA-1/TFAM, SIRT3 has previously been shown to increase TFAM activity and protein levels through direct deacetylation (PMID: 30095923).

Regarding the second point that this SIRT3/DsbA-1/TFAM is of relevance in MASH, unfortunately this is almost certainly an artifact caused by their use of two MASH models both of which are based on generating a state of choline deficiency. While it is true that there is no perfect MASH model, and choline deficiency models are in use, the main purpose of using two models is to use different approaches that may compensate for the other's limitations. Using two choline deficiency models is very strange. The issue if however more concrete because it is known that choline is required for the deacetylation function of SIRT3 (PMID: 35457022, PMID: 30165480). This means that many of their manipulations in which increasing SIRT3 function has a protective effect are reversing the known SIRT3 (and other enzymatic) inhibitions caused by choline deficiency. This argument is confirmed by the data from hepatocyte specific SIRT3 deficient mice which do not have increased steatosis in the setting of MASH that is not driven by choline deficiency (PMID: 22645641). The relevant point of-course is that in human MASH there is no deficiency of choline, and the proposed SIRT3/DsbA-1/TFAM pathway is likely not relevant. The authors did discuss the fact their data from choline deficient models is the opposite of the non-choline deficient models but left the question open and suggested theier was equivalence in the two experimental approaches.

Reviewer #2

(Remarks to the Author)
All my comments have been addressed

Reviewer #3

(Remarks to the Author)
In our previous major concern #5, we have emphasized on the fact that there is less SIRT3 and TFAM in the input pool of proteins where DsbA-L is pulled from. The response was that equal amount of DsbA-L was pulled down under all conditions, so the co-IP should have been also equal if the interactions were not weaker.

This rationale is flawed. If there is less SIRT3 and TFAM that can interact with DsbA-L, this may be the reason why less is co-IP'd with equal amounts of DsbA-L. The potential for interaction will be reduced due to the severe drop in the abundance of these interacting proteins. This must be addressed by additional experiments to show that less SIRT3 or TFAM co-IP even when the abundance of these interacting proteins is not different. The case is the same for the revised Fig. 3d (in response to point #6). An alternative option could be by inhibiting acetylation of DsbA-L and conducting IP within the same pool of protein where SIRT3 and TFAM abundance is equal between control and non-acetylated input protein.

If current results cannot be fortified further, this caveat should be included in the text (that this could be due to reduced abundance of the interacting protein).

Version 2:

Reviewer comments:

Reviewer #1

(Remarks to the Author)
All the issues raised have been addressed.

Reviewer #3

(Remarks to the Author)
All the caveats have been successfully addressed

Open Access This Peer Review File is licensed under a Creative Commons Attribution 4.0 International License, which permits use, sharing, adaptation, distribution and reproduction in any medium or format, as long as you give appropriate credit to the original author(s) and the source, provide a link to the Creative Commons license, and indicate if changes were made.
In cases where reviewers are anonymous, credit should be given to 'Anonymous Referee' and the source.
The images or other third party material in this Peer Review File are included in the article’s Creative Commons license, unless indicated otherwise in a credit line to the material. If material is not included in the article’s Creative Commons license and your intended use is not permitted by statutory regulation or exceeds the permitted use, you will need to obtain permission directly from the copyright holder.
To view a copy of this license, visit https://creativecommons.org/licenses/by/4.0/

We thank the reviewers for their constructive comments and valuable suggestions. In response to their comments, we have carried out additional experiments and made careful revisions to the manuscript. Below, we provide detailed, point-by-point responses to each of the reviewers' comments:

Reviewer #1:

I. General comments:

Though the study seems novel and shows elaborate planning and sufficient in vivo and invitro demonstrations of reduced NASH phenotype, there are several issues at experimental and molecular level, which needs to be addressed. The expression of STING in hepatocytes has been disputed by various previous studies, which raises a serious question on the validity of the active cGAS-STING-IRF3 pathway in hepatocytes. Also in liver, the Kupffer cells and endothelial cells are known to be the chief source of Interferon production upon stimulus and hepatocytes are not known to produce interferon. The authors need to validate an active STING pathway in hepatocytes and response to high fat metabolism stress using a STING knockout Hepatocyte model with palmitate stimulus and by generating bulk RNA sequence data for differential gene expression.

Response:
We thank the reviewer for your insightful comments and concerns regarding STING expression in hepatocytes. Although immune cells are the primary producers of interferon, multiple studies have reported low levels of STING expression in hepatocytes or its upregulation under pathological conditions (doi: 10.1016/j.metabol.2017.09.010; doi: 10.1016/j.devcel.2022.06.003; doi: 10.1016/j.bbrc.2020.03.034; doi: 10.1002/hep.32335; doi.org/10.1038/s; doi.org/10.1038/s41598-018-29924-3; doi: 10.1016/j.jhepr.2021.100324). To further validate

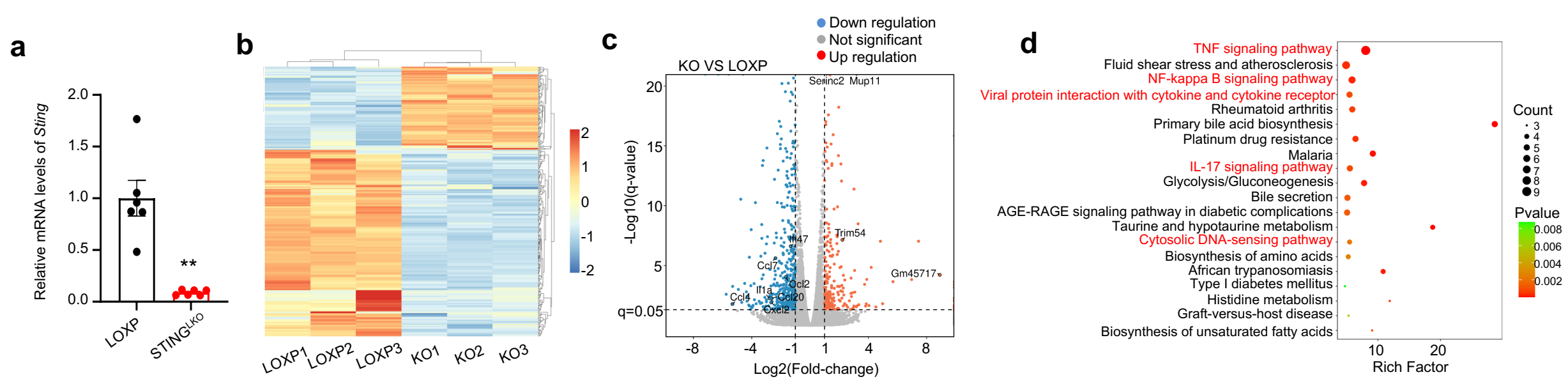

Supplementary Data Fig. 1. The hepatocytes-specific STING knockout ($STING^{LKO}$) mice were generated by crossing STING floxed mice with Albumin-Cre mice. Mouse hepatocytes were purified from both $STING^{Loxp}$ and $STING^{LKO}$ mice, followed by 0.1 mM palmitate treatment for 24 hrs. Cells were collected for RNA sequencing. **a.** STING mRNA expression in purified hepatocytes from $STING^{Loxp}$ and $STING^{LKO}$ mice. **b.** Heatmap showed 97 upregulated genes and 218 downregulated gene in STING-knockout hepatocytes compared to control group. **c**. Vocalo plot of DEGs in control and STING-knockout hepatocytes. **d.** KEGG enrichment analysis made by downregulated DEGs showing pathways including TNF signaling, NF-kappa B signaling, viral protein related to cytokine and cytokine receptor, IL-17 signaling as well as cytosolic DNA sensing pathway were highly enriched.

STING expression in hepatocytes, we generated hepatocyte-specific STING knockout ($STING^{LKO}$) mice by crossing STING floxed mice with Albumin-Cre mice. Mouse hepatocytes were purified from both $STING^{Loxp}$ and $STING^{LKO}$ mice, followed by 0.1 mM palmitate treatment for 24 hrs for *sting* mRNA and bulk RNA-seq analysis. mRNA analysis indicated *sting* was expressed in hepatocytes and the knockout greatly reduced its amount (Supplementary Data Fig. 1a). Heatmap and Volcano plot showed that 97 and 218 genes were upregulated and downregulated, respectively, in STING-knockout hepatocytes compared to the loxp cells (Supplementary Data Fig. 1b, c). Consistent with the finding that *sting* is expressed in hepatocytes, KEGG enrichment analysis showed that inflammation-related pathways, including TNF signaling, NF-kappa B signaling, and viral interaction with cytokine and cytokine receptor pathways, were notably reduced in STING knockout hepatocytes compared to such measures in loxp controls (Supplementary Data Fig. 1b, c). These data support a potential role of STING in mediating inflammatory responses in hepatocytes.

II. Specific comments:

1. In Fig1, the authors have not mentioned the duration of the MCD and HF-CD diets.

Response: We have added this information in the legend of Fig. 1.

2. In the western blot Fig1a and 1g the protein loads are uneven as indicated by Tubulin expression.

Response: We have re-run the western blot, and the loading is now even (Fig. 1c and 1h; previously Fig. 1a and 1g, respectively).

3. Fig1c: The blue filter on light source need to be removed before imaging to avoid the orange hue on the image. This makes it difficult to visually evaluate the minor differences in fibrosis. This is also found in various images in the manuscript.

Response: We have adjusted the color in revised Fig 1e (refer to original submission Fig. 1c), as well as in Fig. 4a, 4k, 5c, and 6d as suggested.

4. Fig1.g TGF beta expression shows increased protein expression with SIRT3 KO but Fig 1e and 1f do not change in TGF beta RNA expression – needs explanation.

Response: We repeated the western blot analysis and consistently observed an increase in TGF-beta protein levels, despite no change in mRNA levels under equal loading conditions (now shown in Fig. 1h). These findings suggest that the elevated TGF-beta protein levels in the livers of $SIRT3^{LKO}$ mice may be due to post-translational regulation, potentially involving changes in protein stability or degradation, rather than transcriptional control. Further investigations are required to explore this compelling possibility.

5. Fig1h western blots of alpha SMA, FN and Col1A2 do not seem convincing due to low quality.

Response: We repeated the western blot experiment and replaced the previous low-quality results with enhanced, more reliable data (now shown in Fig. 1i).

6. Overall expression of total SIRT3, DsbAL and STING in hepatocytes under palmitate or other NASH stimulus should be compared in Figure 1 prior to any knockdown/knockout experiments.

Response: We sincerely appreciate the reviewer's constructive suggestion. In response, we treated wild-type C57BL/6 mice with a CDAHFD (HF-CD) diet and analyzed liver samples from the mice. In mouse MASH liver samples, SIRT3 expression was significantly reduced. Furthermore, SIRT3 protein levels were markedly decreased in primary hepatocytes treated with palmitate and oleic acid (revised Fig. 1a, b). Additionally, we observed a significant downregulation of DsbA-L protein expression, accompanied by a marked upregulation of cGAS and STING in the livers of CDAHFD-fed mice (revised Fig. 2i).

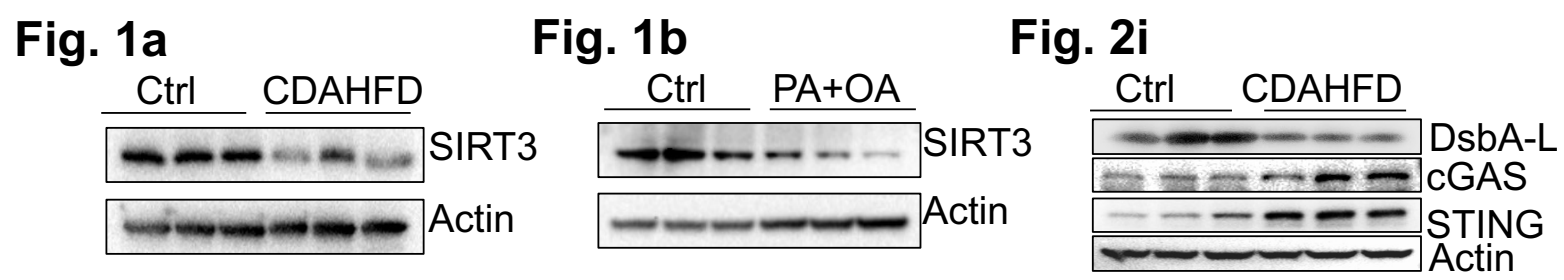

Fig. 1a, Western blot showing the downregulation of SIRT3 protein levels in the livers of CDAHFD (HF-CD) diet-fed wide-type C57BL/6 mice. **Fig. 1b**. The SIRT3 protein level was also greatly reduced in primary hepatocytes treated with palmitate and oleic acid. **Fig. 2i**, Western blot showing the upregulation of cGAS and STING, and down regulation of DsbA-L protein levels in livers of wide-type C57BL/6 mice fed with a CDAHFD (HF-CD) diet for 8 weeks.

7. Fig.2 a. The MCD diet for 8 weeks in liver did not induce p-IRF3 but did so only in SIRT3 KO, this is questionable because MCD diet for 8 weeks is harsh enough to induce significant NASH and IRF3 from Kupffer cells and other infiltrating cells. These blots need Flox chow diet control for comparison.

Response: A normal chow diet does not impact the role of hepatic-SIRT3 in regulating liver metabolism (doi: 10.1038/srep00425), so we excluded normal diet treatment data for Flox and SIRT^{LKO} mice. Due to the high IRF3 phosphorylation in SIRT3 KO mice, the original Fig. 2a featured a shorter exposure blot. However, with longer exposure, we observed substantial IRF3 phosphorylation in the livers of Flox mice as well. This updated, longer-exposure data has been incorporated into the revised Fig. 2a, replacing the previous blot.

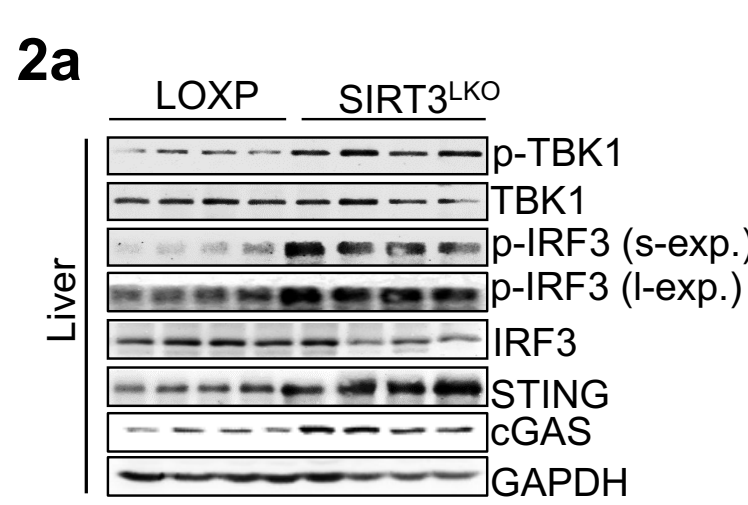

Fig. 2a. The western blot analysis demonstrated a significant increase in cGAS signaling including phosphorylation of TBK1 and IRF3 in liver tissues of SIRT3^{LKO} mice and Loxp control fed with MCD. (s-exp.: Shorter exposure; l-exp. : Longer exposure).

8. Fig3 a. The immunofluorescence is highly unconvincing. The immunofluorescence pattern for DsbA-L and Flag-SIRT3 match exactly which is not possible using antigen antibody reaction. The

immunofluorescence images will always show certain minor difference of expression also SIRT3 will be present. Also the experiment does not show any controls or different stimulus conditions.

Response: We appreciate the reviewer's insightful comment. In our original imaging study, SIRT3 was exogenously expressed, and the observed overlap between DsbA-L and Flag-SIRT3 signals was likely due to signal oversaturation and potential non-specific binding of the Flag antibody. In the revised manuscript, we have now included images showing the colocalization of endogenous DsbA-L and SIRT3 (Fig. 3a). Notably, we observed significant colocalization of these two endogenous proteins in primary hepatocytes from C57BL/6 mice.

9. Fig 3. C reduction of SIRT3 in HF-CD diet needs explanation.

Response: We demonstrated that SIRT3 protein levels were significantly reduced in the livers of CDAHFD (HF-CD) diet-fed mice (revised Fig. 1a, 3c). Additionally, SIRT3 protein levels were also substantially decreased in primary hepatocytes treated with palmitate and oleic acid (revised Fig. 1b). These results strongly support our hypothesis that hepatic SIRT3 deficiency exacerbates liver steatosis, inflammation, and injury, highlighting the critical role of SIRT3 in mitigating MASH progression. The mechanisms underlying the reduction of SIRT3 expression in CDAHFD (HF-CD) diet-induced MASH remain unclear. SIRT3 mRNA expression can be regulated by various transcription factors and co-factors, including positive regulation by ERRα/PGC-1α (PMID: 38261767; PMID: 20661474), NRF2 (PMID: 26109058), and CREBH (PMID: 35926687), or negative regulation by ZEB1 (PMID: 30487699). Additionally, SIRT3 protein can undergo post-translational modifications such as glutathionylation (PMID: 28684630), sulfhydration (PMID: 31218880), and nitration (PMID: 29721150). The reduction of SIRT3 in response to the CDAHFD (HF-CD) diet may be due to altered activity or expression of these transcriptional regulators, as well as changes in post-translational modifications, ultimately leading to decreased SIRT3 levels.

10. Fig 4 b, 8 weeks MCD diet is known to induce more severe steatosis and Sirius red staining, the Sirius red staining shown is far less severe.

Response: Thank you for highlighting this point. Consistent with the well-established role of an 8-week MCD diet in more severe steatosis and fibrosis, in the revised manuscript we have replaced the previous image with a more representative one, showing more pronounced steatosis in the livers of Loxp mice, with even more severe steatosis observed in the livers of DsbA-L^{LKO} mice (revised Fig. 4a).

11. Fig 4.h. TUNEL assay to show mitochondrial DNA damage in liver is not sufficient as it might also indicate apoptosis signals in a NASH liver. The authors need to demonstrate mitoDNA damage with immunofluorescence in isolated hepatocytes form DsbAL-KO mice using mitochondrial and nucleus counter stain.

Response: Thank you for the excellent suggestion. We have performed an 8-OH-dG assay to assess oxidative damage to mitochondrial DNA in primary hepatocytes from DsbA-L^{LKO} and Loxp control mice. Our results indicate that the knockout of DsbA-L in hepatocytes significantly increased DNA damage, as evidenced by strong immunoreactivity for 8-OH-dG (revised Fig. 4h).

12. Most of mRNA expression in Fig 5 can be in supplementary data and instead need to include bulk RNA seq for the same data.

Response: As per your suggestion, we have relocated most of the mRNA data from Fig. 5 to the supplementary section (now revised as Supplementary Data Fig. 4f-q). However, we retained the

original Fig. 5a-c, now presented as Fig. 4k-m in the revised version, as these findings are critical for validating our *in vitro* observations. They demonstrate that different DsbA-L mutants have distinct physiological roles in regulating MASH phenotypes and cGAS-STING signaling *in vivo*. Although bulk RNA-Seq would indeed provide deeper mechanistic insights into how different DsbA-L mutants affect MASH phenotypes and liver metabolism, the focus of our current study is primarily on SIRT3 acetylation in MASH.

13. Fig.6 c. MCD diet for 8 weeks should induce far severe steatosis and inflammation than shown.

Response: We thank the reviewer for this insightful comment. In response, we have replaced the previous data with more representative H&E and Oil red O staining images that clearly illustrate steatosis in both Loxp and $cGAS^{LKO}$ mice. Notably, compared to the Loxp control group, $cGAS^{LKO}$ mice showed a significant reduction in both steatosis and fibrosis under MCD and CDAHFD (HF-CD) feeding conditions (revised Fig. 5e). Even though Sirus Red staining is not ideal for visualizing steatosis, lipid droplet accumulation is still evident under both MCD and CDAHFD conditions (revised Fig. 5c).

14. Fig. 7. The link between cGAS signaling and hepatic steatosis is not clear. There is not literature evidence for link between cGAS-STING pathway and steatosis upregulation. The possible upregulation of FASN and ACC might be due to other targets of SIRT3 being deactylated. The authors need to clarify this crucial point.

Response: We sincerely thank the reviewer for raising this important question. Although we acknowledge that the connection between cGAS-STING signaling and hepatic steatosis is still an emerging area of research, there are several lines of evidence supporting its involvement in lipid metabolism regulation.

First, recent studies have shown that STING plays a role beyond its classical function in innate immunity. It has been demonstrated to interact with and modulate the activity of fatty acid desaturase 2 (FADS2), which is essential for polyunsaturated fatty acid (PUFA) desaturation. This interaction may influence lipid metabolism independently of the immune-related functions of STING (PMID: 34986331). This suggests that STING has the potential to impact lipid metabolism pathways, which could be relevant to hepatic steatosis.

Second, supporting evidence from Drosophila models has shown that STING deletion leads to reduced lipid storage and a significant downregulation of lipid metabolism-related genes, including fatty acid synthase (FASN) and acetyl-CoA carboxylase (ACC) (PMID: 34467853). These findings underscore the critical role of the cGAS-STING pathway in the regulation of lipid metabolism. Consistent with these studies, we observed a significant reduction in lipid accumulation in liver when cGAS was knocked out in our murine model (Fig. 5e).

Furthermore, in $SIRT3^{LKO}$ mice, cGAS deletion led to reduced liver steatosis and the downregulation of lipogenic genes such as FASN and ACC (Figs. 6d, h). These results provide strong evidence that cGAS contributes to the development of steatosis in the context of SIRT3 deficiency under MASH conditions.

Although we acknowledge that SIRT3 may influence lipid metabolism through other targets, such as its deacetylation of key metabolic enzymes, our findings highlight the important role of cGAS in mediating lipid accumulation in SIRT3-deficient livers. This suggests a novel link between cGAS-STING signaling and hepatic steatosis, which warrants further investigation in future studies.

We have incorporated these discussions into the revised manuscript (Page 14, Lines 367-380). We hope that this clarifies the connection between cGAS signaling and hepatic steatosis, and we look forward to the reviewer's feedback.

Reviewer #2:

1. - A new nomenclature for has NAFLD been advocated by multinational liver societies, with metabolic dysfunction-associated steatotic liver disease (MASLD) and metabolic dysfunction-associated steatohepatitis (MASH) replacing nonalcoholic fatty liver disease (NAFLD) and nonalcoholic steatohepatitis (NASH), respectively (DOI: 10.1016/j.jhep.2023.06.003). I would suggest authors to use the new nomenclature throughout the manuscript.

Response: We appreciate the reviewer's constructive suggestion. In response, we have revised the manuscript to adopt the updated terminology of metabolic dysfunction-associated steatotic liver disease (MASLD) and metabolic dysfunction-associated steatohepatitis (MASH) in place of the terms nonalcoholic fatty liver disease (NAFLD) and nonalcoholic steatohepatitis (NASH). This change has been consistently applied throughout the manuscript to reflect the most current nomenclature and ensure clarity for the readers.

2. - Can authors elaborate on the reasoning for choosing the high-fat and choline- deficient diet (HF-CD) and methionine-choline-deficient diet (MCD) as the diet models of MASH for this study? These models fail to recapitulate the broad-spectrum characteristics of human MASH comparing to other models (e.g. "western diets") (DOI: 10.1038/s42255-022-00700-y).
- Also, could the lack of choline (in both models) affect results on mitochondrial dynamics and function?
- Finally, is there also any reasoning for specifically using the MCD model in assessing SIRT3 deficiecy association the cGAS-STING signaling (Figure 2) and as a model of MASH for assesing the effects of fiver-specific DsbA-L knockout (Figure 4), wilhe the HF-CD was used for assessing the interaction between DsbA-L and TFAM (Figure 3c); to test whether DsbA-L acetylation mediates the suppressing effect of SIRT3 on NASH (Figure 5); and to test whether hepatic cGAS knockout alleviates SIRT3 deficiency-induced NASH pathogenesis in mice (Figure 7)?

Response: We initiated our project at a time when the methionine-choline-deficient (MCD) diet was the primary model for inducing metabolic dysfunction-associated steatohepatitis (MASH) in mice. As research progressed and more refined models became available, we transitioned to using the choline-deficient, L-amino acid-defined, high-fat diet (CDAHFD) (PMID: 36539621, DOI: 10.1038/s42255-022-00700-y, Research Diets Inc., Cat# A06071302). Initially, we referred to this diet as the high-fat and choline-deficient diet (HF-CD) in our manuscript. To prevent any potential confusion, we have now updated the terminology to CDAHFD in the revised version.

As noted in the literature, "there is no 'perfect' model of NASH" (PMID: 36539621, DOI: 10.1038/s42255-022-00700-y). We selected CDAHFD to accelerate MASH progression in lean mice, allowing us to investigate the role of SIRT3. MASH is a complex disease, and clinical features can vary significantly among individuals. Even though CDAHFD may not replicate all aspects of human MASH, other models, such as the Western diet or GAN diet, also have limitations, including excessively high cholesterol levels that far exceed recommended human intake. Both MCD and CDAHFD yielded similar conclusions regarding MASH phenotypes and cGAS signaling activation, which justifies the use of both diets in our studies.

Regarding choline's role, both the MCD and CDAHFD diets are choline deficient. Choline is known to be essential for mitochondrial function, influencing processes such as purine nucleotide regulation, respiratory capacity, mitochondrial phosphatidylcholine synthesis, and ATP production (PMID: 39111307; PMID: 3826464; PMID: 30982734). Therefore, it is likely that both diets impact mitochondrial function. Although our current study does not focus on choline's effects, we recognize this as a promising area for future research and have discussed it in our revised manuscript (Page 14-15, Lines 393-402).

3 - It would be interesting to see whether the absence of hepatocyte SIRT3 alone (Figure 1); of DsbA-L alone (Figure 4); as well as of cGAS alone (Figure 6), induces changes in the liver phenotype (including steatosis, inflammation and fibrosis), comparing to WT animals (under a control diet).

Response: We appreciate the reviewer's suggestion to assess phenotype changes in liver in the absence of hepatocyte SIRT3, DsbA-L, and cGAS compared to those in WT animals under a control diet. Previous studies have already established that SIRT3 knockout ($SIRT3^{LKO}$) mice exhibit a normal metabolic phenotype when fed either a chow or high-fat diet (PMID: 22645641). Given these well-documented findings, we did not repeat these experiments. Indeed, our prior research demonstrated that DsbA-L knockout ($DsbA\text{-}L^{LKO}$) mice showed no significant differences in metabolic phenotype compared to their loxp controls under normal diet conditions (PMID: 28232481). Consistent with these findings, $cGAS^{LKO}$ mice exhibited no significant differences in body weight, food intake, fasting glucose levels, or insulin and glucose tolerance compared to floxed control mice on a standard chow diet (These results will be detailed in a forthcoming publication).

4 - Concerning liver histology of the different models, it could be relevant if authors quantified steatosis (Oli Red O), fibrosis (Sirirus red staining), hepatocyte ballooning, lobular inflammation and/or the NAS score, rather than solely presenting a representative image. This would be particularly important for the SIRT3/cGAS double knockout mice (where visible differences are more subtle).

Response: We thank the reviewer for this valuable suggestion. We have quantified steatosis, fibrosis, hepatocyte ballooning, and lobular inflammation using the MASLD Activity Score (MAS). The quantification data are included in the revised manuscript (Fig. 1l, 4c, 5f and 6e).

5 - Discussion - Several reports have already alluded to the critical role of SIRT3 in NASH development, including manuscript references 15; 16; and 21; but also DOI: 10.1016/j.redox.2018.07.011; DOI: 10.1111/hepr.12634; DOI: 10.1194/jlr.M080952; DOI: 10.1186/s12964-020-00640-8; DOI: 10.1016/j.redox.2018.07.011; and DOI: 10.1002/hep.29229). Authors could better discuss their findings in light of at least some of these previous studies.
- Also, the Sirt3-autophagy duality in the context of MASLD is well established (e.g. DOI: 10.1080/15548627.2023.2254191; DOI: 10.1186/s13020-021-00528-w; DOI: 10.1186/s12986-021-00612-w; DOI: 10.1016/j.redox.2018.07.011; DOI: 10.1002/hep.29229; and DOI: 10.1111/hepr.12634). Could the results presented by the authors in this manuscript add new perspectives to this topic?

Response: Thank you for listing these references and your comments, which are truly helpful for improving our understanding of the specific role of SIRT3 in regulating MASH and lipid metabolism. In our revised manuscript, we have discussed these important studies:

SIRT3 is known to regulate glucose metabolism, mitochondrial homeostasis, and cellular stress resistance (doi.10.1056/NEJMra1100831; doi.10.1016/j.tem.2015.06.001; doi.10.3389/fendo.2019.00187), yet its specific role in MASLD remains unclear. Both global knockout and overexpression of SIRT3 have been linked to MASLD, with global SIRT3 knockout accelerating and SIRT3 overexpression decelerating obesity, insulin resistance, hepatosteatosis, inflammation, hepatocyte death, and fibrosis in mice fed HFD or MCD diets (doi.10.1016/j.molcel.2011.07.019; doi.10.1002/mnfr.201800612; doi. 10.1038/nature08778; doi.10.1186/s12964-020-00640-8; doi. 10.3390/ijms22084277; doi. 10.1128/MCB.01658-12). However, the role of hepatic SIRT3 is less defined. For example, Fernández-Marcos et al. found that SIRT3LKO mice fed an HFD did not exhibit the mitochondrial dysfunction and metabolic abnormalities seen in global SIRT3 knockouts, despite increased mitochondrial protein hyperacetylation (doi.10.1038/srep00425). Similarly, hepatic SIRT3 overexpression via HTVI had limited effects on substrate metabolism and did not prevent HFD-induced metabolic defects (doi.10.1016/j.bbrc.2022.03.088). Interestingly, hepatic SIRT3 alleviated steatosis in mitochondrial trifunctional protein knockout ($MTP^{+/-}$) mice under HFD but had minimal impact on hepatic triglycerides in wild-type mice (doi.10.1194/jlr.M080952). In other studies, hepatic SIRT3 overexpression alleviated MCD-induced MASH in AhR-transgenic mice, though no wild-type comparisons were included (doi.10.1128/MCB.01658-12). Contradicting findings by Li et al. (doi. 10.1128/MCB.01658-12) suggested that hepatic SIRT3 overexpression exacerbated liver damage in mice on an SFA-rich diet, potentially through AMPK suppression and reduced autophagy. Given that HFD is not an optimal model for inducing MASH, the role of hepatic SIRT3 in MASH protection remains unresolved. In this study, we demonstrated that hepatocyte-specific SIRT3 knockout worsened MCD or CDAHFD-induced MASH, revealing a protective role for hepatic SIRT3 in MASH development through a novel mitochondrial stress-induced cGAS signaling mechanism. (Page 12-13, lines 332-355)

Interestingly, both SIRT3 (doi.10.1186/s13020-021-00528-w; doi.10.1016/j.redox.2018.07.011; doi. 10.1038/s41418-019-0356-z) and the cGAS-STING pathway [18,54-57] (doi.10.2337/dbi18-0052; doi. 10.1016/j.cell.2012.06.040; doi.10.4161/auto.28769; doi.10.1038/s41586-019-1006-9; doi.10.1038/s41586-018-0448-9; DOI: 10.1080/15548627.2021.1961072) are involved in regulating autophagy, including lipophagy and mitophagy. SIRT3 is primarily known to promote autophagy through pathways such as AMPK/mTORC1, FOXO1, Pink/Parkin, and ERK/CREB (doi.10.1186/s13020-021-00528-w; doi.10.1016/j.redox.2018.07.011; doi.10.1038/s41418-019-0356-z; (doi.org/10.1186/s13008-024-00124-y; DOI: 10.3390/ijms21020372). However, Li et al. (doi: 10.1002/hep.29229) reported a negative role for SIRT3 in autophagy. Meanwhile, cGAS and STING promote autophagy via Beclin-1, ULK1, and TBK1 activation (doi.10.1038/s41586-019-1006-9; doi.10.1016/j.cell.2013.09.049; doi: 10.2337/dbi18-0052), but fatty acid-induced cGAS-STING signaling has been shown to activate mTORC1, which inhibits autophagy and lipophagy in fatty liver (doi.10.1080/15548627.2021.1961072). Since autophagy and lipophagy are critical for triglyceride breakdown and lipid droplet degradation, SIRT3 may reduce lipid accumulation by promoting lipophagy while simultaneously suppressing cGAS-STING signaling. This dual regulation could help protect against hepatosteatosis in MASH. Further investigation into the interaction between cGAS and SIRT3-driven autophagy, especially in lipid metabolism, is needed to clarify their roles in MASH. (Page 14, lines 380-392)

Additional comments:

1 - Abstract: Authors state "Here, we identify the SIRT3-DsbA-L-TFAM axis as a crucial suppressor for mitochondrial stress-induced cGAS immune signaling activation in the liver, thereby alleviating nonalcoholic steatohepatitis (NASH) progression in mice." Given that no

immunological studies were performed, this sentence may be misleading. I would suggest deleting "immune signaling".
Authors also state "Conversely, hepatocyte-specific knockout of cGAS or overexpression of the deacetylation mimic mutant, but not the acetylation mimic mutant, of DsbA-L mitigated NASH diet- or SIRT3 deficiency-induced NASH progression.". This sentence is somewhat difficult to understand. Perhaps changing to: "or overexpression of DsbA-L mitigated diet- and SIRT3 deficiency-induced NASH progression."

Response: Thank you for the constructive suggestion. In response, we have replaced the original sentence with "Here, we identify the SIRT3-DsbA-L-TFAM axis as a crucial suppressor for mitochondrial stress-induced cGAS activation in the hepatocytes, thereby alleviating MASH in mice". We have also replaced the previous statement with "overexpression of DsbA-L mitigated diet- and SIRT3 deficiency-induced MASH progression," as recommended.

2 - Figure 2g: Authors state that "DsbA-L deficiency attenuated the inhibitory effect of SIRT3 on ABT-737-induced cGAS- STING signal activation". However, this is difficult to grasp from the presented panel.

Response: Thank you for your valuable feedback on Figure 2g. In response, we conducted additional experiments using both RNAi-mediated DsbA-L knockdown and primary hepatocytes isolated from DsbA-L^{LKO} and loxp mice. Our consistent findings demonstrate that DsbA-L deficiency attenuates SIRT3's inhibitory effect on ABT-737-induced cGAS-STING signaling activation (revised Fig. 2g). We have highlighted the relevant panel for clarity. Additionally, we have included a quantitative analysis of the results to further support our conclusions. We hope this revision addresses your concerns and enhances the clarity of our findings.

Fig. 2g

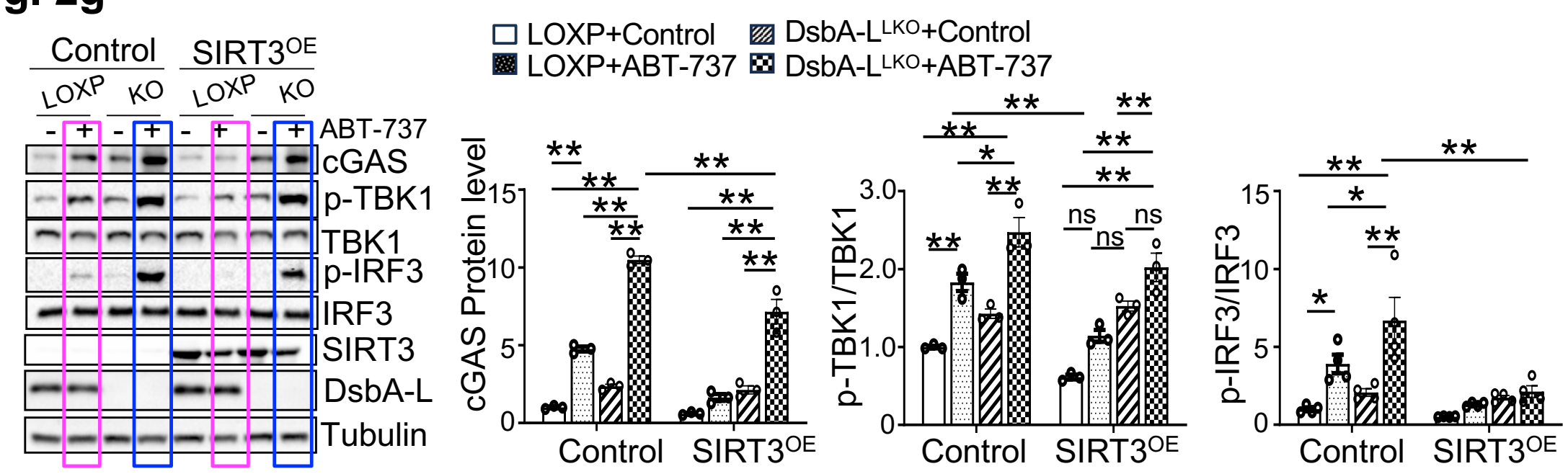

Fig. 2g. Immunoblot analysis and corresponding quantification of cGAS-STING signaling molecule expression and phosphorylation in DsbA-L knockout hepatocytes, with or without SIRT3 overexpression, and treated with or without 10 μM ABT-737 for 16 hours. In all statistical plots, data are presented as mean ± SEM, *, *P* < 0.05; **, *P* < 0.01.

3 - Figure 4 (DsbA-LLKO model) and Figure 6 (cGASLKO model) - Ideally, authors should evaluate the same panel of mRNA and protein fibrogenic markers, as well inflammatory genes, as for the Sirt3LKO model (Figure 1).

Response: Thank you for your insightful feedback. This project was initiated some time ago, and we have incorporated different inflammatory and fibrogenic markers as corresponding primers

became available. Although the specific markers may vary across figures, the overall conclusions—drawn from mRNA and protein expression levels, alongside histological analyses—remain consistent and robust. We believe these data collectively support our key findings.

4 - Figure 1e-h; j and k - Ideally, the same genes/proteins should be shown in both models; Figure 6f-k - Ideally, the same genes/proteins should be shown in both models

Response: Thank you once again for your thoughtful feedback. As mentioned previously, although the specific markers may differ slightly between the figures, the overarching conclusions drawn from mRNA and protein expression levels, as well as histological analyses, remain consistent and strongly support our findings.

Reviewer #3 (Remarks to the Author):

We were very pleased to know that our work was considered to be novel and significant. We also thank the reviewer for their constructive comments and valuable suggestions. Our answers to the points raised are below:

Major Concerns:

1. While DsbA-L binding to TFAM is novel, the impact of this interaction on TFAM activity/stability should be elucidated. Prior reference indicates that TFAM heterozygosity is associated with cytosolic mtDNA, which activates cGAS-STING. Is the rationale for DsbA-L to bind and stabilize TFAM?

Response: We thank the reviewer for this insightful question. Unraveling the rationale behind DsbA-L's interaction with TFAM and its role in TFAM dysfunction-induced activation of the cGAS-STING signaling is indeed a key focus of our study. Our findings indicate that this interaction is regulated by SIRT3, with DsbA-L acetylation playing a pivotal role (see revised Figs. 3d-e, 3g-h). Given that TFAM, an abundant mtDNA-binding protein, controls nucleoid architecture, mtDNA synthesis, and transcription (PMID: 25642965), its binding to mitochondrial DNA is critical for its activity. Our data show that, in DsbA-L knockout or acetylation mimicking mutant DsbA-L expressing hepatocytes, TFAM's binding to mtDNA is significantly reduced compared to wild-type DsbA-L expressing hepatocytes—despite stable TFAM protein levels. This suggests that DsbA-L, particularly its acetylation, influences TFAM's activity rather than its stability (Fig. 3i). Moreover, heterozygous deletion of TFAM has been shown to activate significantly the cGAS-STING signaling pathway (PMID: 25642965). Thus, the reduced binding of TFAM to mtDNA in DsbA-L deficiency or acetylation conditions may explain the link between SIRT3 deficiency and the activation of the cGAS-STING signaling. We have elaborated on this mechanism in the Discussion section of the revised manuscript (Page 11, Lines 285-294) and believe this clarifies the significance of the DsbA-L–TFAM interaction in our study.

2. The leap from SIRT3LKO phenotyping (Figure 1) to cGAS-STING (Figure 2) is abrupt and does not consider other pathways that could be involved. Authors should do better to rule out other links between mitochondrial function and inflammation (NFkB, NLRP3, TLRs, TGFb, PGE2, lipid peroxidation, oxilipins, ROS etc.) or provide rationale for following this path alone. Ideally other pathways would be ruled out leaving only cGAS-STING, which would lead logically to cytosolic mtDNA and the potential involvement of TFAM.

Response: We appreciate the reviewer for highlighting the need to explore the connections between mitochondrial function and inflammation pathways.

It is known that mitochondrial components, such as mtDNA, ROS, and ATP, can act as danger signals that promote inflammation when released into the cytosol (doi.org/10.1038/s41577-022-00760-x). Specifically, mtDNA can activate the cGAS-STING or TLR9 signaling pathways, both of which trigger NFκB inflammatory and type I interferon responses. Another key inflammatory pathway driven by mitochondrial factors is the NLRP3 inflammasome pathway, which can be activated by mtDNA, ATP and ROS. Whereas it is possible that SIRT3 deficiency may dysregulate cell function by activating multiple inflammatory pathways, we found a significant increase in cytosolic mtDNA content and activation of cGAS-STING signaling pathway evidenced by enhanced phosphorylation of TBK1 and IRF3 in hepatocytes and livers deficient in SIRT3 (Fig. 2a-2c). More importantly, suppressing cGAS or STING activities markedly reduced inflammatory gene expression in SIRT3-deficient hepatocytes and livers (Fig. 2d and Fig. 6g) and alleviated TBK1 and NFκB activation, liver steatosis, injury, inflammation, and fibrosis in mice (Fig. 6b-6h). We cannot rule out the potential contributions of other mitochondrial dysfunction-induced inflammatory pathways, such as TLR9 or NLRP3, particularly given the observed increases in mtDNA release and mtROS in SIRT3-deficient hepatocytes (Fig. 2c; Supplementary Data Fig. 4d), but our findings suggest that cGAS-STING signaling likely plays a predominant role in driving inflammation in this context. Nevertheless, further studies are needed to clarify the specific roles of TLRs and the NLRP3 inflammasome in this process. We have discussed this in our revised manuscript (Page 12, lines 315-331).

3. In Fig. 2b and d, experiments should be conducted +/- ABT-737 and +/- SIRT3LKO. ABT-it737 bypasses the protective effect of SIRT3 on release of mtDNA into the cytosol, therefore, it should nullify loss of SIRT3 or be additive. In either case, cGAS-STING inhibition should reduce Il6 and Il1b induction. To make this less convoluted, SIRT3LKO should be tested with cGAS-STING inhibitors without ABT-737.

Response: Thank you for your insightful comments. We carried out experiments under both ABT-737-stimulated and unstimulated conditions. Notably, we observed no significant changes in Il6 or Il1β mRNA expression in SIRT3-deficient hepatocytes without ABT-737 stimulation (revised Fig. 2d). However, ABT-737 bypassed SIRT3's regulatory influence on cGAS signaling (revised Fig. 2g) and inflammatory gene expression (revised Fig. 2d) in control hepatocytes. Importantly, our results show that SIRT3 knockout significantly accelerates, but SIRT3 overexpression attenuates, ABT-737-induced cGAS signaling activation and inflammatory gene expression. These findings suggest that SIRT3 synergizes with Bcl-2 to inhibit mtDNA release and subsequent cGAS activation. An exciting direction for future research would be to investigate whether SIRT3 regulates Bcl-2 and Bax/Bak pore formation, and if this interaction depends on DsbA-L. We plan to explore these intriguing possibilities further.

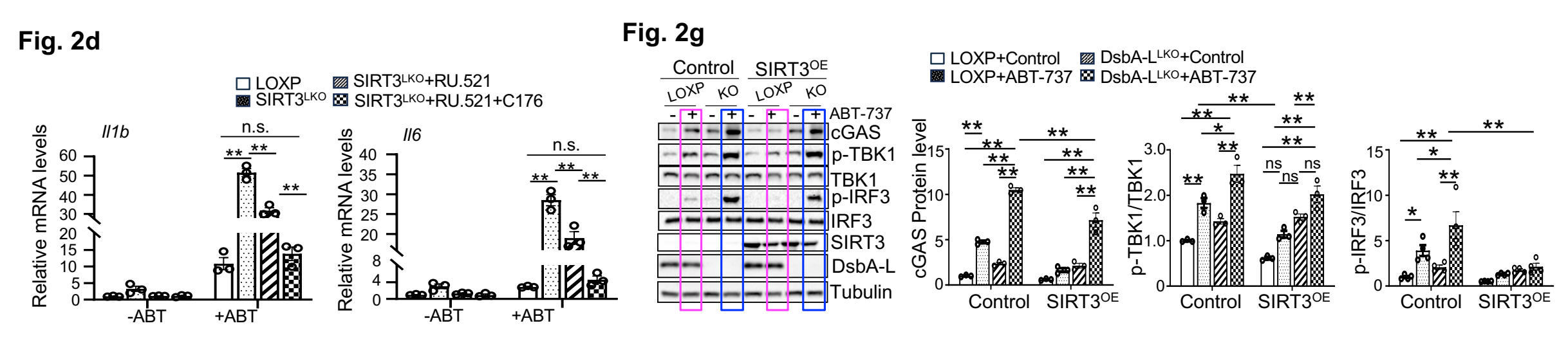

Fig. 2d, mRNA expression of inflammatory genes in SIRT3 knockout or loxp primary hepatocytes pre-treated with either the cGAS inhibitor RU.521 (5 μM) or the STING inhibitor C176 (5 μM) for 2 hrs, followed by treatment with 10 μM ABT-737 for 24 hrs. **2g**, Immunoblot analysis and corresponding quantification of DsbA-L knockout hepatocytes overexpressing SIRT3 or control plasmid, with or without 10 μM ABT-737 treatment for 16 hours. All statistical data are presented as mean ± SEM.*, *P* < 0.05; **, *P* < 0.01.

4. Figure 3a indicates almost 100% overlap between SIRT3 and DsbA-L in the mitochondrial matrix. The merged figure (yellow) is of uniform intensity throughout which is not to be expected. While technically not impossible, it is surprising to have this level of perfect overlap and raises questions about the methodology used to acquire these images. Authors should provide additional images and ensure proper consideration of analyzed probes and wavelengths.

Response: Thank you for your insightful comment. We have addressed this point in our response to Reviewer 1, specifically in response #8.

5. In Fig 3C, input levels of SIRT3 and TFAM in co-IP experiments is affected by HF-CD diet, confounding these results. To properly interpret these results, authors should provide densitometry and normalization to input.

Response: We acknowledge that SIRT3 and TFAM expression were downregulated in MASH livers under the CDAHFD (HF-CD) diet (revised Fig. 3c), consistent with our findings in Figs. 1a-1b. However, since the Co-IP experiments were performed with equal amounts of DsbA-L antibodies, the immunoprecipitated DsbA-L levels remained relatively consistent. Despite this, the binding of SIRT3 and TFAM to DsbA-L was significantly reduced, likely due to increased acetylation of DsbA-L. This suggests that under CDAHFD (HF-CD) conditions, the reduced expression or activity of SIRT3 enhances DsbA-L acetylation, thereby weakening its interaction with TFAM. To support this, we have included densitometry data normalized to the immunoprecipitated DsbA-L levels (revised Fig. 3c).

6. The NASH model reduces SIRT3 expression and leads to increased DsbA-L-Ac in WT mice. It's imperative to show that in the SIRT3LKO model with HF-CD (Mice in figure 1), DsbA-L-Ac is higher in the livers of SIRT3LKO mice compared to LOXP controls (WT). Without this data, the rationale for the Sirt3-mediated protection against NASH via DsbA-L deacetylation would not be very strong.

Response: We appreciate the reviewer's constructive suggestion. We carried out Co-IP experiments in livers from $SIRT3^{LKO}$ and Loxp control mice fed a CDAHFD (HF-CD) diet. Our results demonstrate that DsbA-L acetylation is significantly increased in the livers of $SIRT3^{LKO}$ mice compared to that in Loxp controls. This increased acetylation correlates with a decreased interaction between DsbA-L and TFAM, as shown in revised Fig. 3d. These findings strengthen the rationale for SIRT3-mediated protection against NASH through the deacetylation of DsbA-L.

Fig. 3d

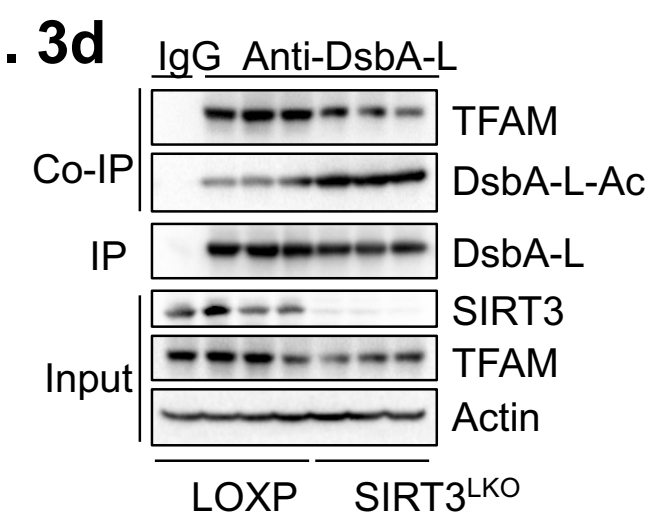

Fig. 3d. The interaction of DsbA-L with SIRT3 or TFAM in liver tissues from CDAHFD fed $SIRT3^{LKO}$ and loxp control mice was determined by co-immunoprecipitation assay.

Minor Comments:

1. In Fig.3f, g, interpretation of DsbA-L-Ac upon K3Q over expression is not straightforward. Increased density could be due to the Ab recognizing the Glutamine residue with similar efficacy to that of an acetylated lysine. On the other hand, the Ab might not recognize glutamine at all, and the observed band density could be due to increased lysine acetylation in residues other than 165, 167 and 177.

Response: We appreciate the reviewer's insightful comments. Our results show an increased density of acetylated DsbA-L (DsbA-L-Ac) when lysine residues 165, 167, and 177 were mutated to glutamine (K3Q, an acetylation mimic) compared to the arginine mutation (K3R, a deacetylation mimic), while total DsbA-L protein levels remained unchanged in both conditions. Although these three lysines are the primary deacetylation targets of DsbA-L, some acetylation was still detectable in the K3R variant, suggesting that other lysine residues may also be subject to acetylation (Fig. 3f, g). Importantly, the acetylation level of K3R-DsbA-L was unaffected by SIRT3 knockout or overexpression, confirming that these specific sites are not regulated by SIRT3. The increased acetylation observed in K3Q-DsbA-L may be due to a conformational change induced by the mutation. This alteration could expose lysine residues, other than 165, 167 and 177, to mitochondria, a high-acetyl environment, making them more susceptible to acetylation. However, we also cannot exclude the possibility of the acetylation antibody recognizing the glutamine residue in DsbA-L protein.

2. In Fig.5, conclusions could be solidified with the use of a SIRT3; DsbA-L LDKO. Rescue of DsbA-L with a WT or mutant DsbA-L is irrelevant unless the investigation is about assessing dominant negative effect.

Response: We thank the reviewer for this thoughtful point. However, considering that both SIRT3 and DsbA-L deficiencies result in more severe MASH phenotypes, we believe that a double knockout of these genes would likely exacerbate MASH, making it difficult to discern whether DsbA-L or its acetylation status mediates the SIRT3 deficiency-induced phenotypes. Instead, overexpressing wild-type or mutant DsbA-L in SIRT3 knockout mice offers a clearer approach to determining the role of DsbA-L acetylation in SIRT3's function. Indeed, our findings show that the DsbA-L K3R (deacetylation mimic) mutant significantly alleviated SIRT3 deficiency-induced MASH, whereas the DsbA-L K3Q (acetylation mimic) mutant did not (revised Fig. 4k-m; Supplementary Data Fig. 4f-q). Furthermore, in SIRT3/cGAS double knockout (SIRT3/cGASL^{DKO}) mice, we observed a marked reduction in MASH progression, further supporting the protective role of DsbA-L deacetylation and its suppression of cGAS activation (revised Fig. 6b-h). Together, these results underscore the importance of DsbA-L deacetylation in mediating SIRT3's protective effects against MASH.

3. In Fig.7b, cGAS is not fully ablated. It is clearly knocked down; however, it should be mentioned somewhere in text that cGAS ablation was incomplete.

Response: The liver is a heterogeneous tissue composed not only of hepatocytes but also various other cell types, including immune cells. Since cGAS is highly expressed in immune cells, we observed a reduction, rather than a complete loss, of cGAS protein in liver tissues (revised Figs. 5a, 6b). To confirm that cGAS is specifically knocked out in hepatocytes, we isolated hepatocytes from the livers of both cGAS^{LDKO} and SIRT3/cGAS^{LDKO} mice. Western blot analysis clearly demonstrated the absence of cGAS in primary hepatocytes isolated from these knockout mice (revised Figs. 5b, 6a).

Reviewer #4 (Remarks to the Author):

I co-reviewed this manuscript with one of the reviewers who provided the listed reports. This is part of the Nature Communications initiative to facilitate training in peer review and to provide appropriate recognition for Early Career Researchers who co-review manuscripts.

Response: We sincerely appreciate your valuable insights and constructive suggestions, which have greatly contributed to improving the quality of our manuscript.

Dear Reviewers:

We are deeply grateful for your thoughtful critiques and insightful suggestions. Your expertise and constructive feedback have greatly enhanced the quality, clarity, and overall impact of our manuscript. In response, we have performed additional experiments and made substantial revisions to strengthen the study. For your convenience, all revised contents have been highlighted in brown in the updated manuscript. Below, we provide a detailed, point-by-point response to each of your comments.

Reviewer #1:

1. *This manuscript is from an experienced group of investigators, and they provide a substantial amount of data. I see this as having two components, a biochemistry component of the proposed interactions between SIRT3, DsbA-1 and TFAM and the relevance of this to the development of MASH. Of the two the second component is novel.*

1) For the first component, SIRT3/DsbA-1/TFAM, SIRT3 has previously been shown to increase TFAM activity and protein levels through direct deacetylation (PMID: 30095923).

Response: Thank you for your insightful comment regarding the role of SIRT3 in regulating TFAM activity and expression via deacetylation. In the referenced study (PMID: 30095923), these experiments were performed in clear cell renal cell carcinoma (ccRCC) cells. While that work demonstrated that SIRT3 can directly deacetylate TFAM, its relevance to TFAM-mediated mtDNA release, cGAS activation, and their contributions to metabolic diseases remained unexplored. In contrast, our study provides compelling evidence that SIRT3 modulates mitochondrial biogenesis, enhances TFAM function, and prevents cytosolic mtDNA release and subsequent cGAS activation through the deacetylation of the mitochondrial chaperone protein DsbA-L. This finding reveals a distinct regulatory mechanism by which SIRT3 preserves mitochondrial integrity and suppresses inflammation. Collectively, our results suggest that SIRT3 governs mitochondrial homeostasis through a broader and more nuanced regulatory network beyond its direct interaction with TFAM. We have elaborated on this distinction in the revised manuscript (Page 10, lines 247-255).

2) Regarding the second point that this SIRT3/DsbA-1/TFAM is of relevance in MASH, unfortunately this is almost certainly an artifact caused by their use of two MASH models both of which are based on generating a state of choline deficiency. While it is true that there is no perfect MASH model, and choline deficiency models are in use, the main purpose of using two models is to use different approaches that may compensate for the other's limitations. Using two choline deficiency models is very strange. The issue if however more concrete because it is known that choline is required for the deacetylation function of SIRT3 (PMID: 35457022, PMID: 30165480). This means that many of their manipulations in which increasing SIRT3 function has a protective effect are reversing the known SIRT3 (and other enzymatic) inhibitions caused by choline deficiency. This argument is confirmed by the data from hepatocyte specific SIRT3 deficient mice which do not have increased steatosis in the setting of MASH that is not driven by choline deficiency (PMID: 22645641). The relevant point of-course is that in human MASH there is no deficiency of choline, and the proposed SIRT3/DsbA-1/TFAM pathway is likely not relevant. The

authors did discuss the fact their data from choline deficient models is the opposite of the non-choline deficient models but left the question open and suggested theier was equivalence in the two experimental approaches.

Response:

i) Addressing the concern regarding the use of choline-deficient diets and their potential confounding effect on SIRT3

We appreciate the reviewer's insightful comments on the role of choline in regulating SIRT3. To rule out confounding effects of choline deficiency, we used the Gubra-Amylin (GAN) diet, a choline-sufficient model, in SIRT3^{LKO} mice and their littermates. Similar to results from choline-deficient models, GAN-fed SIRT3^{LKO} mice exhibited enhanced hepatic lipid accumulation, increased collagen deposition, and upregulated inflammatory and fibrotic gene expression (Revised Fig. 1e–m). While previous studies have shown that choline supplementation enhances SIRT3 expression or activity, thereby alleviating HFD-induced hepatic lipid dysregulation and cardiac hypertrophy (PMID: 35457022; 30165480), our findings from the GAN-diet model suggest that the MASH phenotype resulting from SIRT3 deficiency occurs independently of choline status. Although resource limitations prevented us from applying the GAN diet to all animal models in this study, our data show that cGAS signaling is also activated in the livers of GAN diet-treated SIRT3^{LKO} mice (revised Fig. 2b). This finding supports our hypothesis that mitochondrial dysfunction-induced cGAS activation, irrespective of choline status, serves as a key mediator of MASH progression in SIRT3^{LKO} mice.

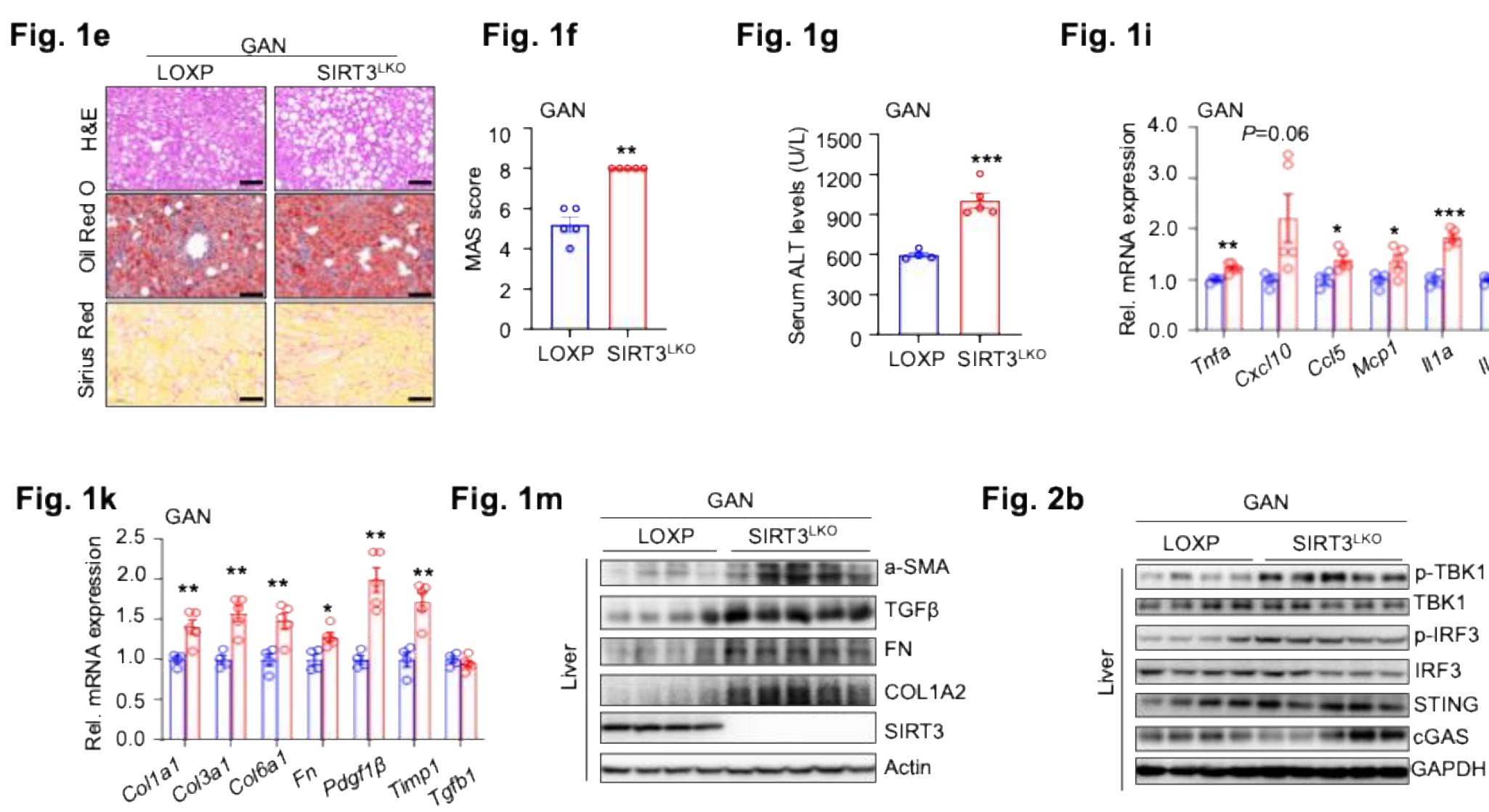

Revised Fig 1 and 2. 1e Hematoxylin & Eosin, Oil Red O, and Sirius red staining in liver sections of SIRT3^{LKO} and control loxp mice fed with GAN for 28 weeks. Scale bars, 100 μm. **1f** MAS grading score was analyzed in livers of GAN-fed SIRT3 ^{LKO} and control loxp mice. **1g** Serum ALT concentrations in GAN-fed SIRT3 ^{LKO} and control loxp mice. **1i** Relative mRNA levels of inflammatory genes in the liver of GAN-fed SIRT3^{LKO} mice compared to control mice. **1k** Relative mRNA levels of fibrosis-related genes in the livers of GAN-fed SIRT3^{LKO} and control loxp mice. **1m** Protein levels of fibrosis-related genes in the livers of GAN-fed SIRT3^{LKO} and control loxp mice. **2b** Increased cGAS signaling is observed in the livers of SIRT3^{LKO} mice fed with GAN diet. In all statistical plots, data are presented as mean ± SEM, *, $P < 0.05$; **, $P < 0.01$; ***; $P < 0.001$. Statistical significance for (**1f-1k**) was determined by unpaired, two-sided *t*-test.

An intriguing question remains: why does hepatic SIRT3 knockout exacerbate steatosis and MASH under MCD, CDAHFD, or GAN diets but not under HFD alone (PMID: 22645641)? A plausible explanation is that under milder metabolic stress (HFD), compensatory pathways may partially offset mitochondrial dysfunction caused by SIRT3 loss. In contrast, under more severe stress—diets high in fructose, sucrose, and cholesterol or low in choline—these compensations fail. Consequently, mitochondrial stress involving the DsbA-L/TFAM axis and mtDNA instability may intensify, triggering cGAS activation and accelerating MASH progression. This possibility is discussed in the revised manuscript (Page 11, Lines 289-300).

ii) Relevance to Human MASH

Choline deficiency has long been linked to hepatic lipid accumulation, and choline-deficient diets are widely used to model fatty liver and MASH in mice. However, its direct relevance to human NASH remains unclear due to limited epidemiological data. Several studies report that low choline intake correlates with worsened steatosis, fibrosis, and liver injury, while supplementation improves hepatic outcomes (PMID: 22338037, 22134222, 7590654, 25320186, 40838115, 39296030). These findings highlight the importance of choline metabolism in liver disease. Nonetheless, our data show that SIRT3 deficiency triggers cGAS activation independent of choline status, underscoring the pivotal role of the SIRT3/DsbA-L/TFAM axis in MASH pathogenesis through a choline-independent mitochondrial and innate immune mechanism.

Reviewer #2:

1. All my comments have been addressed.

Response: We sincerely appreciate your thoughtful evaluation and kind acknowledgment that all comments have been addressed. Your constructive feedback has been invaluable in improving the quality of our work.

Reviewer #3:

In our previous major concern #5, we have emphasized on the fact that there is less SIRT3 and TFAM in the input pool of proteins where DsbA-L is pulled from. The response was that equal amount of DsbA-L was pulled down under all conditions, so the co-IP should have been also equal if the interactions were not weaker. This rationale is flawed. If there is less SIRT3 and TFAM that can interact with DsbA-L, this may be the reason why less is co-IP'd with equal amounts of DsbA-L. The potential for interaction will be reduced due to the severe drop in the abundance of these interacting proteins. This must be addressed by additional experiments to show that less SIRT3 or TFAM co-IP even when the abundance of these interacting proteins is not different. The case is the same for the revised Fig. 3d (in response to point #6). An alternative option could be by inhibiting acetylation of DsbA-L and conducting IP within the same pool of protein where SIRT3 and TFAM abundance is equal between control and non-acetylated input protein.

If current results cannot be fortified further, this caveat should be included in the text (that this could be due to reduced abundance of the interacting protein).

Response: We thank the reviewer for this important and constructive comment. As suggested, we conducted a co-immunoprecipitation (Co-IP) experiment using DsbA-L knockout primary hepatocytes transfected with wild-type DsbA-L, the deacetylation-mimic mutant (K3R), or the acetylation-mimic mutant (K3Q). Our results show that the K3R mutant exhibited markedly stronger interactions with both SIRT3 and TFAM, whereas the K3Q mutant significantly weakened these interactions compared with wild-type DsbA-L (Revised Fig. 3g). These findings demonstrate that the acetylation status of DsbA-L itself directly regulates its binding affinity with SIRT3 and TFAM, independent of their abundance in the input pool. Thus, this new evidence effectively addresses the reviewer's concern and clarifies that reduced Co-IP signals primarily reflect weakened interactions caused by DsbA-L acetylation rather than differences in protein levels.

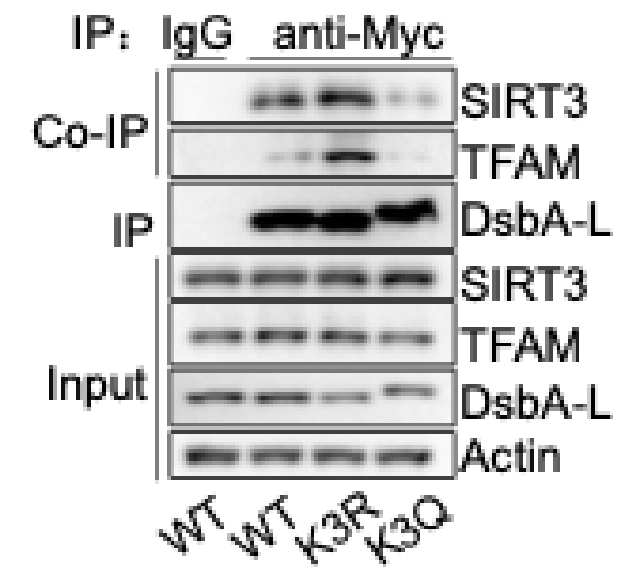

Revised Fig 3g. The interaction of DsbA-L with SIRT3 and TFAM in mouse hepatocytes overexpressing wild-type DsbA-L or K3R, K3Q DsbA-L mutants was assessed by co-immunoprecipitation. Myc-tagged DsbA-L was immunoprecipitated from cell lysates, and co-immunoprecipitated SIRT3 and TFAM were detected by Western blot.

We hope that we have satisfactorily addressed all of the reviewers' concerns and look forward to your favorable consideration of our revised manuscript.

Juli Bai and Feng Liu
